# Enzyme-Responsive Double-Locked Photodynamic Molecular Beacon for Targeted Photodynamic Anticancer Therapy

**DOI:** 10.1021/jacs.2c13732

**Published:** 2023-03-24

**Authors:** Leo K. B. Tam, Jacky C. H. Chu, Lin He, Caixia Yang, Kam-Chu Han, Peter Chi Keung Cheung, Dennis K. P. Ng, Pui-Chi Lo

**Affiliations:** † Department of Chemistry, The Chinese University of Hong Kong, Shatin, N.T., Hong Kong, China; ‡ Department of Biomedical Sciences and Tung Biomedical Sciences Centre, City University of Hong Kong, Tat Chee Avenue, Kowloon, Hong Kong, China; § Department of Clinical Pathology, Pamela Youde Nethersole Eastern Hospital, Chai Wan, Hong Kong, China; ∥ School of Life Sciences, The Chinese University of Hong Kong, Shatin, N.T., Hong Kong, China

## Abstract

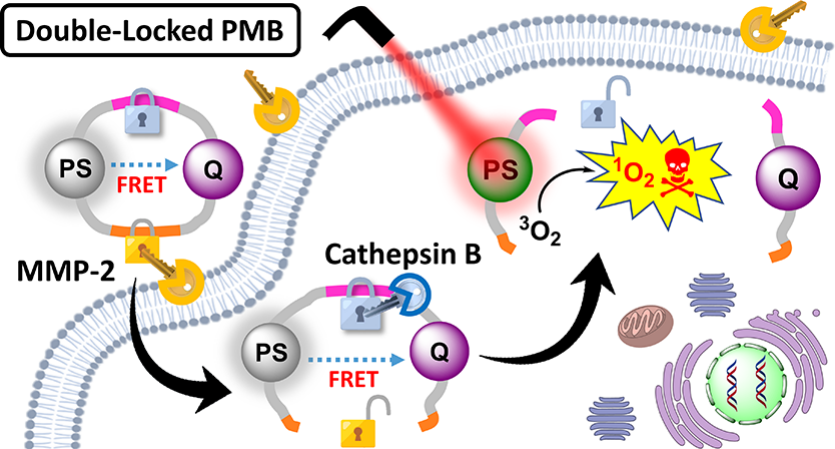

An advanced photodynamic molecular beacon (PMB) was designed and synthesized, in which a distyryl boron dipyrromethene (DSBDP)-based photosensitizer and a Black Hole Quencher 3 moiety were connected via two peptide segments containing the sequences PLGVR and GFLG, respectively, of a cyclic peptide. These two short peptide sequences are well-known substrates of matrix metalloproteinase-2 (MMP-2) and cathepsin B, respectively, both of which are overexpressed in a wide range of cancer cells either extracellularly (for MMP-2) or intracellularly (for cathepsin B). Owing to the efficient Förster resonance energy transfer between the two components, this PMB was fully quenched in the native form. Only upon interaction with both MMP-2 and cathepsin B, either in a buffer solution or in cancer cells, both of the segments were cleaved specifically, and the two components could be completely separated, thereby restoring the photodynamic activities of the DSBDP moiety. This PMB could also be activated in tumors, and it effectively suppressed the tumor growth in A549 tumor-bearing nude mice upon laser irradiation without causing notable side effects. In particular, it did not cause skin photosensitivity, which is a very common side effect of photodynamic therapy (PDT) using conventional “always-on” photosensitizers. The overall results showed that this “double-locked” PMB functioned as a biological AND logic gate that could only be unlocked by the coexistence of two tumor-associated enzymes, which could greatly enhance the tumor specificity in PDT.

## Introduction

As a promising treatment modality for cancer, photodynamic therapy (PDT) has received considerable attention due to its lower invasiveness, less side effects, and lower likelihood of occurrence of drug resistance compared to the traditional anticancer therapies.^1−3^ The treatment requires the excitation of a photosensitive drug using light with an appropriate wavelength to produce reactive oxygen species (ROS) through interactions with the endogenous oxygen. Apart from the direct attack on cancer cells and tissues, leading to necrosis, apoptosis, and/or other forms of regulated cell death,^4^ these ROS can also disrupt the tumor vasculature, resulting in tumor eradication indirectly and stimulate the host immune system.^5^ Despite the great potential, the low tumor selectivity and undesired pharmacokinetics of the currently used photosensitizers result in prolonged photosensitivity as a very common side effect of treatment.^1^ Taking the most commonly used photosensitizer for PDT of non-cutaneous solid tumors, Porfimer sodium, as an example, the residual drug that is present in all parts of the skin inevitably causes cutaneous toxicities upon exposure to sunlight.^6^ Therefore, according to the guidelines of U.S. Food and Drug Administration,^7^ all patients receiving this drug must avoid exposure of skin and eyes to direct sunlight or bright indoor light for at least 30 days (up to 90 days or more for some patients). It is envisaged that if these drawbacks can be improved, it would certainly promote the use of PDT for cancer treatment. The photosensitizing drug cetuximab saratolacan (or Akalux), which has recently been approved in Japan for the treatment of unresectable locally advanced or recurrent head and neck cancer, is a good example.^8^ It is a conjugate of the photosensitizer IR700 with the antibody cetuximab, which plays a critical role of tumor targeting toward the epidermal growth factor receptor.

Apart from the tumor-targeting issue, the oxygen-dependent nature of PDT would also affect its efficacy against tumor hypoxia, which is a common characteristic of advanced solid tumors.^9−11^ In addition, the light-dependent feature of PDT also requires an effective means of light delivery particularly to deep tissues, but the limited penetration depth of the light radiation used remains a concern.^12−14^ All these challenges greatly hinder the clinical applications of PDT. As a result, considerable efforts have been devoted to optimizing the design, properties, and efficacy of photosensitizing systems,^15−17^ replenishing the oxygen levels in the tumor microenvironment,^9−11,18−20^ and relaxing the oxygen dependence in PDT by using Type-1 photosensitizers, photodynamic oxygen economizers, the approach of mitochondrial respiration inhibition, etc.^21,22^ To circumvent the limitation of light penetration, various approaches have been extensively studied, such as extending the π conjugation of the photosensitizers to shift their absorption to red, using the two-photon strategy, X-rays with a scintillator, or chemiluminescence for excitation, and the development of upconversion photosensitizers.^12−14,18−20^

Over the years, a vast number of photosensitizers based on different organic dyes and inorganic materials have been studied,^15−17^ and various carriers have also been developed to deliver the PDT agents to the targeted sites.^19,20,23,24^ Despite the recent advances, the tumor specificity of the photodynamic action remains a challenge. Photosensitization at a nonspecific site could lead to undesired photodamage to normal cells and tissues and reduce the overall therapeutic outcome. To address this issue, various approaches have been actively explored, including the bioconjugation of photosensitizers with a tumor-directing ligand for active targeting,^25−27^ the incorporation of photosensitizers on a nanoplatform for promoting the tumor localization through the enhanced permeability and retention effect,^27−29^ and the introduction of stimuli-responsive units to photosensitizers for controlling their photodynamic activities.^30−34^ The last approach involving activatable photosensitizers is of particular interest as it can resolve the “always-on” problem of general photosensitizers and has a well-defined activation mechanism in response to the stimuli in the tumor microenvironment.

To date, most of the activatable photosensitizers reported are responsive toward a single stimulus. To further enhance the tumor specificity, a number of dual stimuli-responsive photosensitizing systems have been developed, of which the PDT efficacy can be enhanced in the presence of two stimuli.^35−49^ This concept was first reported by Ozlem and Akkaya, who prepared a boron dipyrromethene-based photosensitizer that was responsive toward both Na^+^ and H^+^ ions, functioning as a molecular AND logic gate.^50^ While most of these systems are in a nanostructured form,^35−43^ the molecular counterparts are rare, and very often they can still be partially activated in the presence of one of the stimuli.^44−49^ This approach has also been extended to nanosystems which are responsive to three stimuli.^51,52^ For the examples reported so far, the acidic environment of the tumor and the overproduced glutathione (GSH), H_2_O_2_, and enzymes in cancer cells are utilized as the stimuli. To the best of our knowledge, activatable photosensitizers using two tumor-associated enzymes as stimuli have not been reported so far, though a few dual-enzyme-activated fluorescent probes have been reported.^53−56^ We report herein such a system in a molecular form that can only be activated by the coexistence of matrix metalloproteinase-2 (MMP-2) and cathepsin B. The former is an extracellular membrane-bound enzyme which is overexpressed in most tumors, serving as an important biomarker for diagnostic and prognostic evaluation of cancer,^57^ while the latter is a lysosomal cysteine protease which is also upregulated in various malignancies.^58^ This “double-locked” photodynamic molecular beacon (PMB) is largely quenched in the native form and even in the presence of one of these enzymes, and it can only be unlocked or activated in the presence of both of these cancer-associated enzymes. This advanced feature renders this PMB acting as an enzymatic AND logic gate and functioning as a precise photosensitizer for targeted PDT against cancer, as demonstrated through a series of experiments using a range of cancer cell lines and tumor-bearing nude mice.

## Results and Discussion

### Molecular Design and Mechanistic Action

**Figure 1:**
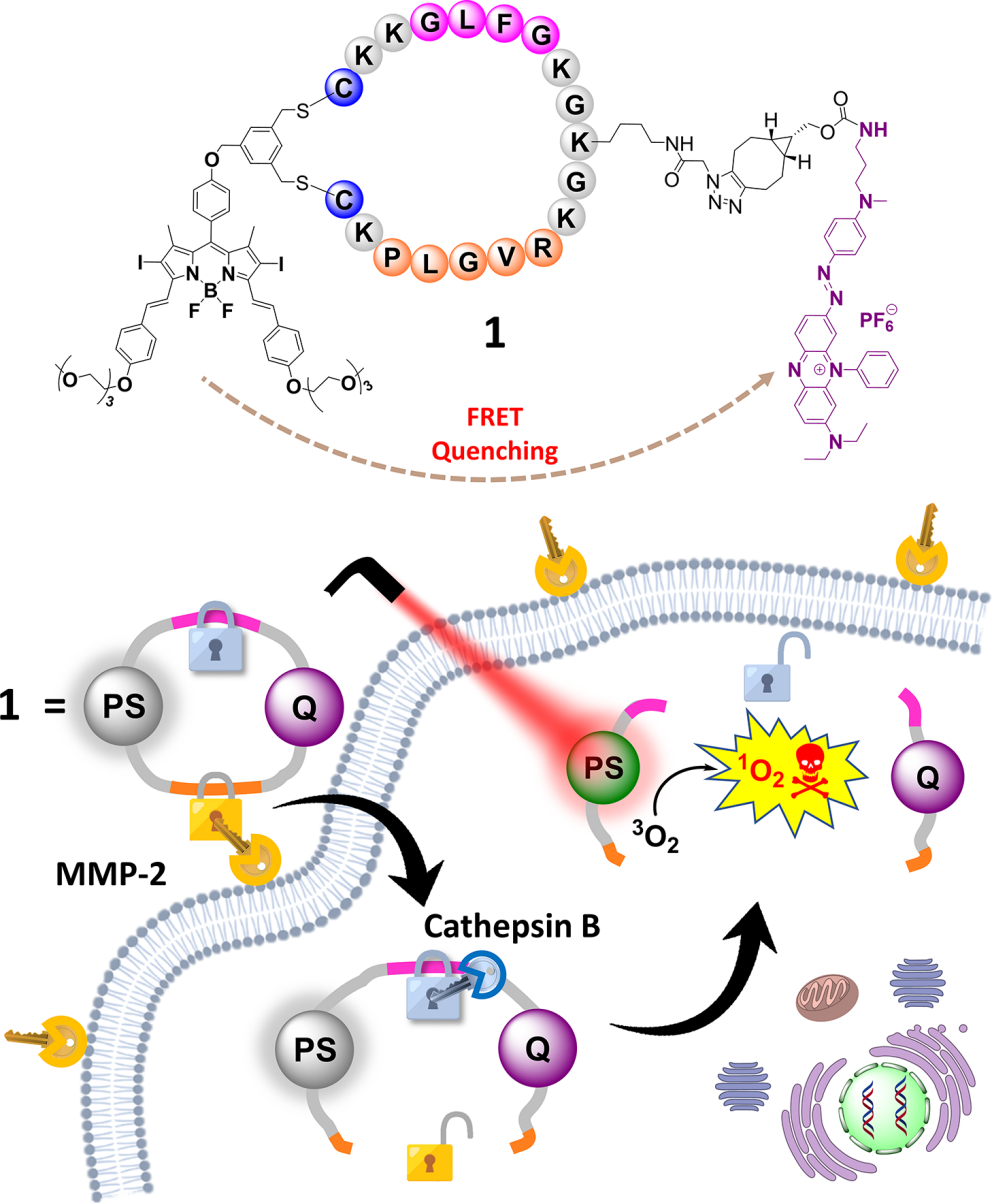
Molecular structure of the double-locked PMB **1** and its dual-enzyme-unlocked mechanism.

Figure 1 shows the molecular structure of this double-locked PMB (compound **1**) and its dual-enzyme-activated mechanism in cancer cells. The compound contains a distyryl boron dipyrromethene (DSBDP)-based photosensitizer and a Black Hole Quencher 3 (BHQ-3) moiety, which are connected via two peptide segments of a cyclic peptide. The segments contain the peptide sequences PLGVR and GFLG, which are the respective substrates of MMP-2 and cathepsin B.^59,60^ Having a BHQ-3 moiety in proximity, the DSBDP unit is fully quenched in terms of fluorescence emission and ROS generation due to the presence of a highly efficient Förster resonance energy transfer (FRET) process. Upon interactions with the overexpressed extracellular MMP-2 of cancer cells, followed by endocytosis and further interactions with the overproduced cathepsin B in the lysosomes, both of the peptide segments are cleaved to completely separate the two components. As a result, the free DSBDP unit becomes activated and can restore its photodynamic activities upon light irradiation. In the presence of only one of these stimuli, the two components are still connected, making the partially cleaved products remain largely quenched. Hence, this double-locked PMB can only be unlocked by these extracellular and intracellular keys, which is supposed to be in a sequential manner, and this design can greatly enhance the tumor specificity of the treatment.

### Synthesis and Characterization

To prepare the cyclic peptide skeleton of PMB **1**, the azide-appended peptide resin **2** was first prepared using a modified 9-fluorenylmethoxycarbonyl (Fmoc) solid-phase peptide synthesis (SPPS) protocol with the commercially available *N*-α-Fmoc-protected amino acids and Fmoc-Lys(N_3_)–OH^61^ as the building blocks and rink amide resin as the solid support (Scheme S1 in the Supporting Information). The Fmoc-protecting group was removed by 20% piperidine in *N*,*N*-dimethylformamide (DMF), while the carboxyl group was activated by 1-[bis(dimethylamino)methylene]-1*H*-1,2,3-triazolo[4,5-*b*]pyridinium 3-oxid hexafluorophosphate (HATU) and *N*,*N*-diisopropylethylamine (DIPEA) for each coupling. Resin **2** was then treated with 20% piperidine in DMF to remove the Fmoc-protecting group and then with a mixture of trifluoroacetic acid (TFA), triisopropylsilane (TIPS), and water (95:2.5:2.5 v/v/v) to remove all other protecting groups and detach the peptide chain from the resin. The resulting unprotected azide-appended peptide **3** was purified by reverse-phase high-performance liquid chromatography (HPLC) and characterized by electrospray ionization (ESI) mass spectrometry. It is worth mentioning that the introduction of an azide group in the middle of the linear peptide with two terminal cysteine residues is the key to the synthesis of the PMB that can place the photosensitizer and the quencher on two sides of the cyclic peptide. This point will be elaborated below.

For comparison, a non-cleavable analogue of PMB **1** was also prepared, which required a peptide precursor in which both the PLGVR and GFLG sequences were replaced with an oligoglycine chain with the same number of amino acid residues. By using the same synthetic procedure described above, resin **4** and peptide **5**, i.e., the non-cleavable analogues of resin **2** and peptide **3**, respectively, were prepared (Scheme S2) and characterized.

For the photosensitizing component, a diiodo DSBDP was used because of the superior photosensitizing properties of this class of compounds.^62^ Treatment of our previously reported hydroxy DSBDP **6**(63) with an excess of 1,3,5-tris(bromomethyl)benzene (**7**) in the presence of K_2_CO_3_ in DMF gave the mono-substituted product **8** in a 35% yield (Scheme S3). The 3,5-bis(bromomethyl)phenyl substituent was introduced to facilitate the subsequent cyclic peptide formation through disubstitution with two cysteine residues of the linear peptide.^64^ For the quenching component, BHQ-3 was conjugated with a bicyclo[6.1.0]non-4-yne (BCN) moiety to facilitate the coupling with the azide-modified cyclic peptide via strain-promoted azide–alkyne cycloaddition (SPAAC).^65^ As shown in Scheme S4, treatment of the commercially available BHQ-3 amine **9** with 4-nitrophenyl carbonate-modified BCN **10**(66) and triethylamine gave BCN-substituted BHQ-3 **11**, which was isolated by reverse-phase HPLC in a 65% yield. Both **8** and **11** were characterized with ^1^H and ^13^C NMR spectroscopy and high-resolution ESI mass spectrometry.

**Figure 2:**
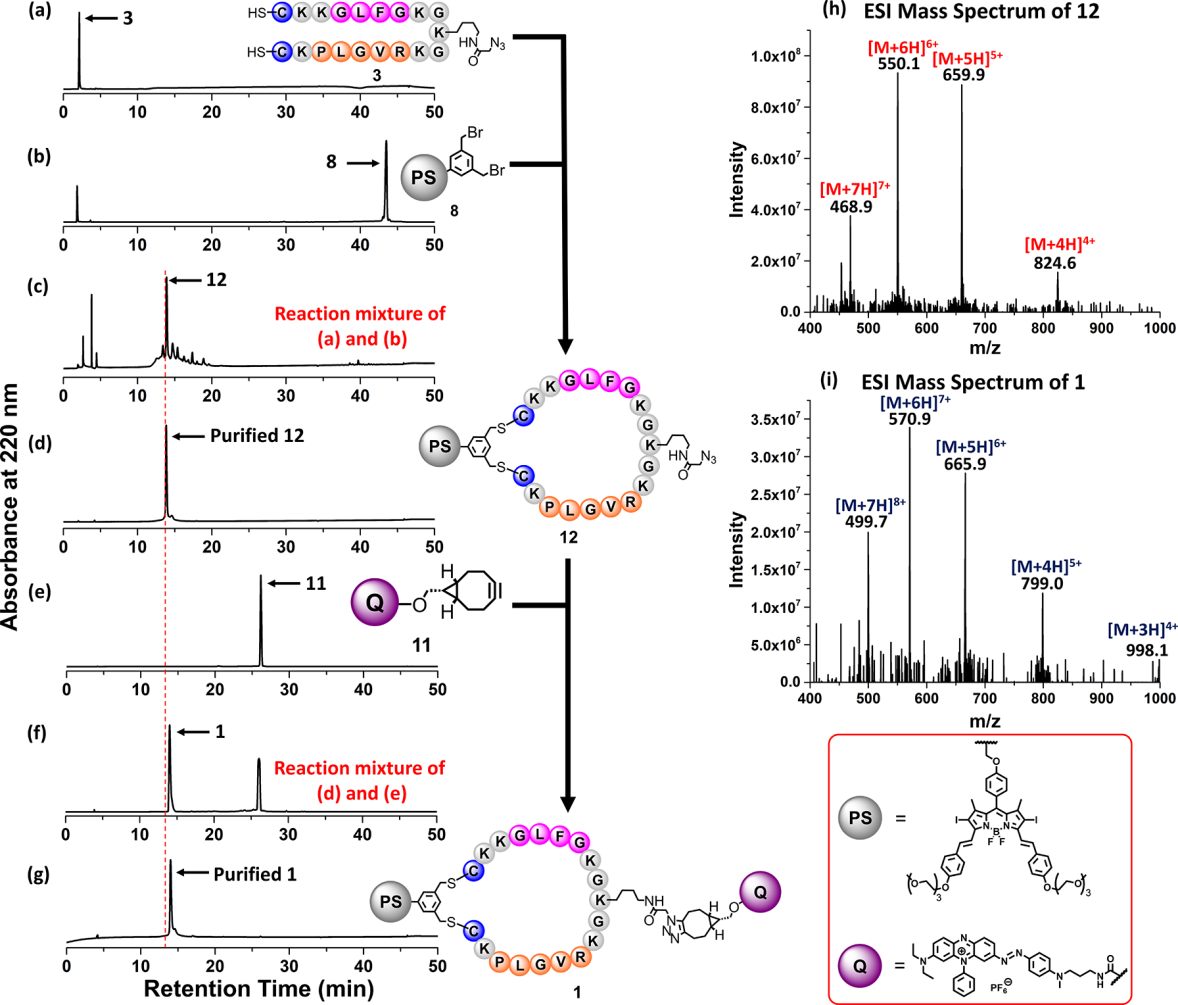
Monitoring the course of the formation of PMB **1** using LC–MS.

**Scheme 1:**
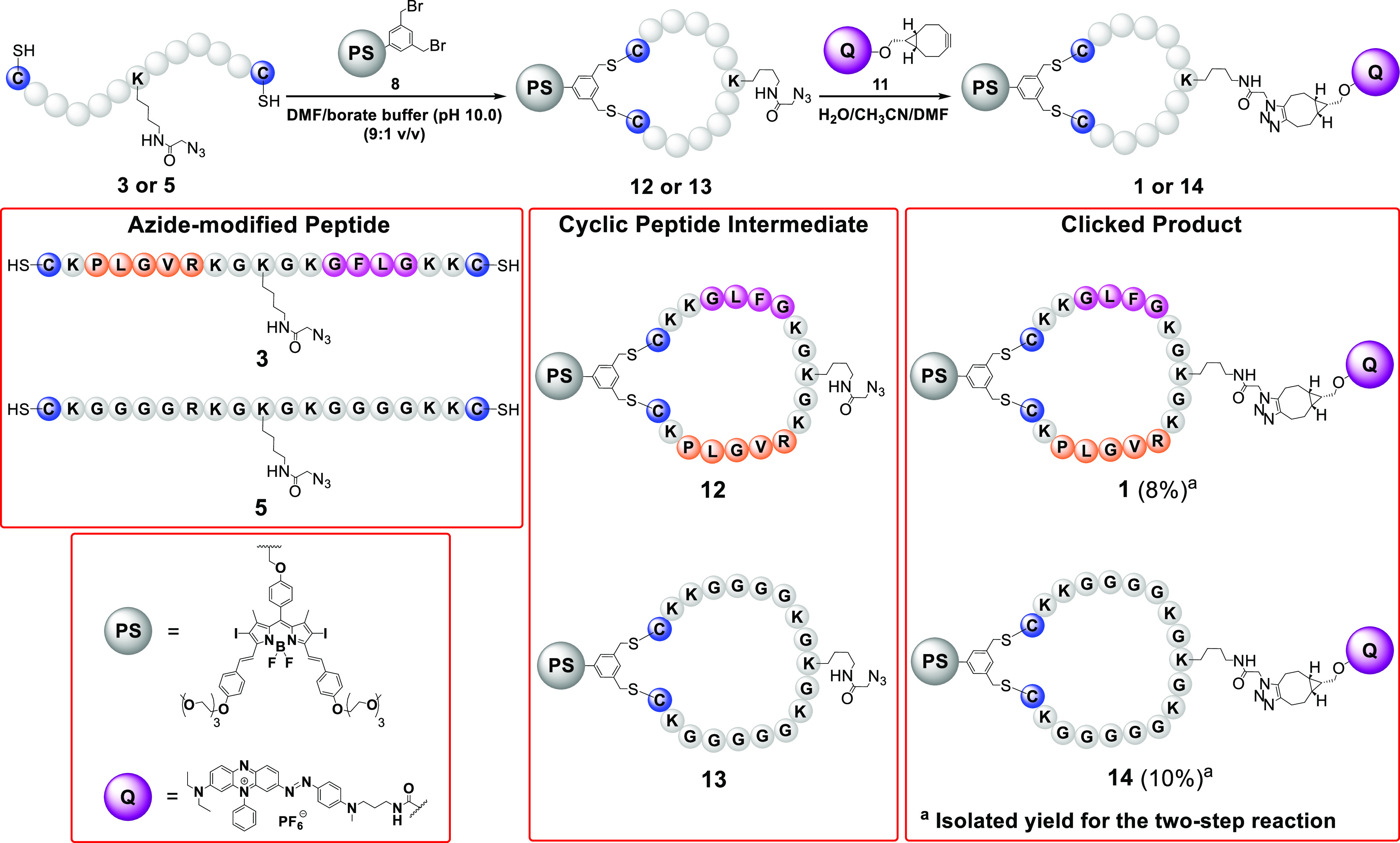
Synthetic Route of PMB **1** and Its Non-cleavable Analogue **14**

After preparing these building blocks, PMB **1** was assembled covalently according to Scheme 1, and the course of the reaction was monitored step-by-step by liquid chromatography–mass spectrometry (LC–MS) (Figure 2). The azide-modified linear peptide **3** (1 mM) was first treated with DSBDP **8** (1 mM) in a mixture of DMF and borate buffer (pH 10.0) (9:1 v/v) to induce cyclic peptide formation according to our previously reported synthetic methodology.^64^ After stirring the mixture at room temperature for 15 min, the reaction was essentially completed as indicated by the disappearance of the signals of the two starting materials and the appearance of a new intense signal at 13.5 min in the chromatogram (Figure 2a–c). After purification, this intermediate product was confirmed by ESI mass spectrometry (Figure 2d,h) to be the cyclic peptide **12**, which was formed via disubstitution of **8** with the two cysteine residues of **3**. Despite the long peptide sequence with 19 amino acid residues, the cyclization proceeded smoothly, giving the cyclic peptide **12** in a 70% HPLC yield, though a significant amount of sample was lost during the purification. The purified sample (in H_2_O/CH_3_CN) was then treated with BCN-substituted quencher **11** in DMF (0.5 mM). After 1 h, a new signal at 14 min appeared, while the signal of **12** (at 13.5 min) could not be seen in the chromatogram (Figure 2d–f). This fraction was then purified and characterized by ESI mass spectrometry (Figure 2g,i), which confirmed that it was the coupled product **1**, which was formed via SPAAC^65^ and isolated in an 8% overall yield through the two-step process.

For comparison, a non-cleavable analogue of PMB **1** was prepared similarly. Treatment of the linear peptide **5** with DSBDP **8** gave the cyclic peptide intermediate **13**, which was then treated with **11** to afford the clicked product **14** in a 10% isolated yield. The course of its formation was also monitored by LC–MS (Figure S1).

### Electronic Absorption and Photophysical Properties

The electronic absorption and fluorescence spectra of PMB **1** were first recorded in DMF and compared with those of the components **8** and **11**, as well as the non-cleavable analogue **14** (Figure S2 and Table 1). The absorption spectra of **1** and **14** resembled each other, and as expected, they showed the absorption features of DSBDP **8** and BHQ-3 **11**, including an intense and broad absorption at 662 nm, a shoulder at 610 nm, and several high-energy bands at ca. 320, 380, and 450 nm. The similar absorption positions suggested that the two chromophores do not exhibit significant ground-state interactions. In contrast, the fluorescence spectra of **1** and **14** were remarkably different from that of DSBDP **8**. While a strong fluorescence band was observed for the latter at 689 nm with a fluorescence quantum yield (Φ_F_) of 0.22 relative to the unsubstituted zinc(II) phthalocyanine (ZnPc) (Φ_F_ = 0.28), the fluorescence band was very weak for **1** and **14** with a Φ_F_ value of 0.01, which was clearly due to the efficient FRET-based quenching by the BHQ-3 moiety.

**Table 1:** Electronic Absorption and Photophysical Data for **1**, **8**, **11**, and **14** in DMF

compound	λ_abs_ (nm) (log ε)	λ_em_^a^ (nm)	Φ_F_^b^	Φ_Δ_^c^
**1**	323 (4.41), 377 (4.57), 447 (4.17), 610 (4.64), 662 (4.88)	693	0.01	0.02
**8**	320 (4.51), 377 (4.59), 465 (4.15), 610 (4.41), 660 (4.79)	689	0.22	0.52
**11**	391 (4.04), 620 (4.59), 662 (4.66)			
**14**	322 (4.61), 387 (4.64), 447 (4.30), 610 (4.64), 662 (4.86)	693	0.01	0.02

a Excited at 610 nm.

b Relative to ZnPc (Φ_F_ = 0.28 in DMF).

c Relative to ZnPc (Φ_Δ_ = 0.56 in DMF).

**Figure 3:**
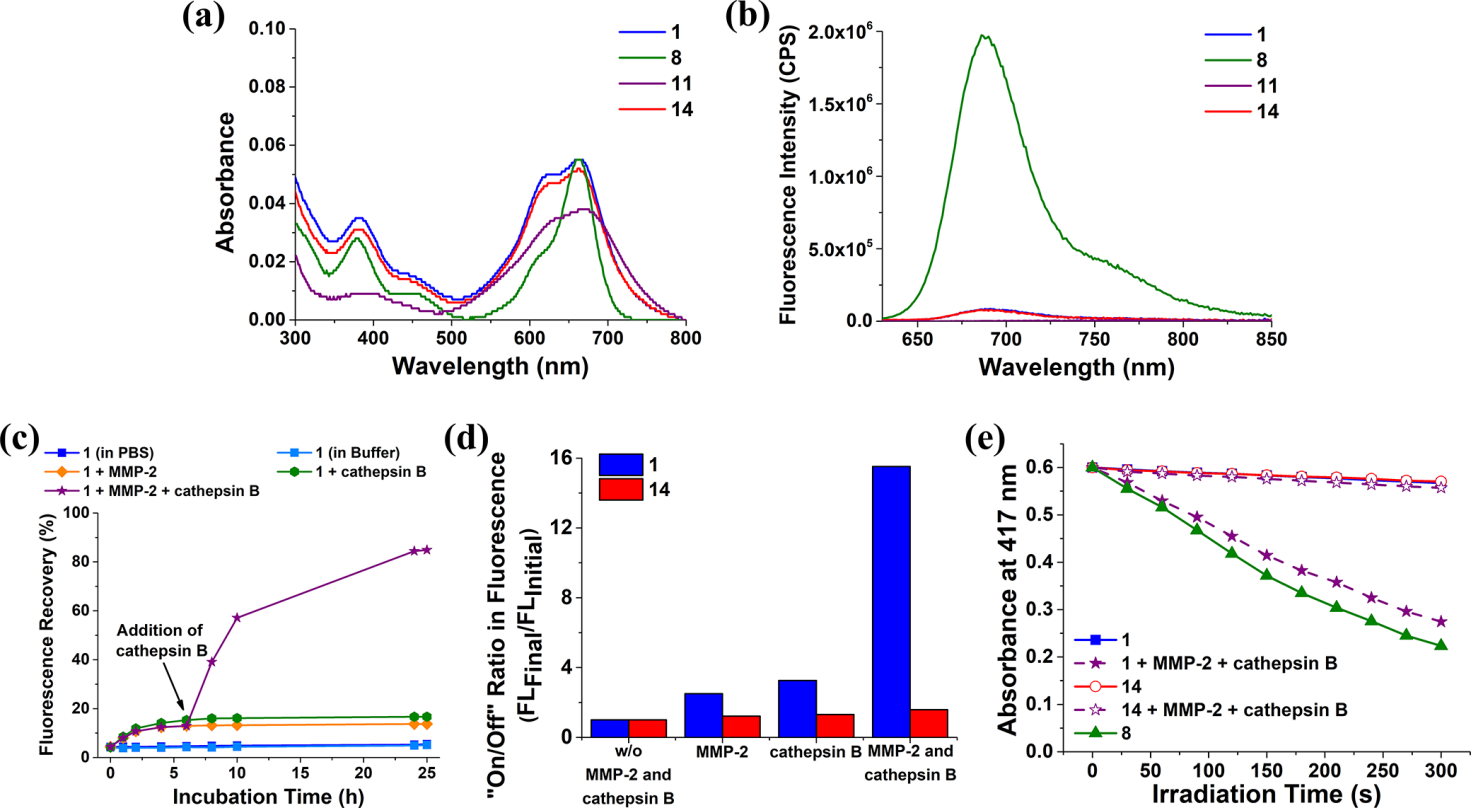
(a) Electronic absorption and (b) fluorescence (λ_ex_ = 610 nm) spectra of **1**, **8**, **11**, and **14** (all at 1 μM) in PBS at pH 7.4 with 0.1% Tween 80 (v/v). (c) Fluorescence recovery for **1** (1 μM) in the absence of MMP-2 and cathepsin B, upon treatment with either MMP-2 (2 μg mL^–1^) or cathepsin B (1 unit mL^–1^) at 37 °C over a period of 25 h or upon treatment with MMP-2 (2 μg mL^–1^) for 6 h and then with cathepsin B (1 unit mL^–1^) for a further 19 h at 37 °C. The solvent was either PBS at pH 7.4 (for the study involving MMP-2) or buffer solution (pH 5.0, 25 mM NaOAc, 1 mM EDTA, 500 μM GSH) (for the study involving cathepsin B), both in the presence of 0.1% Tween 80 (v/v). (d) On/off ratios for **1** and **14** under different conditions as determined by dividing the corresponding fluorescence intensity at 25 h by the initial fluorescence intensity. (e) Comparison of the rates of decay of DPBF (initial concentration = 30 μM) sensitized by **1** and **14** (both at 1 μM), both with and without the sequential treatment with MMP-2 and cathepsin B as described above, followed by light irradiation (λ > 610 nm) for 300 s. The results for **8** (1 μM) in the buffer are also included for comparison.

The spectral properties of these four compounds were then studied in phosphate-buffered saline (PBS) at pH 7.4 in the presence of 0.1% Tween 80 (v/v) added to increase the solubility and reduce the aggregation of the compounds in this aqueous medium. As shown in Figure 3a, the longest-wavelength absorption was slightly broadened and diminished for all of the compounds compared with that observed in DMF, reflecting their higher aggregation tendency in this medium. Upon excitation at 610 nm, DSBDP **8** showed a very strong fluorescence emission at 690 nm, while the fluorescence band of **1** and **14** was greatly reduced and that of BHQ-3 **11** could not be detected (Figure 3b). The results were essentially the same as those obtained in DMF. Similarly, we also recorded the spectra in the working buffer of cathepsin B, i.e., pH 5.0 with 25 mM sodium acetate (NaOAc), 1 mM ethylenediaminetetraacetic acid (EDTA), and 500 μM GSH, also in the presence of 0.1% Tween 80 (v/v). The spectra as shown in Figure S3 were very similar to those recorded in PBS.

The singlet oxygen generation efficiency of **1** was then studied and compared with that of **8**, **14**, and ZnPc in all these media using 1,3-diphenylisobenzofuran (DPBF) as the singlet oxygen probe.^67^ The absorbance of DPBF’s absorption at 415 nm (in DMF) or 417 nm (in PBS and the buffer) was monitored during light irradiation (λ > 610 nm). Figures S4–S6 show the changes in the absorption spectra of the mixtures in DMF, PBS, and the buffer, respectively, upon irradiation. It can be seen that in all these media while **8** and ZnPc could effectively sensitize the formation of singlet oxygen to induce substantial decay of DPBF, the spectra were not significantly changed for **1** and **14**, showing that the photosensitizing property of these two conjugates was also largely quenched. The results are summarized in Figure S7. The singlet oxygen quantum yields (Φ_Δ_) were also determined in DMF. The value of **8** (Φ_Δ_ = 0.52) was comparable with that of ZnPc used as the reference (Φ_Δ_ = 0.56) and was much higher than those of **1** and **14** (Φ_Δ_ = 0.02) (Table 1).

### MMP-2- and Cathepsin B-Responsive Behavior

To examine the response of PMB **1** toward MMP-2 and cathepsin B, its absorption and fluorescence spectra were monitored in the presence of these stimuli at 37 °C over a period of 25 h, using the non-cleavable analogue **14** as the negative control. For studying the MMP-2-responsive effect, MMP-2 (2 μg mL^–1^) in PBS at pH 7.4 was used to mimic the high serum levels of this enzyme in patients with cancer.^68^ The effect of cathepsin B (using a concentration of 1 unit mL^–1^) was studied in the working buffer mentioned above at pH 5.0 to mimic the lysosomal environment.^69^ As shown in Figure S8a–d, while the absorption spectrum of **1** in PBS, both in the absence and presence of MMP-2, was not significantly changed over 25 h, the fluorescence intensity was slightly increased and reached a plateau after 6 h when MMP-2 was present. Similar results were obtained for the study using cathepsin B in the buffer (Figure S8e–h). The results showed that in the absence of these enzymes, PMB **1** was stable under these conditions. In the presence of one of these enzymes, the fluorescence emission of **1** remained significantly quenched as the DSBDP unit was still connected to the BHQ-3 moiety despite the cleavage of one of the peptide linkers. The more flexible ring-opening structure, however, might slightly increase the separation of the two moieties, leading to a lower FRET efficiency,^70^ which could explain the small increase in fluorescence intensity. For comparison, the effect of these enzymes was also studied for **14**. As expected, both the absorption and fluorescence spectra were not changed toward these two enzymes (Figure S9).

To study the dual-enzyme-activated effect, PMB **1** was first treated with MMP-2 (2 μg mL^–1^) in PBS for 6 h, followed by the addition of NaOAc, EDTA, and GSH and adjustment of the pH to 5.0 to create a working environment for cathepsin B. This enzyme (1 unit mL^–1^) was then added, and the mixture was stirred for a further 19 h. As expected, after the addition of cathepsin B, although the longest-wavelength absorption was just slightly intensified, the intensity of the fluorescence band was largely increased. The intensity at 25 h was almost the same as that at 24 h, which could be regarded as full activation, arising from the complete separation of the two components. In contrast, the absorption and fluorescence spectra of **14** remained unchanged upon this treatment (Figure S10). Hence, PMB **1** functioned as an AND logic gate that could be turned on by the coexistence of these two enzymes.

The fluorescence recovery was then determined for **1** under the aforementioned conditions, assuming that the fluorescence intensity of **8** at the same concentration in the same medium was the maximum fluorescence intensity that could be recovered. The results are depicted in Figure 3c. It was found that the fluorescence recovery was 13% and 16% after the treatment with MMP-2 only and cathepsin B only, respectively, for 25 h. Interestingly, the value could reach 85% after the treatment with both enzymes. In addition, we also determined the corresponding on/off ratios by dividing the fluorescence intensity at 25 h by the initial fluorescence intensity under each of these conditions. As shown in Figure 3d, which also contains the results for the non-cleavable analogue **14** for comparison, the dual-enzyme-activated condition could lead to an on/off ratio of 16 for **1**, while the ratios were just ca. 2.7 for the single-enzyme-activated conditions. For **14**, the ratios were below 1.5 under all the conditions, showing that the effect of the two enzymes was negligible for this conjugate.

Apart from the activation in fluorescence emission, the activation of **1** in singlet oxygen generation was also studied. PMB **1** (1 μM) was treated with MMP-2 and/or cathepsin B as described above and then mixed with DPBF (30 μM). The absorption spectra of these mixtures were monitored during light irradiation (λ > 610 nm) over a period of 300 s. As shown in Figure S11, the absorption band of DPBF at 417 nm was slightly diminished during irradiation for the treatment groups of MMP-2 only and cathepsin B only. The decrease in absorbance became more substantial for **1** after the treatment with both enzymes. As expected, the changes were negligible for **14** under all conditions (Figure S12). Figure 3e shows the change in the absorbance of DPBF’s absorption at 417 nm with the irradiation time for **1** and **14**, both with and without the sequential treatment with MMP-2 and cathepsin B, which could reflect the singlet oxygen generation efficiency. It can be seen that after the dual activation, the photosensitizing property of **1** was turned on remarkably, and the singlet oxygen generation efficiency was just slightly lower than that of DSBDP **8**.

**Figure 4:**
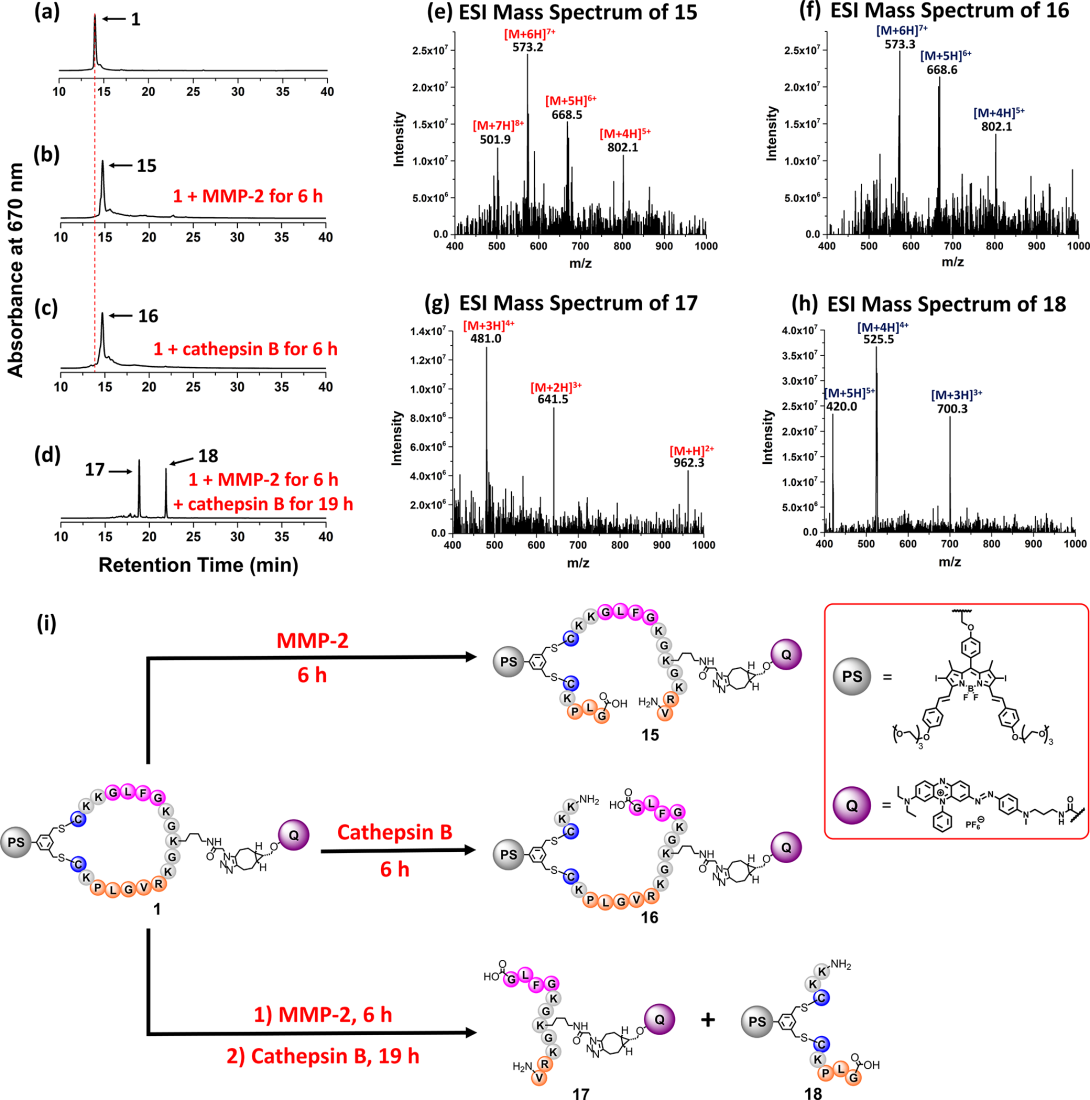
HPLC chromatograms of (a) **1** and the reaction mixtures of (b) **1** after the treatment with MMP-2 for 6 h, (c) **1** after the treatment with cathepsin B for 6 h, and (d) **1** after the treatment with MMP-2 for 6 h and then with cathepsin B for 19 h. ESI mass spectra of (e) **15**, (f) **16**, (g) **17**, and (h) **18**. (i) Enzymatic reactions of **1** with MMP-2 and/or cathepsin B.

To further confirm that the restoration of fluorescence emission and singlet oxygen generation of PMB **1** was due to the cleavage by the enzymes, LC–MS was used to analyze the reaction mixtures (Figure 4). After **1** (1 μM) had been treated with MMP-2 (2 μg mL^–1^) in PBS for 6 h, the signal of **1** (with the retention time at 14.0 min) disappeared, while a new intense signal at 14.7 min was observed in the chromatogram of the mixture. Similarly, for the mixture obtained after treating **1** (1 μM) with cathepsin B (1 unit mL^–1^) in the working buffer at pH 5.0 for 6 h, a new signal with virtually the same retention time was detected in the chromatogram (Figure 4a–c). The ESI mass spectra of the two new fractions, labeled as **15** and **16**, respectively, were found to be very similar (Figure 4e,f), giving a series of signals corresponding to the multi-protonated molecular ions. The molecular mass was identical for both compounds, which was larger than that of **1** by 18, suggesting that one of the amide bonds of **1** was cleaved by enzymatic hydrolysis. Since it has been reported that the favorable site for cleavage of PLGVR and GFLG by the corresponding enzymes is PLGVR and GFLG (the underlined), respectively,^71,72^ the structures of **15** and **16** are proposed as indicated in Figure 4i, for which the DSBDP and BHQ-3 moieties are still linked up together.

To study the dual-enzyme-activated effect, PMB **1** was treated with MMP-2 (for 6 h) and cathepsin B (for 19 h) sequentially as mentioned above. The chromatogram of the mixture showed the disappearance of the signal of **1** and the occurrence of two new signals at 18.8 and 21.8 min (Figure 4d). The mass spectral data of these two fractions showed that they were the BHQ-3 and DSBDP fragments **17** and **18**, respectively, which were formed by cleaving the two peptide segments at the aforementioned sites (Figure 4g–i). These results clearly showed that **1** was cleaved by the two enzymes specifically, and the detachment of the BHQ-3 quencher restored the fluorescence emission and singlet oxygen production of the DSBDP moiety.

### Cellular Uptake and Intracellular Activation in Fluorescence Emission

**Figure 5:**
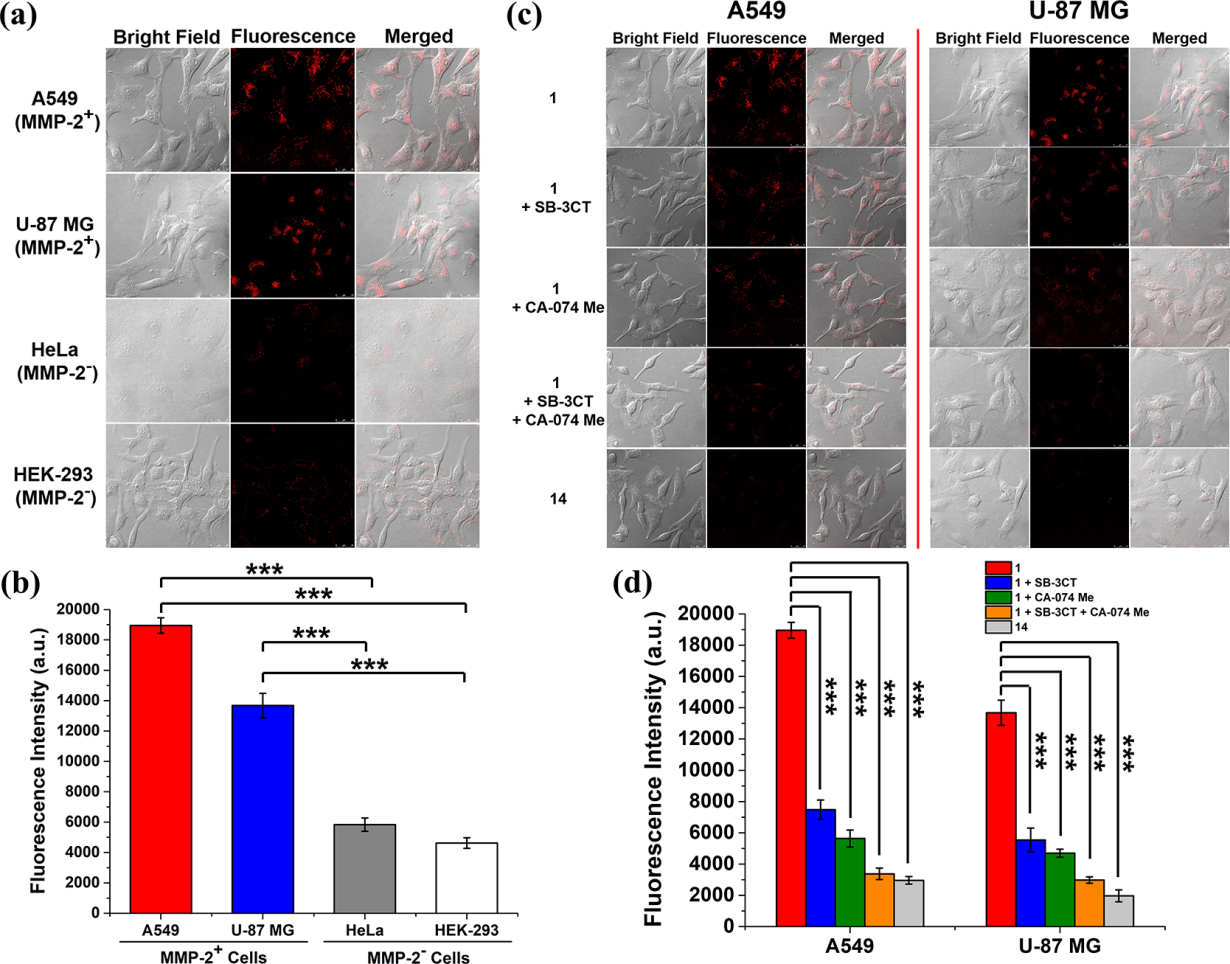
(a) Bright field, fluorescence, and the merged images of A549, U-87 MG, HeLa, and HEK-293 cells after incubation with **1** (2 μM) in a serum-free medium for 1 h, followed by incubation in the neat medium for a further 6 h. (b) Mean intracellular fluorescence intensities of A549, U-87 MG, HeLa, and HEK-293 cells under these conditions as determined by flow cytometry. Data are expressed as the mean ± standard error of the mean (SEM) of three independent experiments. (c) Bright field, fluorescence, and the merged images of A549 and U-87 MG cells after incubation in a serum-free medium in the absence or presence of SB-3CT (10 μM) and/or CA-074 Me (25 μM) for 2 h, and then with **1** (2 μM) for 1 h, followed by post-incubation in the medium for a further 6 h or incubation with **14** (2 μM) for 1 h, followed by post-incubation in the medium for a further 6 h. (d) Mean intracellular fluorescence intensities of A549 and U-87 MG cells under these conditions as determined by flow cytometry. Data are expressed as the mean ± SEM of three independent experiments. ***p < 0.001 as calculated by the Student’s *t*-test.

The cellular uptake of **1** was studied using a range of cell lines with different expression levels of MMP-2, namely the MMP-2-overexpressed A549 human lung carcinoma cells^73^ and U-87 MG human glioblastoma cells,^74^ as well as the low MMP-2-expressed HeLa human cervical carcinoma cells^75^ and HEK-293 human embryonic kidney normal cells.^76^ The cells were incubated with **1** (2 μM) in a serum-free medium for 1 h and then in the neat medium for a further 6 h to provide sufficient time for enzymatic cleavage. As shown in Figure 5a, the intracellular fluorescence intensity as observed by confocal microscopy was significantly higher for the MMP-2-positive A549 and U-87 MG cells compared with that for the MMP-2-negative HeLa and HEK-293 cells. Similar results were obtained by flow cytometry (Figure 5b). The quantified fluorescence intensity for A549 cells was 3.8-fold that for HEK-293 cells. This observation could be explained by the enhanced MMP-2 levels in the MMP-2-positive cells that could promote the cleavage of the PLGVR linker. After subsequent cleavage of the remaining GFLG linker by the intrinsic cathepsin B inside the cells, the probe was activated favorably, leading to higher intracellular fluorescence intensity. The very weak intensity for the MMP-2-negative cells showed that the probe was still significantly quenched even when one of the peptide linkers (i.e., the GFLG sequence) was cleaved by the intracellular cathepsin B.

The above hypothesis was verified by further studying the intracellular activation of **1** using two enzyme inhibitors, namely SB-3CT, which is a potent and competitive matrix metalloproteinase inhibitor that can reversibly inhibit the enzymatic function of MMP-2,^77^ and CA-074 methyl ester (CA-074 Me), which is a cell-permeable and selective inhibitor of cathepsin B.^78^ The cells were pretreated with SB-3CT (10 μM) and/or CA-074 Me (25 μM) or simply the neat medium for 2 h, followed by incubation with **1** (2 μM) for a further 1 h. After being rinsed with PBS, the cells were incubated in the medium for a further 6 h. As a negative control, the cells were simply incubated with **14** (2 μM) for 1 h, followed by post-incubation in the medium for 6 h. The fluorescence images of the cells under all these conditions were captured using a confocal laser scanning microscope. For the two MMP-2-positive cell lines (Figure 5c), bright intracellular fluorescence was observed for **1** without pretreating the cells with the two inhibitors. The intensity was significantly reduced when the cells were pretreated with one of the two inhibitors. For the cells with pretreatment with both inhibitors, fluorescence due to **1** could hardly be seen. Similarly, for the cells being incubated with **14**, the intracellular fluorescence was negligible. These results were in good agreement with those obtained by flow cytometry (Figure 5d). Again, for PMB **1**, the fluorescence intensity was the highest for the cells without being pretreated with the two inhibitors. For A549 cells, the intensity was 5.6-fold that for the cells being pretreated with the two inhibitors. The slightly higher intensity for the SB-3CT-pretreated cells compared with that for the CA-074 Me-pretreated cells suggested that the activation effect of cathepsin B was slightly higher than that of MMP-2, which was consistent with the results of the solution study (Figure 3c,d). The results for the two MMP-2-negative cell lines were similar, but the activation effect was significantly smaller (Figure S13). For HeLa cells being treated with **1**, the fluorescence intensity of the native cells (i.e., the dual activated condition) was just 3.2-fold that of the cells under the dual inhibited condition. The overall results further demonstrated that the fluorescence emission of PMB **1** could only be remarkably activated upon interactions with MMP-2 and cathepsin B through cleavage of the corresponding peptide substrates.

### Subcellular Localization

The subcellular localization of **1** in A549 cells was also studied. After incubation with **1** (2 μM) for 1 h and post-incubation in culture medium for a further 6 h, the cells were stained with LysoTracker Green DND-26 (2 μM for 30 min), MitoTracker Green FM (0.2 μM for 15 min), or ER-Tracker Green (1 μM for 15 min). The intracellular fluorescence of the DSBDP fragment of **1** formed through the enzymatic cleavage and the trackers were examined using confocal microscopy. As shown in Figure S14, the fluorescence due to the fragment of **1** could only be overlapped with that of LysoTracker but not the other two trackers, showing that **1**, after the enzymatic activation, was localized mainly in the lysosomes, which are the last compartments of the endocytic pathway and could facilitate the activation by cathepsin B therein.

### Intracellular Activation in ROS Generation and Cytotoxicity

**Figure 6:**
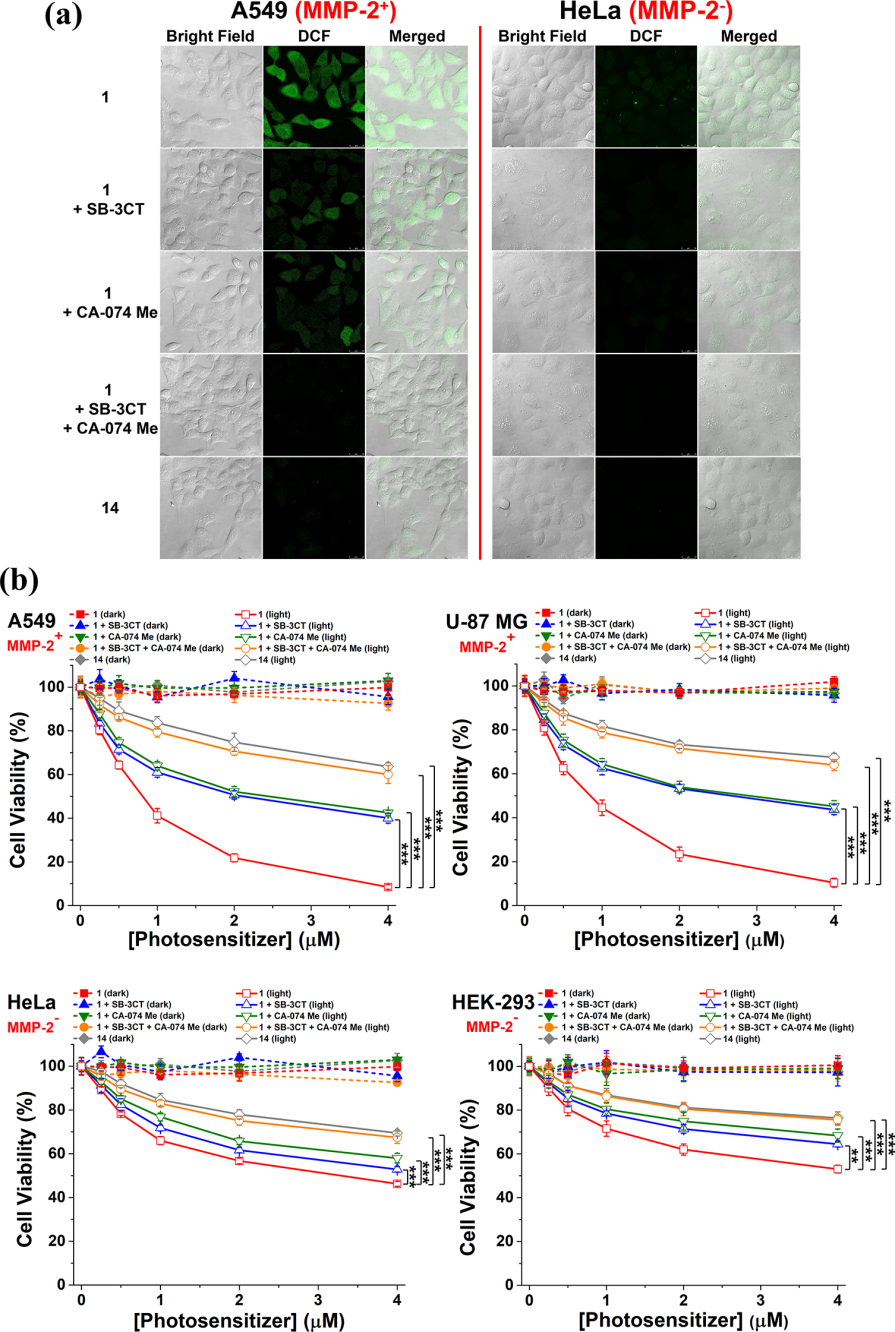
(a) Intracellular ROS generation as reflected by the intracellular fluorescence intensity of DCF. A549 and HeLa cells were incubated in a serum-free medium in the absence or presence of SB-3CT (10 μM) and/or CA-074 Me (25 μM) for 2 h, and then with **1** (2 μM) for 1 h, or simply with **14** (2 μM) for 1 h. After post-incubation in the medium for 6 h, the cells were incubated with H_2_DCFDA (10 μM) for 30 min, followed by dark or light (λ > 610 nm, 23 mW cm^–2^, 14 J cm^–2^) treatment. (b) Dark and photo (λ > 610 nm, 23 mW cm^–2^, 28 J cm^–2^) cytotoxicity of **1** and **14** against A549, U-87 MG, HeLa, and HEK-293 cells under the different conditions as described. Data are expressed as the mean ± SEM of three independent experiments, each performed in quadruplicate. **p < 0.01 and ***p < 0.001 as calculated by the Student’s *t*-test.

The enzyme-responsive intracellular ROS generation of **1** was then examined in A549 and HeLa cells using 2′,7′-dichlorodihydrofluorescein diacetate (H_2_DCFDA) as the ROS fluorescent probe. Once H_2_DCFDA is internalized, it will be deacetylated by the intracellular esterase, followed by oxidation by the intracellular ROS, leading to the formation of the highly fluorescent 2′,7′-dichlorofluorescein (DCF).^79^ The cells were first pretreated with SB-3CT (10 μM) and/or CA-074 Me (25 μM) or simply the neat medium for 2 h, followed by incubation with **1** (2 μM) for a further 1 h. As a negative control, the cells were simply incubated with **14** (2 μM) for 1 h. After being rinsed with PBS, the cells were incubated in the medium for a further 6 h. Finally, the cells were incubated with H_2_DCFDA (10 μM) for 30 min, followed by dark or light (λ > 610 nm, 23 mW cm^–2^, 14 J cm^–2^) treatment. Figure 6a shows the corresponding confocal fluorescence images. It can be seen that for the MMP-2-positive A549 cells, the fluorescence due to DCF was in the following order: **1** without pretreatment of the cells with the two inhibitors > **1** with pretreatment of the cells with either one of the two inhibitors > **1** with pretreatment of the cells with both inhibitors ≈ **14**. In fact, the intracellular fluorescence could hardly be observed for the last two treatment groups, showing that the two conjugates remained largely quenched under these conditions. This trend, which reflects the extent of recovery of ROS generation, was in accord with that of the fluorescence recovery inside the cells (Figure 5c,d). For the MMP-2-negative HeLa cells, the intracellular fluorescence remained very weak even for **1** without pretreatment of the cells with the two inhibitors because of the intrinsically low MMP levels in the cells.

The dark- and photocytotoxicity of **1** was also investigated against all four cell lines under the different conditions as described, using **14** as a negative control. Figure 6b shows the corresponding dose-dependent survival curves. Without light irradiation, both **1** and **14** were noncytotoxic up to 4 μM for all conditions. Upon light irradiation (λ > 610 nm, 23 mW cm^–2^, 28 J cm^–2^), both conjugates however could induce a different extent of cytotoxicity. The corresponding half-maximal inhibitory concentrations (IC_50_ values) were determined and are summarized in Table 2. PMB **1** exhibited the highest photocytotoxicity, particularly toward the two MMP-2-positive cell lines. The IC_50_ values were 0.78 and 0.91 μM for A549 and U-87 MG cells, respectively, which were significantly lower than those for HeLa (3.06 μM) and HEK-293 (>4 μM) cells. The results could be attributed to the intrinsic difference in the MMP-2 levels in these cells. Upon pretreatment with either SB-3CT or CA-074 Me, the photocytotoxicity of **1** was significantly reduced with IC_50_ values in the range of 2.07–2.72 μM for the two MMP-2-positive cell lines due to the partial inhibition, while the values for the two MMP-2-negative cell lines were >4 μM. The photocytotoxicity was further reduced under the dual inhibition condition, for which the IC_50_ values (>4 μM) also could not be determined. Similarly, **14** also exhibited low photocytotoxicity due to its non-cleavable and non-activatable nature. The overall results generally followed the trend of intracellular ROS generation as reported above.

**Table 2:** IC_50_ Values for **1** and **14** against A549, U-87 MG, HeLa, and HEK-293 Cells under Different Conditions with Light Irradiation (λ > 610 nm, 23 mW cm^–2^, 28 J cm^–2^)

	IC_50_ (μM)			
	MMP-2-positive		MMP-2-negative	
conditions	A549	U-87 MG	HeLa	HEK-293
**1**	0.78 ± 0.04	0.91 ± 0.03	3.06 ± 0.02	>4
**1** + CA-074 Me	2.39 ± 0.02	2.72 ± 0.01	>4	>4
**1** + SB-3CT	2.07 ± 0.01	2.55 ± 0.04	>4	>4
**1** + SB-3CT + CA-074 Me	>4	>4	>4	>4
**14**	>4	>4	>4	>4

### In Vivo Activation and PDT

**Figure 7:**
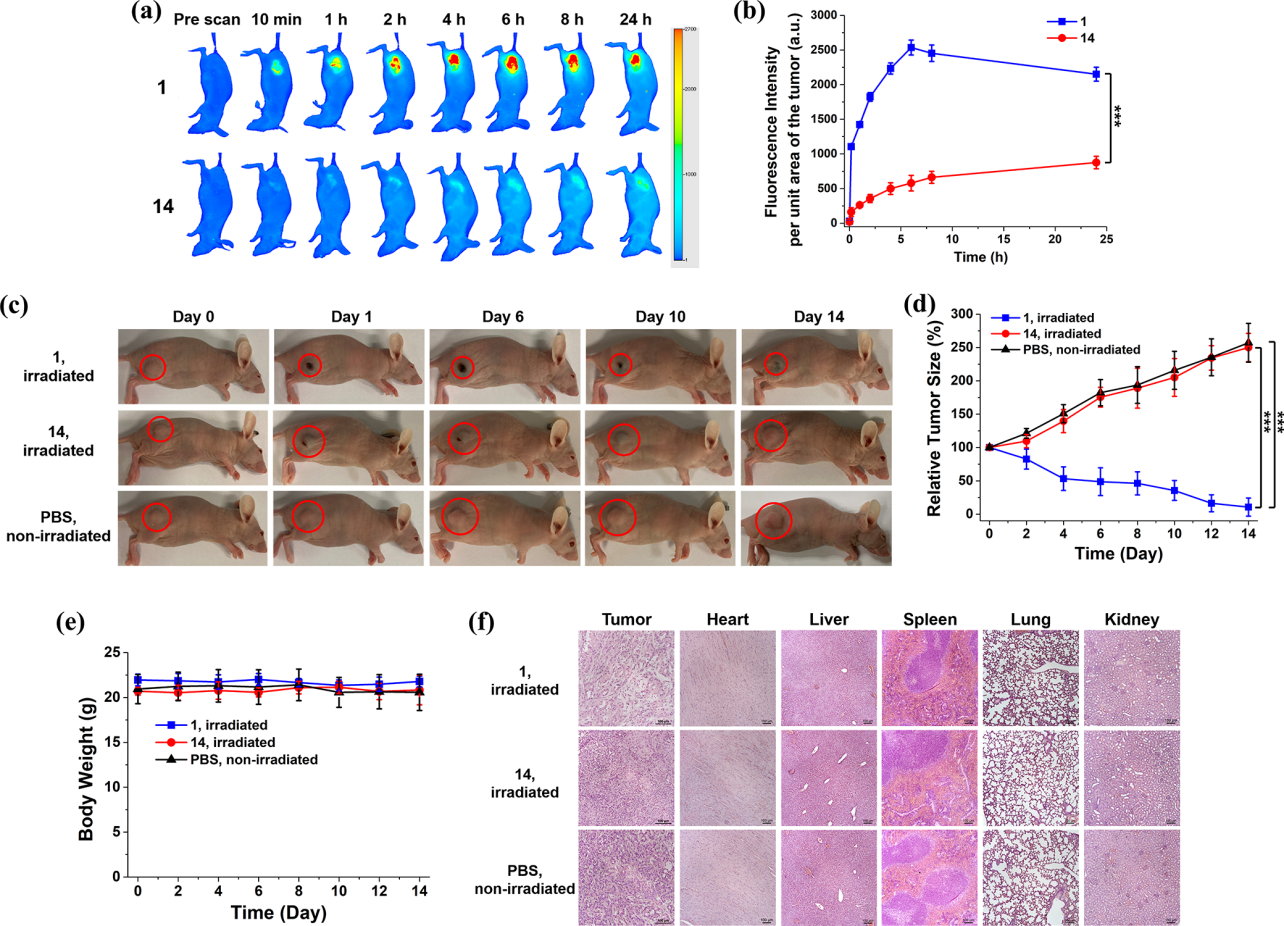
(a) Fluorescence images of A549 tumor-bearing nude mice before and after intratumoral injection of **1** or **14** over a period of 24 h (excitation wavelength = 680 nm, emission wavelength ≥700 nm). (b) Change in fluorescence intensity per unit area of the tumor for the mice being treated with **1** or **14** over 24 h. (c) Photographs of the A549 tumor-bearing nude mice before and after intratumoral injection with **1** or **14** followed by laser irradiation (680 nm, 0.3 W cm^–2^, 180 J cm^–2^) or with PBS without laser irradiation over 14 days. (d) Tumor-growth curves for A549 tumor-bearing nude mice after the aforementioned treatments. (e) Change in body weight of the mice being treated as above over 14 days. (f) H&E-stained slices of the tumor and major organs of the mice on Day 14 after the above treatments. The drug dose was 20 nmol in 20 μL of distilled water containing 7.5% DMSO and 0.5% Tween 80 (v/v) for all cases. For (b,d,e), data are expressed as the mean ± standard deviation for *n* = 4. ***p < 0.001 as calculated by the Student’s *t*-test.

The in vivo activation of **1** in terms of fluorescence emission was further investigated using A549 tumor-bearing nude mice as the animal model and the non-cleavable analogue **14** as the negative control. These conjugates were injected intratumorally into the mice, and then their whole-body fluorescence was monitored with an infrared imaging system (excitation wavelength = 680 nm, emission wavelength ≥700 nm) over a period of 24 h (Figure 7a). The fluorescence intensity per unit area of the tumor was also determined at different time points for both conjugates (Figure 7b). It can be seen that for PMB **1**, the fluorescence was gradually increased at the tumor site during the first 6 h and then dropped slightly. For **14**, the tumoral fluorescence intensity was significantly lower for the whole period of time. At 6 h post-injection, the intensity of **1** was 4.3-fold higher than that of **14**, showing that the former could be activated through enzymatic cleavage of the two peptide segments by the intrinsic MMP-2 and cathepsin B in the tumor, while the latter remained largely quenched due to its non-cleavable nature.

To further examine the in vivo PDT efficacy of **1**, A549 tumor-bearing nude mice were first intratumorally injected with **1** or the non-cleavable control **14** (20 nmol) in 20 μL of distilled water containing 7.5% dimethyl sulfoxide (DMSO) and 0.5% Tween 80 (v/v). After 6 h, when the difference in tumoral fluorescence intensity was the largest between the two conjugates (Figure 7b), the tumor was irradiated with a diode laser at 680 nm (0.3 W cm^–2^) for 10 min. As another negative control, another group of mice were injected with PBS only without the laser treatment. The tumor size of all mice was monitored continuously for 14 days after the different treatments. As shown in Figure 7c,d, while the tumor grew continuously for both control groups (i.e., **14** with laser irradiation and PBS only), the tumor size was greatly reduced for the positive treatment group (i.e., **1** with laser irradiation). Although residual fluorescence could be observed in the tumor when **14** was injected (Figure 7b), the negligible antitumor effect suggested that the amount of ROS generated by this compound, if any, was not sufficient to cause a significant photodynamic effect, which could be attributed to its non-cleavable nature and the strong intrinsic quenching.

The body weight of the mice was also monitored during the course of these treatments (Figure 7e). The negligible change suggested that these treatments did not cause a notable side effect to the mice. In addition, the PDT effect on the tumor and major organs was further investigated by hematoxylin and eosin (H&E) staining. As shown in Figure 7f, only the H&E-stained image of the tumor after the treatment with **1** and laser irradiation indicated significant cellular damage and necrosis. There was no apparent damage to the tumor tissue for the other two treatments and to the major organs for all three treatments. All these in vivo results showed that **1** could serve as a potent and safe PDT agent at the animal level.

**Figure 8:**
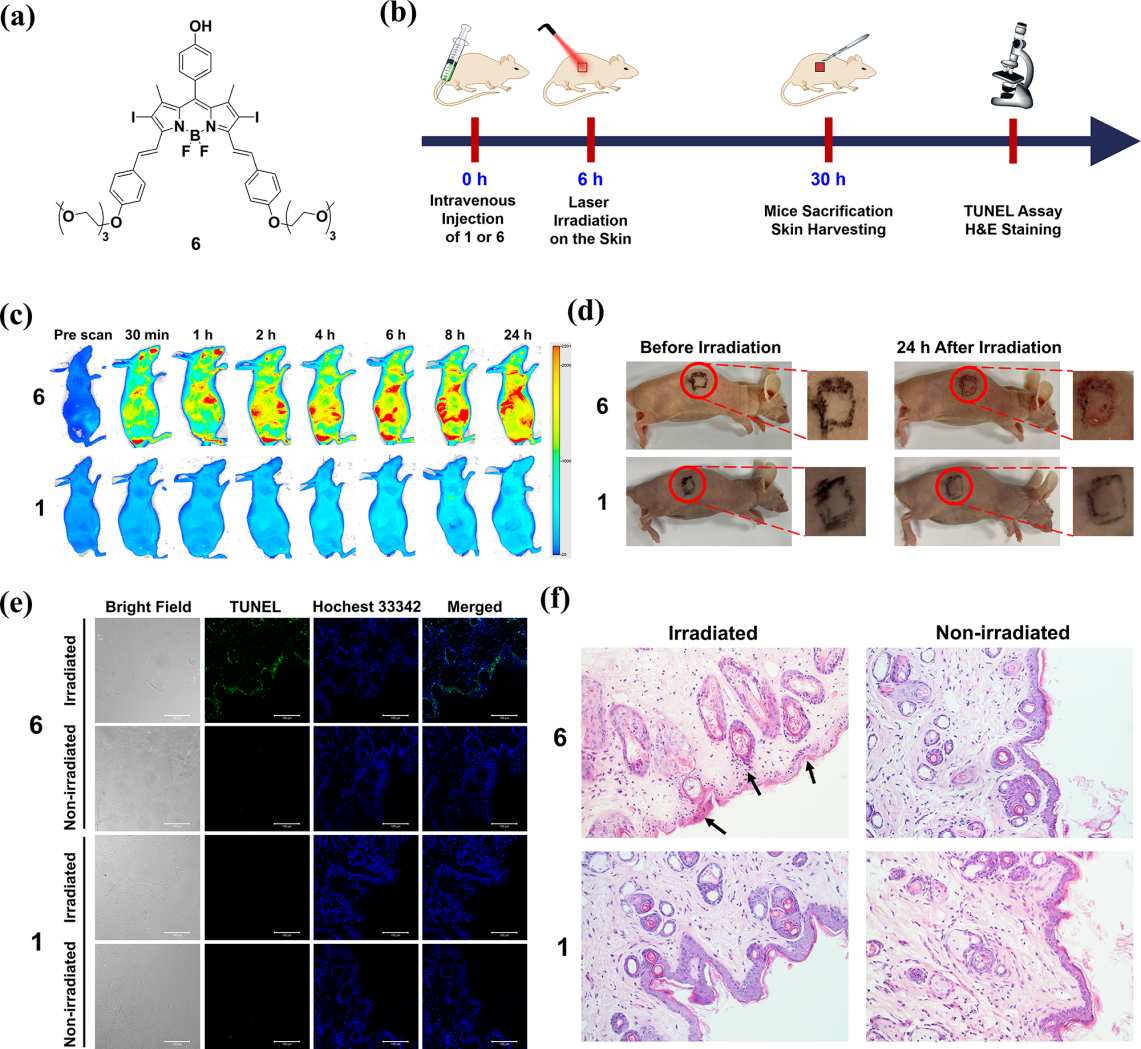
(a) Molecular structure of **6**. (b) Timeline for the investigation of the in vivo photodynamic effect of **1** and **6** on the skin of the mice. (c) Fluorescence images of the nude mice before and after intravenous injection of **1** or **6** [20 nmol in 200 μL of distilled water containing 3% DMSO (v/v) and 0.5% Tween 80 (v/v)] over a period of 24 h (excitation wavelength = 680 nm, emission wavelength ≥700 nm). (d) Photographs of the nude mice before and after laser irradiation (680 nm, 0.3 W cm^–2^) for 10 min at 6 h post-injection taken after 24 h. (e) TUNEL-stained slices of the skins of the mice at 24 h after the above treatments. The cell nuclei were stained with Hoechst 33342. (f) H&E-stained slices of the skins of the mice at 24 h after the above treatments (original magnification 200×).

As mentioned earlier, prolonged skin photosensitivity is a very common side effect of PDT. It was expected that PMB **1**, of which the photodynamic activity can be controlled precisely, could exhibit minimal toxicities at the nontarget sites, including the skin tissue. To demonstrate this advantageous feature of this smart photosensitizer, we examined the effects of **1** and laser irradiation on the skin of mice and compared the results with those for DSBDP **6** (Figure 8a), which is an “always-on” counterpart. The timeline of the study is shown in Figure 8b. The mice were first intravenously injected with either **1** or **6** (20 nmol) in 200 μL of distilled water containing 3% DMSO and 0.5% Tween 80 (v/v). The whole-body fluorescence images of the mice were monitored using an infrared imaging system over a period of 24 h. As shown in Figure 8c, bright fluorescence was observed throughout the whole body of the mice being treated with **6** over this period of time, reflecting its “always-on” nature. In contrast, fluorescence was not noticeable for the mice being treated with **1**, showing that **1** remained largely quenched inside the mice.

At 6 h post-injection of **1** or **6**, the mice were irradiated with the aforementioned laser source (680 nm, 0.3 W cm^–2^) on a region of the skin (marked in Figure 8d) for 10 min, which were the conditions used for in vivo PDT as described above. The appearance of the irradiated region was then examined after 24 h. A clear burning sign was observed for the mice being treated with **6**, while there was no noticeable change for those being treated with **1**, showing that the burning sign was caused by the photodynamic action of **6** instead of the laser irradiation. The skin tissues of these mice were then harvested and examined using the TUNEL assay. As shown in Figure 8e, bright green fluorescence was observed only for the tissue from the mice being treated with **6** followed by laser irradiation, which indicated the occurrence of extensive cell apoptosis. The fluorescence intensity was negligible without laser irradiation and for the mice being treated with **1** regardless of whether laser irradiation was applied. The results indicated that light excitation of the “always-on” photosensitizer **6** had significantly damaged the skin tissue of the mice, while for the double-locked PMB **1**, it did not cause significant cellular damage to the skin of the mice even upon light irradiation.

Further evidence was obtained by the H&E staining of these skin tissues. As shown in Figure 8f, for the tissue of the mice being treated with **6** and laser irradiation, it displayed many apoptotic keratinocytes (as indicated by black arrows) per field. They are smaller than the healthy keratinocytes and are characterized by their dark and condensed nucleic chromatin. Again, without the subsequent laser treatment and for the tissues of the mice being treated with **1** with or without laser irradiation, the skin tissues remined virtually intact. All of the above in vivo results demonstrated that the photodynamic action of the double-locked PMB **1** could be confined at the tumor without the problem of photosensitivity.

## Conclusions

In summary, a novel double-locked PMB (compound **1**) has been designed and synthesized, in which a DSBDP-based photosensitizer and a BHQ-3 quencher are connected via two enzyme-cleavable peptide segments of a cyclic peptide. The synthesis involves a tailor-made linear peptide precursor with two terminal cysteine residues and an azide group in the middle, which can facilitate cyclization of the peptide and conjugation with the photosensitizer and quencher via a one-pot two-step process. The highly efficient FRET between the two components largely inhibits the fluorescence emission and ROS generation of the conjugate, making it virtually completely quenched in the native state. Upon interactions with MMP-2 and cathepsin B, the specific enzymes for the two peptide linkers, the PMB can be activated remarkably through cleavage of the two linkers and release of the free DSBDP unit. In the presence of only one of these enzymes, the two components are still connected with a peptide segment and the PMB remains significantly quenched. These enzyme-responsive properties have been demonstrated through a series of solution, in vitro, and in vivo studies. The overall results show that PMB **1** functions as an AND logic gate that can only be unlocked by the two enzymes. The specific activation of this PMB by the coexistence of these two tumor-associated enzymes and the negligible photosensitivity render it a highly potent and promising photosensitizer for targeted PDT.

## Supporting Information Available

Experimental details; synthetic routes of different precursors; course of the formation of **14** as monitored by LC–MS; additional electronic absorption and fluorescence spectra; singlet oxygen generation efficiency; enzyme-responsive spectral data; confocal microscopic and flow cytometric data for **1** and **14** under different conditions against HeLa and HEK-293 cells; subcellular localization of **1** in A549 cells; and HPLC chromatograms, NMR spectra, and/or ESI mass spectra of all new compounds (PDF)

## References

[ref1] Brown S. B.; Brown E. A.; Walker I. The present and future role of photodynamic therapy in cancer treatment. Lancet Oncol. 2004, 5, 497–508. 10.1016/s1470-2045(04)01529-3. 15288239

[ref2] Correia J. H.; Rodrigues J. A.; Pimenta S.; Dong T.; Yang Z. Photodynamic therapy review: principles, photosensitizers, applications, and future directions. Pharmaceutics 2021, 13, 1332 10.3390/pharmaceutics13091332. 34575408 PMC8470722

[ref3] Algorri J. F.; Ochoa M.; Roldán-Varona P.; Rodríguez-Cobo L.; López-Higuera J. M. Photodynamic therapy: a compendium of latest reviews. Cancers 2021, 13, 4447 10.3390/cancers13174447. 34503255 PMC8430498

[ref4] Mishchenko T.; Balalaeva I.; Gorokhova A.; Vedunova M.; Krysko D. V. Which cell death modality wins the contest for photodynamic therapy of cancer?. Cell Death Dis. 2022, 13, 455 10.1038/s41419-022-04851-4. 35562364 PMC9106666

[ref5] Castano A. P.; Mroz P.; Hamblin M. R. Photodynamic therapy and anti-tumour immunity. Nat. Rev. Cancer 2006, 6, 535–545. 10.1038/nrc1894. 16794636 PMC2933780

[ref6] Dougherty T. J.; Cooper M. T.; Mang T. S. Cutaneous phototoxic occurrences in patients receiving Photofrin. Lasers Surg. Med. 1990, 10, 485–488. 10.1002/lsm.1900100514. 2146455

[ref7] Highlights of prescribing information: Photofrin (porfimer sodium) injection. https://www.accessdata.fda.gov/drugsatfda_docs/label/2011/020451s020lbl.pdf (accessed December 24, 2022).

[ref8] Gomes-da-Silva L. C.; Kepp O.; Kroemer G. Regulatory approval of photoimmunotherapy: photodynamic therapy that induces immunogenic cell death. Oncoimmunology 2020, 9, e1841393 10.1080/2162402X.2020.1841393. PMC7595598 33178498

[ref9] Wan Y.; Fu L.-H.; Li C.; Lin J.; Huang P. Conquering the hypoxia limitation for photodynamic therapy. Adv. Mater. 2021, 33, 2103978 10.1002/adma.202103978. 34580926

[ref10] Zheng X.; Sun W.; Ju M.; Wu J.; Huang H.; Shen B. A chemical biology toolbox to overcome the hypoxic tumor microenvironment for photodynamic therapy: a review. Biomater. Sci. 2022, 10, 4681–4693. 10.1039/d2bm00776b. 35822831

[ref11] Hong L.; Li J.; Luo Y.; Guo T.; Zhang C.; Ou S.; Long Y.; Hu Z. Recent advances in strategies for addressing hypoxia in tumor photodynamic therapy. Biomolecules 2022, 12, 81 10.3390/biom12010081. 35053229 PMC8774200

[ref12] Algorri J. F.; Ochoa M.; Roldán-Varona P.; Rodríguez-Cobo L.; López-Higuera J. M. Light technology for efficient and effective photodynamic therapy: a critical review. Cancers 2021, 13, 3484 10.3390/cancers13143484. 34298707 PMC8307713

[ref13] Li W.-P.; Yen C.-J.; Wu B.-S.; Wong T.-W. Recent advances in photodynamic therapy for deep-seated tumors with the aid of nanomedicine. Biomedicines 2021, 9, 69 10.3390/biomedicines9010069. 33445690 PMC7828119

[ref14] Sun B.; Rahmat J. N. B.; Zhang Y. Advanced techniques for performing photodynamic therapy in deep-seated tissues. Biomaterials 2022, 291, 121875 10.1016/j.biomaterials.2022.121875. 36335717

[ref15] Lan M.; Zhao S.; Liu W.; Lee C.-S.; Zhang W.; Wang P. Photosensitizers for photodynamic therapy. Adv. Healthcare Mater. 2019, 8, 1900132 10.1002/adhm.201900132. 31067008

[ref16] Zhao X.; Liu J.; Fan J.; Chao H.; Peng X. Recent progress in photosensitizers for overcoming the challenges of photodynamic therapy: from molecular design to application. Chem. Soc. Rev. 2021, 50, 4185–4219. 10.1039/d0cs00173b. 33527104

[ref17] Pham T. C.; Nguyen V.-N.; Choi Y.; Lee S.; Yoon J. Recent strategies to develop innovative photosensitizers for enhanced photodynamic therapy. Chem. Rev. 2021, 121, 13454–13619. 10.1021/acs.chemrev.1c00381. 34582186

[ref18] Hu T.; Wang Z.; Shen W.; Liang R.; Yan D.; Wei M. Recent advances in innovative strategies for enhanced cancer photodynamic therapy. Theranostics 2021, 11, 3278–3300. 10.7150/thno.54227. 33537087 PMC7847668

[ref19] Lee D.; Kwon S.; Jang S.-y.; Park E.; Lee Y.; Koo H. Overcoming the obstacles of current photodynamic therapy in tumors using nanoparticles. Bioact. Mater. 2022, 8, 20–34. 10.1016/j.bioactmat.2021.06.019. 34541384 PMC8424083

[ref20] Li S.; Yang F.; Wang Y.; Du T.; Hou X. Emerging nanotherapeutics for facilitating photodynamic therapy. Chem. Eng. J. 2023, 451, 138621 10.1016/j.cej.2022.138621.

[ref21] Li M.; Xu Y.; Peng X.; Kim J. S. From low to no O_2_-dependent hypoxia photodynamic therapy (hPDT): a new perspective. Acc. Chem. Res. 2022, 55, 3253–3264. 10.1021/acs.accounts.2c00531. 36323625

[ref22] Li D.; Liu P.; Tan Y.; Zhang Z.; Kang M.; Wang D.; Tang B. Z. Type I photosensitizers based on aggregation-induced emission: a rising star in photodynamic therapy. Biosensors 2022, 12, 722 10.3390/bios12090722. 36140107 PMC9496375

[ref23] Cheng X.; Gao J.; Ding Y.; Lu Y.; Wei Q.; Cui D.; Fan J.; Li X.; Zhu E.; Lu Y.; Wu Q.; Li L.; Huang W. Multi-Functional Liposome: A Powerful Theranostic Nano-Platform Enhancing Photodynamic Therapy. Adv. Sci. 2021, 8, 2100876 10.1002/advs.202100876. PMC8373168 34085415

[ref24] Li Y.; Zhang M.; Han H.; Zhang B.; Matson J. B.; Chen D.; Li W.; Wang Y. Peptide-based supramolecular photodynamic therapy systems: from rational molecular design to effective cancer treatment. Chem. Eng. J. 2022, 436, 135240 10.1016/j.cej.2022.135240.

[ref25] Sandland J.; Boyle R. W. Photosensitizer antibody-drug conjugates: past, present, and future. Bioconjugate Chem. 2019, 30, 975–993. 10.1021/acs.bioconjchem.9b00055. 30768894

[ref26] Gierlich P.; Mata A. I.; Donohoe C.; Brito R. M. M.; Senge M. O.; Gomes-da-Silva L. C. Ligand-targeted delivery of photosensitizers for cancer treatment. Molecules 2020, 25, 5317 10.3390/molecules25225317. 33202648 PMC7698280

[ref27] Wang X.; Luo D.; Basilion J. P. Photodynamic therapy: targeting cancer biomarkers for the treatment of cancers. Cancers 2021, 13, 2992 10.3390/cancers13122992. 34203805 PMC8232794

[ref28] Lim C. K.; Heo J.; Shin S.; Jeong K.; Seo Y. H.; Jang W. D.; Park C. R.; Park S. Y.; Kim S.; Kwon I. C. Nanophotosensitizers toward advanced photodynamic therapy of cancer. Cancer Lett. 2013, 334, 176–187. 10.1016/j.canlet.2012.09.012. 23017942

[ref29] Lucky S. S.; Soo K. C.; Zhang Y. Nanoparticles in photodynamic therapy. Chem. Rev. 2015, 115, 1990–2042. 10.1021/cr5004198. 25602130

[ref30] Li X.; Kolemen S.; Yoon J.; Akkaya E. U. Activatable photosensitizers: agents for selective photodynamic therapy. Adv. Funct. Mater. 2017, 27, 1604053 10.1002/adfm.201604053.

[ref31] Luby B. M.; Walsh C. D.; Zheng G. Advanced photosensitizer activation strategies for smarter photodynamic therapy beacons. Angew. Chem., Int. Ed. 2019, 58, 2558–2569. 10.1002/anie.201805246. 29890024

[ref32] Cheng P.; Pu K. Activatable Phototheranostic Materials for Imaging-Guided Cancer Therapy. ACS Appl. Mater. Interfaces 2020, 12, 5286–5299. 10.1021/acsami.9b15064. 31730329

[ref33] Liu M.; Li C. Recent advances in activatable organic photosensitizers for specific photodynamic therapy. ChemPlusChem 2020, 85, 948–957. 10.1002/cplu.202000203. 32401421

[ref34] Yang M.; Li X.; Yoon J. Activatable supramolecular photosensitizers: advanced design strategies. Mater. Chem. Front. 2021, 5, 1683–1693. 10.1039/d0qm00827c.

[ref35] Hou W.; Xia F.; Alves C. S.; Qian X.; Yang Y.; Cui D. MMP2-targeting and redox-responsive PEGylated chlorin e6 nanoparticles for cancer near-infrared imaging and photodynamic therapy. ACS Appl. Mater. Interfaces 2016, 8, 1447–1457. 10.1021/acsami.5b10772. 26638778

[ref36] Chen Q.; Feng L.; Liu J.; Zhu W.; Dong Z.; Wu Y.; Liu Z. Intelligent albumin–MnO_2_ nanoparticles as pH-/H_2_O_2_-responsive dissociable nanocarriers to modulate tumor hypoxia for effective combination therapy. Adv. Mater. 2016, 28, 7129–7136. 10.1002/adma.201601902. 27283434

[ref37] Dai X.; Han K.; Ma Z.; Han H. A chimeric peptide logic gate for orthogonal stimuli-triggered precise tumor therapy. Adv. Funct. Mater. 2018, 28, 1804609 10.1002/adfm.201804609.

[ref38] Zhao H.; Li L.; Zheng C.; Hao Y.; Niu M.; Hu Y.; Chang J.; Zhang Z.; Wang L. An intelligent dual stimuli-responsive photosensitizer delivery system with O_2_-supplying for efficient photodynamic therapy. Colloids Surf., B 2018, 167, 299–309. 10.1016/j.colsurfb.2018.04.011. 29679806

[ref39] Jing X.; Zhi Z.; Jin L.; Wang F.; Wu Y.; Wang D.; Yan K.; Shao Y.; Meng L. pH/redox dual-stimuli-responsive cross-linked polyphosphazene nanoparticles for multimodal imaging-guided chemo-photodynamic therapy. Nanoscale 2019, 11, 9457–9467. 10.1039/c9nr01194c. 31042245

[ref40] Teng K. X.; Niu L. Y.; Kang Y. F.; Yang Q. Z. Rational design of a “dual lock-and-key” supramolecular photosensitizer based on aromatic nucleophilic substitution for specific and enhanced photodynamic therapy. Chem. Sci. 2020, 11, 9703–9711. 10.1039/d0sc01122c. 34094236 PMC8162035

[ref41] Fu Y.; Jang M. S.; Wang N.; Li Y.; Wu T. P.; Lee J. H.; Lee D. S.; Yang H. Y. Dual activatable self-assembled nanotheranostics for bioimaging and photodynamic therapy. J. Controlled Release 2020, 327, 129–139. 10.1016/j.jconrel.2020.07.045. 32771476

[ref42] Zhang P.; Gao Z.; Cui J.; Hao J. Dual-stimuli-responsive polypeptide nanoparticles for photothermal and photodynamic therapy. ACS Appl. Bio Mater. 2020, 3, 561–569. 10.1021/acsabm.9b00964. 35019399

[ref43] Cheng D.; Ji Y.; Wang B.; Jin T.; Xu Y.; Qian X.; Zhu W. Enzyme/GSH dual-responsive biodegradable nanohybrid for spatiotemporally specific photodynamic and hypoxia-augmented therapy against tumors. Int. J. Pharm. 2021, 603, 120730 10.1016/j.ijpharm.2021.120730. 34029662

[ref44] Lau J. T. F.; Lo P.-C.; Jiang X.-J.; Wang Q.; Ng D. K. P. A dual activatable photosensitizer toward targeted photodynamic therapy. J. Med. Chem. 2014, 57, 4088–4097. 10.1021/jm500456e. 24793456

[ref45] Jiang X.-J.; Lau J. T. F.; Wang Q.; Ng D. K. P.; Lo P.-C. pH- and thiol-responsive BODIPY-based photosensitizers for targeted photodynamic therapy. Chem.–Eur. J. 2016, 22, 8273–8281. 10.1002/chem.201600452. 27139139

[ref46] Sun J.; Du K.; Diao J.; Cai X.; Feng F.; Wang S. GSH and H_2_O_2_ co-activatable mitochondria-targeted photodynamic therapy under normoxia and hypoxia. Angew. Chem., Int. Ed. 2020, 59, 12122–12128. 10.1002/anie.202003895. 32297412

[ref47] Turkoglu G.; Koygun G. K.; Yurt M. N. Z.; Pirencioglu S. N.; Erbas-Cakmak S. A therapeutic keypad lock decoded in drug resistant cancer cells. Chem. Sci. 2021, 12, 9754–9758. 10.1039/d1sc02521j. 34349948 PMC8293978

[ref48] Tam L. K. B.; Yu L.; Wong R. C. H.; Fong W.-P.; Ng D. K. P.; Lo P.-C. Dual cathepsin B and glutathione-activated dimeric and trimeric phthalocyanine-based photodynamic molecular beacons for targeted photodynamic therapy. J. Med. Chem. 2021, 64, 17455–17467. 10.1021/acs.jmedchem.1c01634. 34846143

[ref49] Tam L. K. B.; He L.; Ng D. K. P.; Cheung P. C. K.; Lo P.-C. A tumor-targeting dual-stimuli-activatable photodynamic molecular beacon for precise photodynamic therapy. Chem.–Eur. J. 2022, 28, e202201652 10.1002/chem.202201652. 35852020

[ref50] Ozlem S.; Akkaya E. U. Thinking outside the silicon box: molecular AND logic as an additional layer of selectivity in singlet oxygen generation for photodynamic therapy. J. Am. Chem. Soc. 2009, 131, 48–49. 10.1021/ja808389t. 19086786

[ref51] Zhang N.; Zhao F.; Zou Q.; Li Y.; Ma G.; Yan X. Multitriggered tumor-responsive drug delivery vehicles based on protein and polypeptide coassembly for enhanced photodynamic tumor ablation. Small 2016, 12, 5936–5943. 10.1002/smll.201602339. 27622681

[ref52] Chen Y.; Li X.; Zhao Y.; Zhang X.; Sun L. Preparation of Triple-Responsive Porous Silica Carriers and Carbon Quantum Dots for Photodynamic-/Chemotherapy and Multicolor Cell Imaging. ChemNanoMat 2020, 6, 648–656. 10.1002/cnma.201900777.

[ref53] Prost M.; Hasserodt J. “Double gating”—a concept for enzyme-responsive imaging probes aiming at high tissue specificity. Chem. Commun. 2014, 50, 14896–14899. 10.1039/c4cc07147f. 25325798

[ref54] Li S.-Y.; Liu L.-H.; Cheng H.; Li B.; Qiu W.-X.; Zhang X.-Z. A dual-FRET-based fluorescence probe for the sequential detection of MMP-2 and caspase-3. Chem. Commun. 2015, 51, 14520–14523. 10.1039/c5cc04962h. 26283215

[ref55] Cheng H.; Li S.-Y.; Zheng H.-R.; Li C.-X.; Xie B.-R.; Chen K.-W.; Li B.; Zhang X.-Z. Multi-Förster Resonance Energy Transfer-Based Fluorescent Probe for Spatiotemporal Matrix Metalloproteinase-2 and Caspase-3 Imaging. Anal. Chem. 2017, 89, 4349–4354. 10.1021/acs.analchem.7b00277. 28365980

[ref56] Liu Y.; Teng L.; Xu C.; Liu H.-W.; Xu S.; Guo H.; Yuan L.; Zhang X.-B. A “double-locked” and enzyme-activated molecular probe for accurate bioimaging and hepatopathy differentiation. Chem. Sci. 2019, 10, 10931–10936. 10.1039/c9sc03628h. 32190249 PMC7066674

[ref57] Henriet P.; Emonard H. Matrix metalloproteinase-2: not (just) a “hero” of the past. Biochimie 2019, 166, 223–232. 10.1016/j.biochi.2019.07.019. 31362036

[ref58] Gondi C. S.; Rao J. S. Cathepsin B as a cancer target. Expert Opin. Ther. Targets 2013, 17, 281–291. 10.1517/14728222.2013.740461. 23293836 PMC3587140

[ref59] Han K.; Lei Q.; Jia H.-Z.; Wang S.-B.; Yin W.-N.; Chen W.-H.; Cheng S.-X.; Zhang X.-Z. A tumor targeted chimeric peptide for synergistic endosomal escape and therapy by dual-stage light manipulation. Adv. Funct. Mater. 2015, 25, 1248–1257. 10.1002/adfm.201403190.

[ref60] Cheng Y.-J.; Qin S.-Y.; Liu W.-L.; Ma Y.-H.; Chen X.-S.; Zhang A.-Q.; Zhang X.-Z. Dual-Targeting Photosensitizer-Peptide Amphiphile Conjugate for Enzyme-Triggered Drug Delivery and Synergistic Chemo-Photodynamic Tumor Therapy. Adv. Mater. Interfaces 2020, 7, 2000935 10.1002/admi.202000935.

[ref61] Ke M.-R.; Ng D. K. P.; Lo P.-C. Synthesis and in vitro photodynamic activities of an integrin-targeting cRGD-conjugated zinc(II) phthalocyanine. Chem.–Asian J. 2014, 9, 554–561. 10.1002/asia.201301166. 24203795

[ref62] Kue C. S.; Ng S. Y.; Voon S. H.; Kamkaew A.; Chung L. Y.; Kiew L. V.; Lee H. B. Recent strategies to improve boron dipyrromethene (BODIPY) for photodynamic cancer therapy: an updated review. Photochem. Photobiol. Sci. 2018, 17, 1691–1708. 10.1039/c8pp00113h. 29845993

[ref63] Chu J. C. H.; Shao C.; Ha S. Y. Y.; Fong W.-P.; Wong C. T. T.; Ng D. K. P. One-pot peptide cyclisation and surface modification of photosensitiser-loaded red blood cells for targeted photodynamic therapy. Biomater. Sci. 2021, 9, 7832–7837. 10.1039/d1bm01306h. 34726672

[ref64] Chu J. C. H.; Yang C.; Fong W.-P.; Wong C. T. T.; Ng D. K. P. Facile one-pot synthesis of cyclic peptide-conjugated photosensitisers for targeted photodynamic therapy. Chem. Commun. 2020, 56, 11941–11944. 10.1039/d0cc05264g. 32931540

[ref65] Dommerholt J.; Rutjes F. P. J. T.; van Delft F. L. Strain-promoted 1,3-dipolar cycloaddition of cycloalkynes and organic azides. Top. Curr. Chem. 2016, 374, 16 10.1007/s41061-016-0016-4. PMC5480410 27573141

[ref66] Dommerholt J.; Schmidt S.; Temming R.; Hendriks L. J. A.; Rutjes F. P. J. T.; van Hest J. C. M.; Lefeber D. J.; Friedl P.; van Delft F. L. Readily accessible bicyclononynes for bioorthogonal labeling and three-dimensional imaging of living cells. Angew. Chem., Int. Ed. 2010, 49, 9422–9425. 10.1002/anie.201003761. PMC3021724 20857472

[ref67] Entradas T.; Waldron S.; Volk M. The detection sensitivity of commonly used singlet oxygen probes in aqueous environments. J. Photochem. Photobiol., B 2020, 204, 111787 10.1016/j.jphotobiol.2020.111787. 31958676

[ref68] Sheen-Chen S.-M.; Chen H.-S.; Eng H.-L.; Sheen C.-C.; Chen W.-J. Serum levels of matrix metalloproteinase 2 in patients with breast cancer. Cancer Lett. 2001, 173, 79–82. 10.1016/s0304-3835(01)00657-7. 11578812

[ref69] Yoon M. C.; Christy M. P.; Phan V. V.; Gerwick W. H.; Hook G.; O’Donoghue A. J.; Hook V. Molecular features of CA-074 pH-dependent inhibition of cathepsin B. Biochemistry 2022, 61, 228–238. 10.1021/acs.biochem.1c00684. 35119840 PMC9096814

[ref70] Sindbert S.; Kalinin S.; Nguyen H.; Kienzler A.; Clima L.; Bannwarth W.; Appel B.; Müller S.; Seidel C. A. M. Accurate Distance Determination of Nucleic Acids via Förster Resonance Energy Transfer: Implications of Dye Linker Length and Rigidity. J. Am. Chem. Soc. 2011, 133, 2463–2480. 10.1021/ja105725e. 21291253

[ref71] Seltzer J. L.; Akers K. T.; Weingarten H.; Grant G. A.; McCourt D. W.; Eisen A. Z. Cleavage specificity of human skin type IV collagenase (gelatinase). Identification of cleavage sites in type I gelatin, with confirmation using synthetic peptides. J. Biol. Chem. 1990, 265, 20409–20413. 10.1016/s0021-9258(17)30519-7. 2173706

[ref72] Rejmanová P.; Kopeček J.; Pohl J.; Baudyš M.; Kostka V. Degradation of oligopeptide sequences in *N*-(2-hydroxypropyl)methacrylamide copolymers by bovine spleen cathepsin B. Makromol. Chem. 1983, 184, 2009–2020. 10.1002/macp.1983.021841006.

[ref73] Shu M.; Tang J.; Chen L.; Zeng Q.; Li C.; Xiao S.; Jiang Z.; Liu J. Tumor microenvironment triple-responsive nanoparticles enable enhanced tumor penetration and synergetic chemo-photodynamic therapy. Biomaterials 2021, 268, 120574 10.1016/j.biomaterials.2020.120574. 33271451

[ref74] VanMeter T. E.; Rooprai H. K.; Kibble M. M.; Fillmore H. L.; Broaddus W. C.; Pilkington G. J. The role of matrix metalloproteinase genes in glioma invasion: co-dependent and interactive proteolysis. J. Neuro Oncol. 2001, 53, 213–235. 10.1023/a:1012280925031. 11716072

[ref75] Wang Y.; Lin T.; Zhang W.; Jiang Y.; Jin H.; He H.; Yang V. C.; Chen Y.; Huang Y. A prodrug-type, MMP-2-targeting nanoprobe for tumor detection and imaging. Theranostics 2015, 5, 787–795. 10.7150/thno.11139. 26000052 PMC4440437

[ref76] Ali M. A. M.; Chow A. K.; Kandasamy A. D.; Fan X.; West L. J.; Crawford B. D.; Simmen T.; Schulz R. Mechanisms of cytosolic targeting of matrix metalloproteinase-2. J. Cell. Physiol. 2012, 227, 3397–3404. 10.1002/jcp.24040. 22212960

[ref77] Brown S.; Bernardo M. M.; Li Z.-H.; Kotra L. P.; Tanaka Y.; Fridman R.; Mobashery S. Potent and selective mechanism-based inhibition of gelatinases. J. Am. Chem. Soc. 2000, 122, 6799–6800. 10.1021/ja001461n.

[ref78] Buttle D. J.; Murata M.; Knight C. G.; Barrett A. J. CA074 methyl ester: a proinhibitor for intracellular cathepsin B. Arch. Biochem. Biophys. 1992, 299, 377–380. 10.1016/0003-9861(92)90290-d. 1444478

[ref79] Chen X.; Zhong Z.; Xu Z.; Chen L.; Wang Y. 2′,7′-Dichlorodihydrofluorescein as a fluorescent probe for reactive oxygen species measurement: Forty years of application and controversy. Free Radical Res. 2010, 44, 587–604. 10.3109/10715761003709802. 20370560

