# Supplementary material for: Enzyme-Responsive Double-Locked Photodynamic Molecular Beacon for Targeted Photodynamic Anticancer Therapy
Source: J Am Chem Soc. 2023 Mar 24;145(13):7361–75. doi: 10.1021/jacs.2c13732 (PMC10080691; doi:10.1021/jacs.2c13732)
Supplement: Supplementary file 1 — ja2c13732_si_001.pdf [file ja2c13732_si_001.pdf]

Supporting Information

Enzyme-Responsive Double-Locked Photodynamic Molecular Beacon for Targeted Photodynamic Anticancer Therapy

Leo K. B. Tam,[†] Jacky C. H. Chu,[†] Lin He,[‡] Caixia Yang,[†] Kam-Chu Han,[§] Peter Chi Keung Cheung,[‖] Dennis K. P. Ng,*[†] and Pui-Chi Lo*[‡]

[†] *Department of Chemistry, The Chinese University of Hong Kong, Shatin, N.T., Hong Kong, China. E-mail: dkpn@cuhk.edu.hk*

[‡] *Department of Biomedical Sciences and Tung Biomedical Sciences Centre, City University of Hong Kong, Tat Chee Avenue, Kowloon, Hong Kong, China. E-mail: gigi.lo@cityu.edu.hk*

[§] *Department of Clinical Pathology, Pamela Youde Nethersole Eastern Hospital, Chai Wan, Hong Kong, China*

[‖] *School of Life Sciences, The Chinese University of Hong Kong, Shatin, N.T., Hong Kong, China*

Contents

Experimental Section

Scheme S1 Synthetic route of the azide-appended peptide **3**.

Scheme S2 Synthetic route of the azide-appended peptide **5**.

Scheme S3 Synthetic route of 3,5-bis(bromomethyl)phenyl-substituted DSBDP **8**.

Scheme S4 Synthetic route of BCN-substituted BHQ-3 **11**.

Figure S1 Monitoring the course of the formation of conjugate **14** using LC-MS.

Figure S2 (a) Electronic absorption and (b) fluorescence (λ_{ex} = 610 nm) spectra of **1**, **8**, **11**, and **14** (all at 1 μM) in DMF.

Figure S3 (a) Electronic absorption and (b) fluorescence (λ_{ex} = 610 nm) spectra of **1**, **8**, **11**, and **14** (all at 1 μM) in buffer solution (pH 5.0, 25 mM NaOAc, 1 mM EDTA, 500 μM GSH) with 0.1% Tween 80 (v/v).

Figure S4 Change in the electronic absorption spectrum of a mixture of DPBF (initial concentration = 30 μM) and (a) **1**, (b) **8**, (c) **14**, or (d) ZnPc in DMF upon irradiation (λ > 610 nm) for 30 s.

Figure S5 Change in the electronic absorption spectrum of a mixture of DPBF (initial concentration = 30 μM) and (a) **1**, (b) **8**, (c) **14**, or (d) ZnPc in PBS at pH 7.4 with 0.1% Tween 80 (v/v) upon irradiation (λ > 610 nm) for 300 s.

Figure S6 Change in the electronic absorption spectrum of a mixture of DPBF (initial concentration = 30 μM) and (a) **1**, (b) **8**, (c) **14**, or (d) ZnPc in buffer solution (pH 5.0, 25 mM NaOAc, 1 mM EDTA, 500 μM GSH) with 0.1% Tween 80 (v/v) upon irradiation (λ > 610 nm) for 300 s.

Figure S7 Comparison of the rates of decay of DPBF (initial concentration = 30 μM) sensitized by **1**, **8**, **14**, and ZnPc (all at 1 μM) in (a) DMF, (b) PBS at pH 7.4 with 0.1% Tween 80 (v/v), and (c) buffer solution (pH 5.0, 25 mM NaOAc, 1 mM EDTA, 500 μM GSH) with 0.1% Tween 80 (v/v).

Figure S8 Change in the (a,b,e,f) electronic absorption and (c,d,g,h) fluorescence (λ_{ex} = 610 nm) spectra of **1** (1 μM) in the absence or presence of MMP-2 (2 μg mL^{-1}) or cathepsin B (1 unit mL^{-1}) in PBS at pH 7.4 with 0.1% Tween 80 (v/v) (for the study involving MMP-2) or buffer solution (pH 5.0, 25 mM NaOAc, 1 mM EDTA, 500 μM GSH) with 0.1% Tween 80 (v/v) (for the study involving cathepsin B) at 37 °C over a period of 25 h.

Figure S9 Change in the (a,b,e,f) electronic absorption and (c,d,g,h) fluorescence (λ_{ex} = 610 nm) spectra of **14** (1 μM) in the absence or presence of MMP-2 (2 μg mL^{-1}) or cathepsin B (1 unit mL^{-1}) in PBS at pH 7.4 with 0.1% Tween 80 (v/v) (for the study involving MMP-2) or buffer solution (pH 5.0, 25 mM NaOAc, 1 mM

EDTA, 500 μM GSH) with 0.1% Tween 80 (v/v) (for the study involving cathepsin B) at 37 °C over a period of 25 h.

Figure S10 Change in the (a,c) electronic absorption and (b,d) fluorescence (λ_{ex} = 610 nm) spectra of (a,b) **1** and (c,d) **14** (both at 1 μM) upon treatment with MMP-2 (2 μg mL^{-1}) in PBS at pH 7.4 with 0.1% Tween 80 (v/v) at 37 °C for 6 h, and then together with cathepsin B (1 unit mL^{-1}) in buffer solution (pH 5.0, 25 mM NaOAc, 1 mM EDTA, 500 μM GSH) with 0.1% Tween 80 (v/v) at 37 °C for further 19 h.

Figure S11 Change in the electronic absorption spectrum of a mixture of DPBF (initial concentration = 30 μM) and **1** (1 μM) after being treated as follows upon light irradiation (λ > 610 nm) over a period of 300 s: in the (a) absence or (b) presence of MMP-2 (2 μg mL^{-1}) in PBS at pH 7.4 with 0.1% Tween 80 (v/v) at 37 °C for 25 h; in the (c) absence or (d) presence of cathepsin B (1 unit mL^{-1}) in buffer solution (pH 5.0, 25 mM NaOAc, 1 mM EDTA, 500 μM GSH) with 0.1% Tween 80 (v/v) at 37 °C for 25 h; (e) with MMP-2 (2 μg mL^{-1}) in PBS at pH 7.4 with 0.1% Tween 80 (v/v) at 37 °C for 6 h, and then together with cathepsin B (1 unit mL^{-1}) in buffer solution (pH 5.0, 25 mM NaOAc, 1 mM EDTA, 500 μM GSH) with 0.1% Tween 80 (v/v) at 37 °C for further 19 h.

Figure S12 Change in the electronic absorption spectrum of a mixture of DPBF (initial concentration = 30 μM) and **14** (1 μM) after being treated as follows upon light irradiation (λ > 610 nm) over a period of 300 s: in the (a) absence or (b) presence of MMP-2 (2 μg mL^{-1}) in PBS at pH 7.4 with 0.1% Tween 80 (v/v) at 37 °C for 25 h; in the (c) absence or (d) presence of cathepsin B (1 unit mL^{-1}) in buffer solution (pH 5.0, 25 mM NaOAc, 1 mM EDTA, 500 μM GSH) with 0.1% Tween 80 (v/v) at 37 °C for 25 h; (e) with MMP-2 (2 μg mL^{-1}) in PBS at pH 7.4 with 0.1% Tween 80 (v/v) at 37 °C for 6 h, and then together with cathepsin B (1 unit mL^{-1}) in buffer solution (pH 5.0, 25 mM NaOAc, 1 mM EDTA, 500 μM GSH) with 0.1% Tween 80 (v/v) at 37 °C for further 19 h.

Figure S13 Bright field, fluorescence, and the merged images of HeLa and HEK-293 cells after incubation in a serum-free medium in the absence or presence of SB-3CT (10 μM) and/or CA-074 Me (25 μM) for 2 h, and then with **1** (2 μM) for 1 h, followed by post-incubation in the medium for a further 6 h or incubation with **14** (2 μM) for 1 h, followed by post-incubation in the medium for a further 6 h. (b) The mean intracellular fluorescence intensities of HeLa and HEK-293 cells under these conditions as determined by flow cytometry.

Figure S14 Visualization of the intracellular fluorescence of the DSBDP fragment of **1** and various subcellular trackers in A549 cells. The cells were sequentially incubated

with **1** (2 μM) for 1 h, culture medium for 6 h, and then LysoTracker Green DND-26 (2 μM for 30 min), MitoTracker Green FM (0.2 μM for 15 min) or ER-Tracker Green (1 μM for 15 min).

Figure S15 HPLC chromatograms of (a) **3**, (b) **5**, (c) **11**, (d) **12**, (e) **13**, (f) **14**, and (g) **1**.

Figure S16 ^{1}H and ^{13}C{^{1}H} NMR spectra of **8** in $CDCl_3$.

Figure S18 ^{1}H and ^{13}C{^{1}H} NMR spectra of **11** in CD_3OD.

Figure S18 ESI mass spectrum of **3**.

Figure S19 ESI mass spectrum of **5**.

Figure S20 ESI mass spectrum of **8**.

Figure S21 ESI mass spectrum of **11**.

Figure S22 ESI mass spectrum of **12**.

Figure S23 ESI mass spectrum of **13**.

Figure S24 ESI mass spectrum of **1**.

Figure S25 ESI mass spectrum of **14**.

Experimental Section

General

All the reactions were performed under an atmosphere of nitrogen. CH_2Cl_2, DMF, and tetrahydrofuran (THF) were purified using an INERT solvent purification system. All other solvents and reagents were of HPLC or reagent grade and used as received. All the reactions were monitored by thin layer chromatography (TLC) performed on Merck pre-coated silica gel 60 F254 plates. Chromatographic purification was performed on silica gel (Macherey-Nagel, 230−400 mesh) with the indicated eluent and/or Bio-Rad Bio-Beads S-X1 beads (200–400 mesh) with THF as the eluent. Reverse-phase HPLC separation was performed on a XBridge BEH300 C18 column (5 µm, 4.6 mm × 150 mm) for analytical purpose or a XBridge BEH300 Prep C18 column (5 µm, 10 mm × 250 mm) for preparative purpose using a Waters system equipped with a Waters 1525 binary pump and a Waters 2998 photodiode array detector. The purity of all peptide conjugates was found to be >95% by HPLC analysis. Fmoc-Lys(N_3)-OH,[R1] DSBDP **6**,[R2] and BCN **10**[R3] were prepared as described.

^{1}H and ^{13}C{^{1}H} NMR spectra were recorded on a Bruker AVANCE III HD 500 spectrometer (^{1}H, 500 MHz; ^{13}C, 125.8 MHz) in deuterated solvents. Spectra were referenced internally using the residual solvent [^{1}H: δ = 7.26 (for $CDCl_3$), 3.31 for (CD_3OD)] or solvent [^{13}C: δ = 77.2 (for $CDCl_3$), 49.0 (for CD_3OD)] resonances relative to $SiMe_4$. ESI mass spectra were recorded on a Thermo Finnigan MAT 95 XL mass spectrometer. Electronic absorption

and steady-state fluorescence spectra were taken on a Shimazu UV-1800 UV-Vis spectrophotometer and a HORIBA FluoroMax-4 spectrofluorometer, respectively.

Preparation of 3

Peptide **3** was prepared manually using a modified Fmoc SPPS protocol as shown in Scheme S1. The Fmoc protecting group on the rink amide resin was first removed using 20% piperidine in DMF. An excess of Fmoc-protected amino acids (4 equiv) or Fmoc-Lys(N_3)-OH, HATU (4 equiv), and DIPEA (8 equiv) in DMF were used for each coupling at room temperature. After the final coupling and Fmoc deprotection, the resin was treated with a mixture of 95% TFA, 2.5% TIPS, and 2.5% water for 1 h to remove the protecting groups and release the peptide. The resin was removed by filtration, and the filtrate was precipitated upon addition of diethyl ether. After centrifugation, the supernatant was removed. The solid was redissolved in DMSO and then precipitated by the addition of diethyl ether. The crude product was lyophilized and then purified by reverse-phase HPLC. The conditions used were set as follows: solvent A = 0.1% TFA in acetonitrile and solvent B = 0.1% TFA in deionized water. Elution gradient: 0% A + 100% B in the first 5 min, changed to 100% A + 0% B in 30 min, maintained at 100% A for 5 min, changed to 0% A + 100% B in 10 min, and maintained at 100% B for further 10 min. A flow rate of 1 or 3 mL min^{-1} was used for analytical or preparative purpose, respectively. HRMS (ESI): *m/z* calcd for $C_{91}H_{164}N_{33}O_{20}S_2$ $[M+4H]^{4+}$ 522.5561, found 522.5566.

Preparation of 5

Peptide **5** was prepared according to the procedure described for **3** (Scheme S2). After lyophilization of the precipitated peptide, the crude product was purified by reverse-phase HPLC with the aforementioned conditions. HRMS (ESI): *m*/*z* calcd for $C_{66}H_{123}N_{29}O_{20}S_2$ $[M+4H]^{4+}$ 426.4729, found 426.4723.

Preparation of 8

A mixture of DSBDP **6** (30 mg, 27.5 μmol), 1,3,5 tris(bromomethyl)benzene (**7**) (29 mg, 81.3 μmol), and K_2CO_3 (8 mg, 57.9 μmol) in DMF was stirred at room temperature for 16 h. The solvent was then removed in vacuo. The residue was purified by column chromatography on silica gel with $CHCl_3$/CH_3OH (100:1 v/v) as eluent to afford **8** as a dark green solid (13 mg, 35%). ^{1}H NMR (500 MHz, $CDCl_3$) δ 8.13 (d, *J* = 16.5 Hz, 2 H, C=CH), 7.57–7.61 (m, 6 H, C=CH and Ar-H), 7.45 (s, 2 H, Ar-H), 7.42 (s, 1 H, Ar-H), 7.20 (d, *J* = 8.5 Hz, 2 H, Ar-H), 7.12 (d, *J* = 8.5 Hz, 2 H, Ar-H), 6.96 (d, *J* = 8.5 Hz, 4 H, Ar-H), 5.14 (s, 2 H, OCH_2), 4.51 (s, 4 H, CH_2Br), 4.19 (t, *J* = 4.5 Hz, 4 H, OCH_2), 3.89 (t, *J* = 4.5 Hz, 4 H, OCH_2), 3.75–3.77 (m, 4 H, OCH_2), 3.66–3.71 (m, 8 H, OCH_2), 3.55–3.57 (m, 4 H, OCH_2), 3.39 (s, 6 H, OCH_3), 1.50 (s, 6 H, CH_3). $^{13}C\{^{1}H\}$ NMR (125.8 MHz, $CDCl_3$) δ 160.1, 159.5, 150.6, 145.8, 139.3, 139.1 138.5, 138.0, 133.4, 130.0, 129.9, 129.5, 129.4, 128.2, 128.1, 116.9, 115.9, 115.1, 82.9, 72.1,

71.1, 70.8, 70.7, 69.9, 67.7, 59.3, 32.7, 17.9. HRMS (ESI): *m/z* calcd for $C_{56}H_{61}BBr_2F_2I_2N_2O_9Na$ $[M+Na]^+$ 1391.0783, found 1391.0788.

Preparation of 11

A mixture of BHQ-3 amine **9** (5.0 mg, 7.5 μmol), 4-nitrophenyl carbonate-modified BCN **10** (4.6 mg, 14.6 μmol), and triethylamine (6.7 μL, 48.2 μmol) in DMF was stirred at room temperature for 4 h. The solvent was then removed in vacuo. The residue was purified by reserved-phase HPLC with the conditions used for the purification of **3**. The collected fraction was lyophilized to afford **11** as a dark blue solid (4.1 mg, 65%). ^{1}H NMR (500 MHz, CD_3OD) δ 8.27 (d, *J* = 9.0 Hz, 1 H, Ar-H), 8.11 (d, *J* = 9.0 Hz, 1 H, Ar-H), 8.08 (d, *J* = 9.0 Hz, 1 H, Ar-H), 7.94–7.95 (m, 3 H, Ar-H), 7.86 (d, *J* = 9.0 Hz, 1 H, Ar-H), 7.65–7.68 (m, 4 H, Ar-H), 7.24 (s, 1 H, Ar-H), 6.75 (d, *J* = 9.0 Hz, 2 H, Ar-H), 5.81 (s, 1 H, Ar-H), 4.15 (d, *J* = 8.5 Hz, 2 H, OCH_2), 3.80 (br s, 2 H, NCH_2), 3.53 (t, *J* = 7.0 Hz, 2 H, NCH_2), 3.40 (br s, 2 H, NCH_2), 3.17 (t, *J* = 7.0 Hz, 2 H, NCH_2), 3.10 (s, 3 H, NCH_3), 2.14–2.29 (m, 6 H, CH_2), 1.82–1.84 (m, 2 H, CH_2), 1.55–1.65 (m, 2 H, CH_2), 1.29–1.39 (m, 4 H, CH and CH_3), 1.08 (br s, 3 H, CH_3), 0.92–0.97 (m, 2 H, CH_2). ^{13}C{^{1}H} NMR (125.8 MHz, CD_3OD) δ 159.4, 157.9, 156.7, 154.8, 145.2, 144.9, 140.7, 139.2, 137.3, 135.8, 135.1, 133.8, 133.0, 132.9, 128.9, 127.9, 124.9, 121.8, 112.9, 112.0, 99.6, 93.2, 63.7, 50.9, 47.9, 39.3, 38.9, 30.2, 28.4, 22.0, 21.4, 19.0, 14.0, 11.7. HRMS (ESI): *m/z* calcd for $C_{43}H_{48}N_7O_2^+$ $[M]^+$ 694.3864, found 694.3871.

Preparation of 12

Peptide **3** (8.0 mg, 3.8 μmol) and DSBDP **8** (5.2 mg, 3.8 μmol) were dissolved in a mixture of DMF and borate buffer (pH 10.0) (9:1 v/v) (at 1 mM). After stirring at room temperature for 30 min, the mixture was analyzed and the product **12** was purified by reverse-phase HPLC. The conditions used were set as follows: solvent A = 0.1% TFA in acetonitrile and solvent B = 0.1% TFA in deionized water. Elution gradient: 20% A + 80% B in the first 5 min, changed to 100% A + 0% B in 30 min, maintained at 100% A for 5 min, changed to 20% A + 80% B in 10 min, and maintained at 20% A + 80% B for further 10 min. A flow rate of 1 or 3 mL min^{-1} was used for analytical or preparative purpose, respectively. HRMS (ESI): *m/z* calcd for $C_{147}H_{223}BF_2I_2N_{34}O_{29}S_2$ $[M+4H]^{4+}$ 824.1158, found 824.1154.

Preparation of 13

According to the above procedure using peptide **5** (6.0 mg, 3.5 μmol) and DSBDP **8** (4.8 mg, 3.5 μmol) as the starting materials, cyclic peptide **13** was isolated by reverse-phase HPLC. HRMS (ESI): *m/z* calcd for $C_{122}H_{181}BF_2I_2N_{31}O_{29}S_2$ $[M+3H]^{3+}$ 970.7084, found 970.7075.

Preparation of 1

BCN-substituted BHQ-3 **11** (1.3 mg, 1.9 μmol) was added into the above HPLC-purified **12** in a mixture of acetonitrile and water. After complete consumption of **12** as indicated by reverse-

phase HPLC, the target product **1** was isolated with the same conditions described for the purification of **12** and then lyophilized to give a dark bluish-green solid (1.2 mg, 8% for the two-step reaction). HRMS (ESI): *m/z* calcd for $C_{190}H_{271}BF_2I_2N_{41}O_{31}S_2^+$ $[M+4H]^{5+}$ 798.3704, found 798.3701.

Preparation of 14

According to the above procedure using BCN-substituted BHQ-3 **11** (1.2 mg, 1.8 μmol) and the HPLC-purified **13** as the starting materials, conjugate **14** was purified and then lyophilized to give a dark bluish-green solid (1.1 mg, 10% for the two-step reaction). HRMS (ESI): *m/z* calcd for $C_{165}H_{230}BF_2I_2N_{38}O_{31}S_2^+$ $[M+4H]^{5+}$ 721.5039, found 721.5028.

Monitoring the Course of the Formation of 1 and 14

The course of the formation of **1** and **14** was monitored by LC-MS. Reverse-phase LC-MS analysis was performed on a XBridge BEH300 C18 column (5 μm, 4.6 mm × 150 mm) using a Waters ACQUITY Arc system equipped with a Waters 2998 photodiode array detector, a Waters 2475 fluorescence detector, and a Waters SQD 2 mass spectrometer. The conditions used were set as follows: solvent A = 0.01% formic acid in acetonitrile and solvent B = 0.01% formic acid in deionized water. Elution gradient: 20% A + 80% B in the first 5 min, changed to 100% A + 0% B in 30 min, maintained at 100% A for 5 min, changed to 20% A + 80% B in

10 min, and maintained at 20% A + 80% B for further 10 min. A flow rate of 0.8 mL min^{-1} was used for analytical purpose.

Determination of Fluorescence Quantum Yields

The fluorescence quantum yields (Φ_F) of the samples were determined by the equation:

$$\Phi_{F\ (sample)} = \left(\frac{F_{sample}}{F_{ref}}\right)\left(\frac{A_{ref}}{A_{sample}}\right)\left(\frac{n_{sample}^2}{n_{ref}^2}\right)\Phi_{F(ref)}$$

where *F*, *A*, and *n* are the measured fluorescence (area under the emission band), the absorbance at the excitation wavelength (610 nm), and the refractive index of the solvent, respectively.[R4] Unsubstituted ZnPc in DMF was used as the reference [$\Phi_{F(ref)}$ = 0.28].[R5]

Determination of Singlet Oxygen Quantum Yields

The singlet oxygen quantum yields (Φ_Δ) were measured in DMF using the method of chemical quenching of DPBF with ZnPc as the reference [$\Phi_{\Delta(ref)}$ = 0.56].[R5] A solution of DPBF (30 μM) and **1**, **8**, or **14** (all 1 μM) in DMF was irradiated with red light from a 100 W halogen lamp after passing through a water tank for cooling and a color glass filter with a cut-on wavelength at 610 nm (Newport). The absorbance of the DPBF's absorption at 415 nm was monitored along with the irradiation time. The Φ_Δ values were determined by the equation:

$$\Phi_{\Delta(sample)} = \left(\frac{W^{sample}\, I_{abs}^{ref}}{W^{ref}\, I_{abs}^{sample}}\right)\Phi_\Delta^{ref}$$

where W and I_{abs} are the DPBF photobleaching rate and the rate of light absorption, respectively.[R6]

MMP-2 and Cathepsin B-Responsive Spectroscopic Studies

Compound **1** or **14** was dissolved in DMF to form a 1 mM stock solution, which was then diluted to 1 μM with PBS at pH 7.4 or buffer solution (pH 5.0, 25 mM NaOAc, 1 mM EDTA, 500 μM GSH), both in the presence of 0.1% Tween 80 (v/v). For the MMP-2-responsive studies, the solutions in PBS were treated with MMP-2 (2 μg mL^{-1}). For the cathepsin B-responsive studies, the solutions in buffer solution were treated with cathepsin B (1 unit mL^{-1}). For the dual-enzyme-responsive studies, the solutions in PBS were first treated with MMP-2 (2 μg mL^{-1}) for 6 h, followed by the treatment with cathepsin B (1 unit mL^{-1}) after the addition of NaOAc, EDTA, and GSH and adjustment of the pH, turning into the conditions of the buffer solution. All of the solutions were stirred continuously at 37 °C. Their electronic absorption and fluorescence spectra were monitored over a period of 25 h.

MMP-2- and Cathepsin B-Responsive Singlet Oxygen Generation Studies

According to the above procedure, **1** and **14** were treated with MMP-2 and cathepsin B. The solutions were then mixed with a solution of DPBF in DMF (3 mM, 3 μL), giving a final concentration of 30 μM. The mixtures were irradiated with red light coming from a 100 W

halogen lamp after passing through a water tank for cooling and a color glass filter (Newport) cut-on at λ = 610 nm. The decay of DPBF at λ = 417 nm was monitored with a spectrophotometer during the irradiation period.

Monitoring the Reactions of 1 with MMP-2 and/or Cathepsin B

According to the above procedure, **1** and **14** were treated with MMP-2 and cathepsin B. After a period of 25 h, MeOH was added into the solutions for precipitating the enzymes. After filtration, the filtrate was analyzed by LC-MS using the aforementioned conditions.

Cell Lines and Culture Conditions

A549 human lung carcinoma cells (ATCC, no. CCL-185) and HEK-293 human embryonic kidney normal cells (ATCC, no. CRL-1573) were maintained in Dulbecco's Modified Eagle's Medium (DMEM) (ThermoFisher Scientific, cat. no. 12100-046) supplemented with fetal bovine serum (FBS) (10%, Invitrogen, cat. no. 10270-106) and penicillin-streptomycin (100 units mL^{-1} and 100 μg mL^{-1}, respectively). HeLa human cervical carcinoma cells (ATCC, no. CCL-2) were maintained in Roswell Park Memorial Institute (RPMI) 1640 medium (Invitrogen, cat. no. 23400-021) supplemented with FBS (10%) and penicillin-streptomycin (100 units mL^{-1} and 100 μg mL^{-1}, respectively). U-87 MG human glioblastoma cells (ATCC, no. HTB-14) were maintained in Minimum Essential Medium (MEM) (Sigma-Aldrich, no. M5650)

supplemented with FBS (10%) and penicillin-streptomycin (100 units mL^{-1} and 100 μg mL^{-1}, respectively). All the cells were grown at 37 °C in a humidified 5% CO_2 atmosphere.

Confocal Fluorescence Microscopic Studies

Approximately 2 × 10^5 cells in the corresponding medium (2 mL) were seeded on a confocal dish and incubated overnight at 37 °C in a humidified 5% CO_2 atmosphere. Compound **1** or **14** (20 nmol) was first dissolved in DMF (20 μL) to prepare a stock solution of 1 mM, which was diluted with a serum-free medium to 1 μM. For the enzyme-responsive studies, the cells were incubated in the medium (1 mL) with or without SB-3CT (10 μM) and/or CA-074 Me (25 μM) for 2 h. The cells were then incubated with **1** (2 μM) in the same medium for a further 1 h. As a negative control, the cells were simply incubated with **14** (2 μM) in the medium (1 mL) for 1 h. After being rinsed with PBS twice, the cells were further incubated in the medium for 6 h. The cells were then rinsed with PBS twice before being examined with a Leica TCS SP8 high-speed confocal microscope equipped with a 638 nm laser. The fluorescence was monitored at 650−750 nm. The images were digitized and analyzed using a Leica Application Suite X software.

Flow Cytometric Studies

Approximately 2 × 10^5 cells in the corresponding medium (2 mL) were seeded on a 6-well plate and incubated overnight at 37 °C in a humidified 5% CO_2 atmosphere. After the

treatments as described above, the cells were rinsed with PBS twice and then harvested by 0.25% trypsin-EDTA (Invitrogen, 0.3 mL) for 5 min. The activity of trypsin was quenched with a serum-containing medium (0.7 mL), and the mixture was centrifuged at 1500 rpm for 3 min at room temperature. The pellet was washed with PBS (1 mL) and then subject to centrifugation. The cells were suspended in PBS (1 mL) and the intracellular fluorescence intensities were measured using a BD FACSVerse flow cytometer (Becton Dickinson) with 1×10^4 cells counted in each sample. The compounds were excited by an argon laser at 640 nm and the emitted fluorescence was monitored at 720−840 nm. The data collected were analyzed using the BD FAC-Suite. All experiments were performed in triplicate.

Subcellular Localization Studies

Approximately 2×10^5 A549 cells in DMEM (2 mL) were seeded on a confocal dish and incubated overnight at 37 °C in a humidified 5% CO_2 atmosphere. A stock solution of PMB **1** (1 mM) was prepared as described above, which was then diluted to 1 μM with a serum-free medium. The medium was removed and the cells were rinsed with PBS twice. The cells were first incubated with **1** (1 μM) for 1 h. After being rinsed with PBS twice, the cells were incubated in a serum-free medium for 6 h. The cells were rinsed with PBS twice, followed by staining with LysoTracker Green DND-26 (Thermo Fisher Scientific Inc., L7526) (2 μM for 30 min), MitoTracker Green FM (Thermo Fisher Scientific Inc., M7514) (0.2 μM for 15 min),

or ER-Tracker Green (Thermo Fisher Scientific Inc., E34251) (1 μM for 15 min) in a serum-free medium at 37 °C. The solutions were then removed, and the cells were rinsed with PBS twice before being examined with a Leica TCS SP8 high-speed confocal microscope equipped with a 488 nm laser and a 638 nm laser. All the trackers were excited at 488 nm and their fluorescence was monitored at 500−570 nm, while the DSBDP moiety was excited at 638 nm and its fluorescence was monitored at 650−750nm. The images were digitized and analyzed using a Leica Application Suite X software.

Intracellular ROS Generation Studies

Approximately 2 × 10^5 A549 cells in DMEM or HeLa cells in RPMI 1640 medium (2 mL) were seeded on a confocal dish and incubated overnight at 37 °C in a humidified 5% CO_2 atmosphere. A stock solution of **1** or **14** (1 mM) was prepared as described above, which was diluted to 1 μM with a serum-free medium. The cells were treated as described above. After being rinsed by PBS twice, the cells were incubated with H_2DCFDA in PBS (10 μM, 1 mL) at 37 °C for 30 min. The cells were rinsed and refilled with PBS (1 mL) before being irradiated ($\lambda > 610$ nm, 23 mW cm^{-2}, 14 J cm^{-2}) at ambient temperature. The cells were examined with a Leica TCS SP8 high-speed confocal microscope equipped with a solid-state 488 nm laser. DCF, the oxidized product of H_2DCFDA, was excited at 488 nm and its fluorescence was monitored

at 500−550 nm. The images were digitized and analyzed using a Leica Application Suite X software.

Photocytotoxicity Assay

Approximately 2 × 10^4 cells per well (100 μL) in the corresponding medium were inoculated in 96-well plates and incubated overnight at 37 °C in a humidified 5% CO_2 atmosphere. A stock solution of **1** or **14** (1 mM) was prepared as described above, which was diluted with a serum-free medium to various concentrations. The cells, after being rinsed with PBS twice, were treated with or without SB-3CT (10 μM) and/or CA-074 Me (25 μM) in the medium (100 μL) for 2 h. The cells were further incubated with 100 μL of different concentrations of **1** or **14** for 1 h at 37 °C under 5% CO_2. After being rinsed with PBS twice, the cells were incubated in the culture medium (100 μL) for 6 h. The cells were then rinsed with PBS and re-fed with 100 μL of a serum-containing medium before being irradiated (λ > 610 nm, 23 mW cm^{-2}, 28 J cm^{-2}) at ambient temperature. Cell viability was determined by means of a colorimetric MTT assay.[R7] After irradiation, the cells were incubated at 37 °C under 5% CO_2 overnight. After incubation, the medium was removed and the cells were rinsed with PBS twice. A MTT (Sigma) solution in PBS (3 mg mL^{-1}, 50 μL) was added to each well followed by incubation for 4 h under the same environment. DMSO (100 μL) was then added to each well. Solutions in all the wells were mixed until homogenous. The absorbance at 490 nm of each well on the plate was

taken by a microplate reader (Tecan Spark 10M) at ambient temperature. The average absorbance of the blank wells, which did not contain the cells, was subtracted from the readings of the other wells. The cell viability was then determined by the equation:

$$\% \text{ viability} = \frac{\Sigma\left(\frac{A_\text{i}}{A_\text{control}}\right)\times 100}{n}$$

where A_i is the absorbance of the ith datum (i = 1, 2, …, n), A_control is the average absorbance of the control wells in which the compound was absent, and n (= 4) is the number of data points.

In Vivo Imaging

Female Balb/c nude mice (20–25 g) were obtained from the Laboratory Animal Services Centre at The Chinese University of Hong Kong. All animal experiments were approved by the Animal Experimentation Ethics Committee of the City University of Hong Kong. The mice were kept under a pathogen-free condition with free access to food and water. A549 cells (1×10^7 cells in 200 μL) were inoculated subcutaneously at the back of the mice. Once the tumors had grown to a size of 80–100 mm^3, **1** or **14** (20 nmol) dissolved in 20 μL of distilled water containing 7.5% DMSO and 0.5% Tween 80 (v/v) was injected to the tumor-bearing mice through intratutormal injection. In vivo fluorescence imaging was performed before and after the injection at different time points up to 24 h with an Odyssey infrared imaging system (excitation wavelength = 680 nm, emission wavelength ≥ 700 nm). The images were digitized

and analyzed using an Odyssey imaging system software (no. 9201-500). Four mice were used for each compound.

In Vivo PDT

A549 tumor-bearing nude mice were prepared as described above. The length and width of the tumor were measured using a micrometer digital caliper (SCITOP Systems). The tumor volume (mm^3) was calculated using the formula: tumor volume = (length × width^2)/2. Once the tumors had grown to a size of 80–100 mm^3, **1** or **14** (20 nmol) dissolved in 20 μL of distilled water containing 7.5% DMSO and 0.5% Tween 80 (v/v) was injected to the tumor-bearing mice through intratumoral injection. At 6 h post-injection, the tumor was illuminated with a diode laser (Biolitec Ceralas) at 680 nm operated at 0.24 W. Illumination on a circular spot with a diameter of 1.0 cm (fluence rate = 0.3 W cm^{-2}) for 10 min led to a total fluence of 180 J cm^{-2}. The tumor size of the nude mice was monitored periodically for the next 14 days. The tumor volumes were also compared with a PBS control group of mice without the light treatment. Four mice were used for each group. On Day 14 after the PDT treatment, the mice were sacrificed, and the tumor and major organs were harvested. The tissues were fixed with 4% paraformaldehyde, dehydrated in an alcohol series, mixed with solutions of xylene and paraffin, and embedded in paraffin. The tissue sections were prepared by a Leica RM2235 microtome.

After the H&E staining, the tissues were examined with a Carl Zeiss PALM inverted microscope.

In Vivo Photodynamic Effect on Skin

Female Balb/c nude mice (20–25 g) were injected with a solution of **1** or **6** (20 nmol) in 200 μL of distilled water containing 3% DMSO and 0.5% Tween 80 (v/v) through the tail vein. In vivo fluorescence imaging was performed before and after the injection at different time points up to 24 h with an Odyssey infrared imaging system (excitation wavelength = 680 nm, emission wavelength ≥ 700 nm). The images were digitized and analyzed using an Odyssey imaging system software (no. 9201-500). Three mice were used for each compound. To study the photodynamic effect of these two compounds on the skin of the mice, a region of the skin was illuminated with a diode laser (Biolitec Ceralas) at 680 nm operated at 0.24 W at 6 h post-injection of these compounds. Illumination on a circular spot with a diameter of 1.0 cm (fluence rate = 0.3 W cm^{-2}) for 10 min led to a total fluence of 180 J cm^{-2}. After 24 h, the mice were sacrificed, and then the skin tissues were harvested. They were fixed with 4% paraformaldehyde, dehydrated in an alcohol series, mixed with solutions of xylene and paraffin, and then embedded in paraffin. The tissue sections were prepared by a Leica RM2235 microtome. After the H&E staining, the tissues were examined with a Nikon Eclipse Ni-E upright fluorescence microscope. For the TUNEL staining assay, the skin tissues were dewaxed

and rehydrated in an alcohol series and then incubated with a Proteinase K solution (20 μg mL^{-1}) at room temperature for 30 min. After being rinsed with PBS three times, the tissue slides were stained with 50 μL of Nucleotide Mix (a mixture of terminal deoxynucleotidyl transferase and cyanine 3-labeled deoxyuridine triphosphate) of the one-step TUNEL assay kit (Beyotime, Shanghai, China), and then the nuclei were stained with Hochest 33342 (10 μM) for 1 h at room temperature. After being rinsed with PBS for three times, the tissue slides were examined with a Zeiss LSM 880 NLO laser scanning microscope equipped with two solid-state 405 and 561 nm lasers. Hoechst 33342 was excited at 405 nm and its fluorescence was monitored at 410−500 nm, while the TUNEL staining cyanine 3 dye was excited at 561 nm and its fluorescence was monitored at 570−620 nm. The images were digitized and analyzed using a Zen software.

Statistical Analysis

Data shown on figures are presented as the means with the SEM or SD. The data were analyzed using the Student's t-test with p values < 0.05 considered as significant; *p < 0.05; **p < 0.01; and ***p < 0.001. Statistical calculations were performed using a Microsoft Excel spreadsheet (Microsoft Corporation, Redmond, WA, USA).

References

(R1) Ke, M.-R.; Ng, D. K. P.; Lo, P.-C. Synthesis and in vitro photodynamic activities of an integrin-targeting cRGD-conjugated zinc(II) phthalocyanine. *Chem. Asian J.* **2014**, *9*, 554−561.

(R2) Chu, J. C. H.; Shao, C.; Ha, S. Y. Y.; Fong, W.-P.; Wong, C. T. T.; Ng, D. K. P. One-pot peptide cyclisation and surface modification of photosensitiser-loaded red blood cells for targeted photodynamic therapy. *Biomater. Sci.* **2021**, *9*, 7832−7837.

(R3) Dommerholt, J.; Schmidt, S.; Temming, R.; Hendriks, L. J. A.; Rutjes, F. P. J. T.; van Hest, J. C. M.; Lefeber, D. J.; Friedl, P.; van Delft, F. L. Readily accessible bicyclononynes for bioorthogonal labeling and three-dimensional imaging of living cells. *Angew. Chem., Int. Ed.* **2010**, *49*, 9422−9425.

(R4) Eaton, D. F. Reference materials for fluorescence measurement. *Pure Appl. Chem.* **1988**, *60*, 1107−1114.

(R5) Ha, S. Y. Y.; Zhou, Y.; Fong, W.-P.; Ng, D. K. P. Multifunctional molecular therapeutic agent for targeted and controlled dual chemo- and photodynamic therapy. *J. Med. Chem.* **2020**, *63*, 8512−8523.

(R6) Maree, S. E.; Nyokong, T. Syntheses and photochemical properties of octasubstituted phthalocyaninato zinc complexes. *J. Porphyrins Phthalocyanines* **2001**, *5*, 782−792.

(R7) Tada, H.; Shiho, O.; Kuroshima, K.; Koyama, M.; Tsukamoto, K. An improved colorimetric assay for interleukin 2. *J. Immunol. Methods* **1986**, *93*, 157−165.

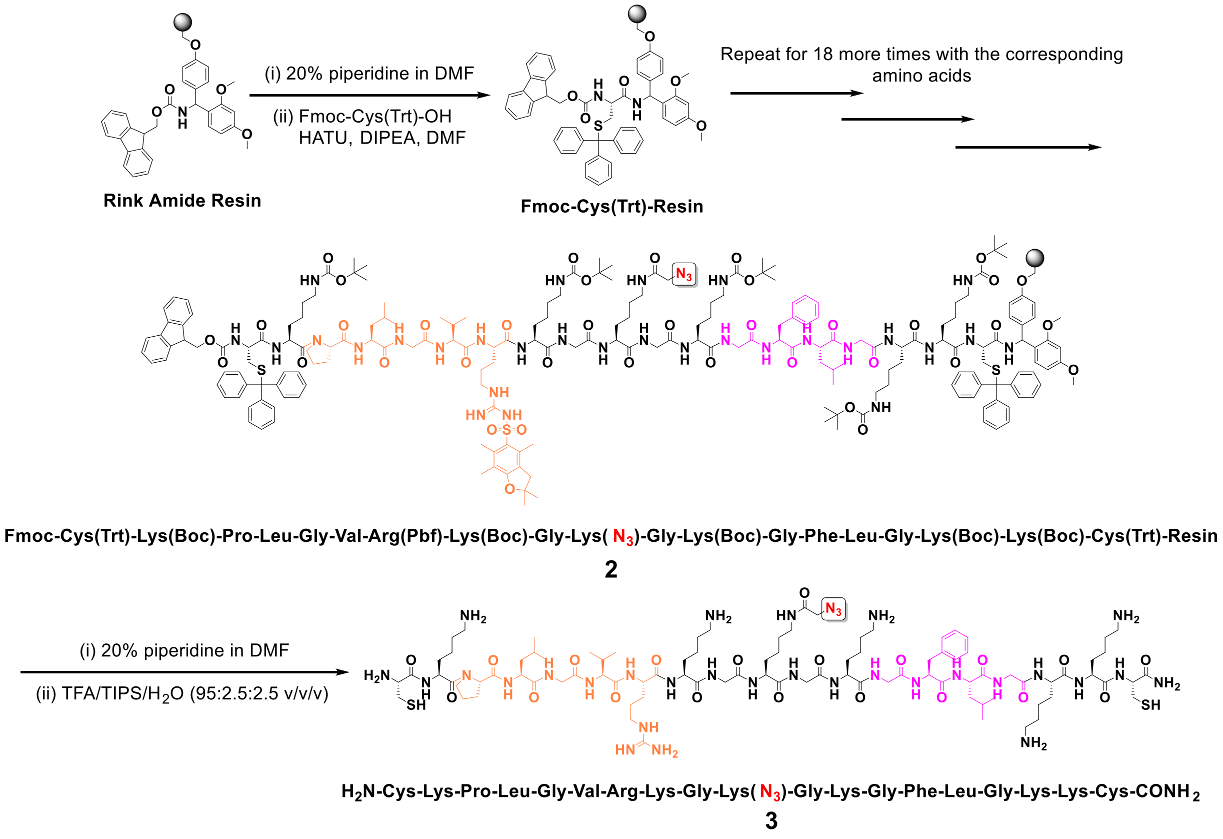

Scheme S1. Synthetic route of the azide-appended peptide **3**.

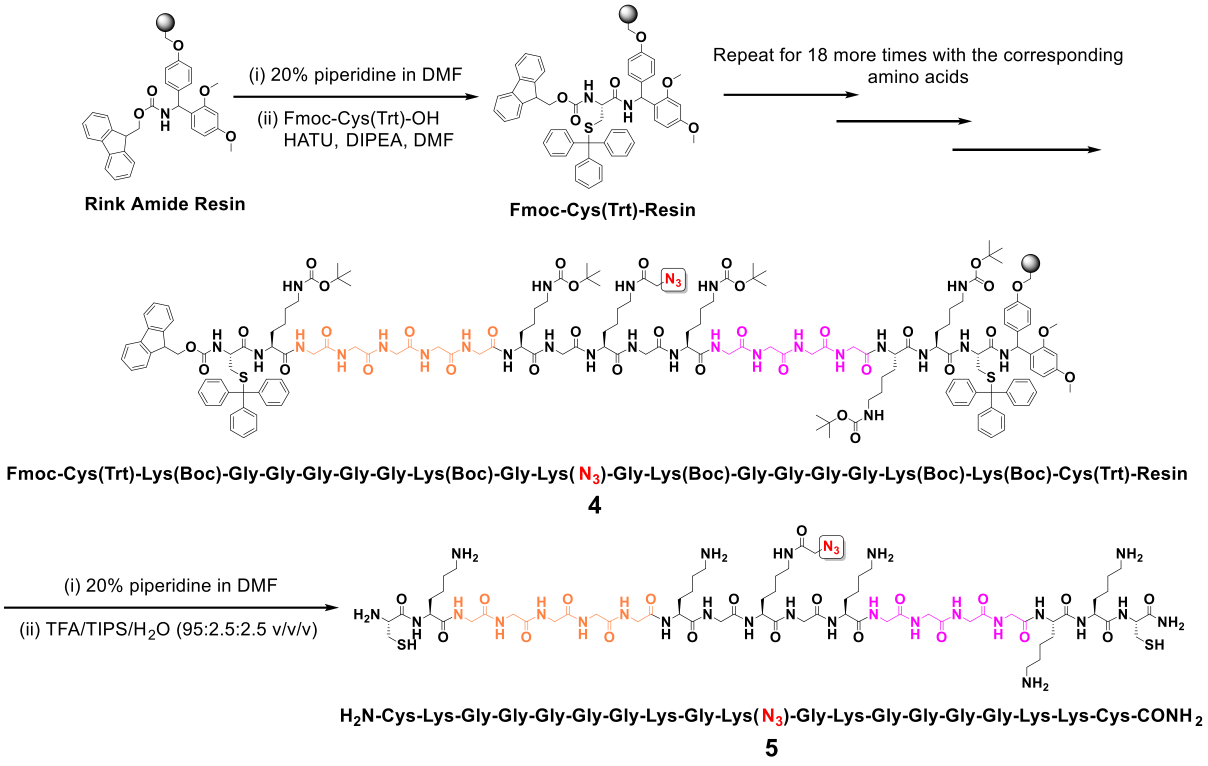

Scheme S2. Synthetic route of the azide-appended peptide **5**.

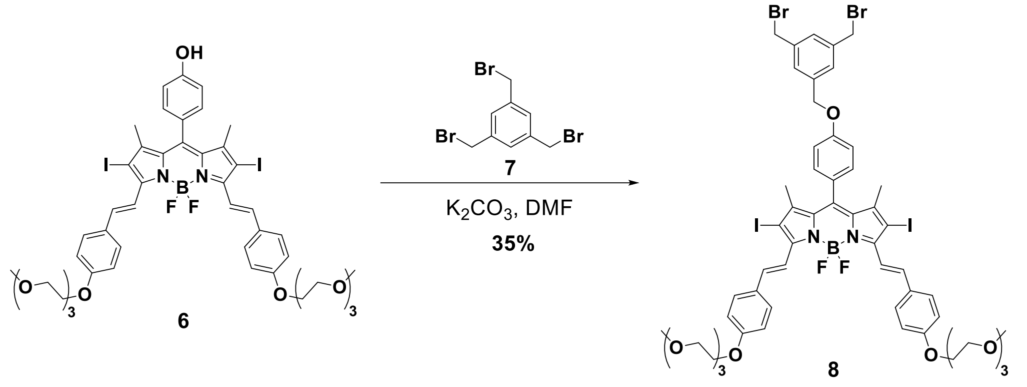

Scheme S3. Synthetic route of 3,5-bis(bromomethyl)phenyl-substituted DSBDP **8**.

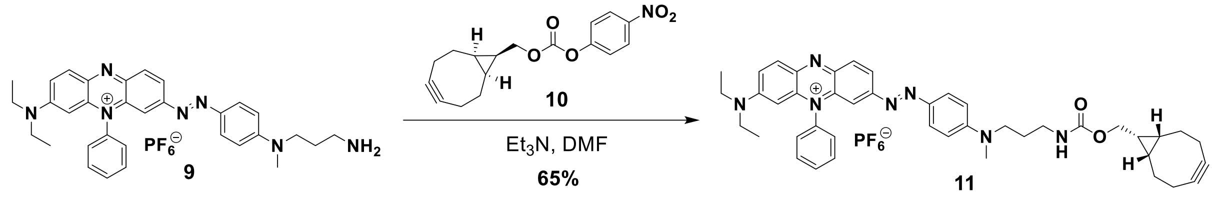

Scheme S4. Synthetic route of BCN-substituted BHQ-3 **11**.

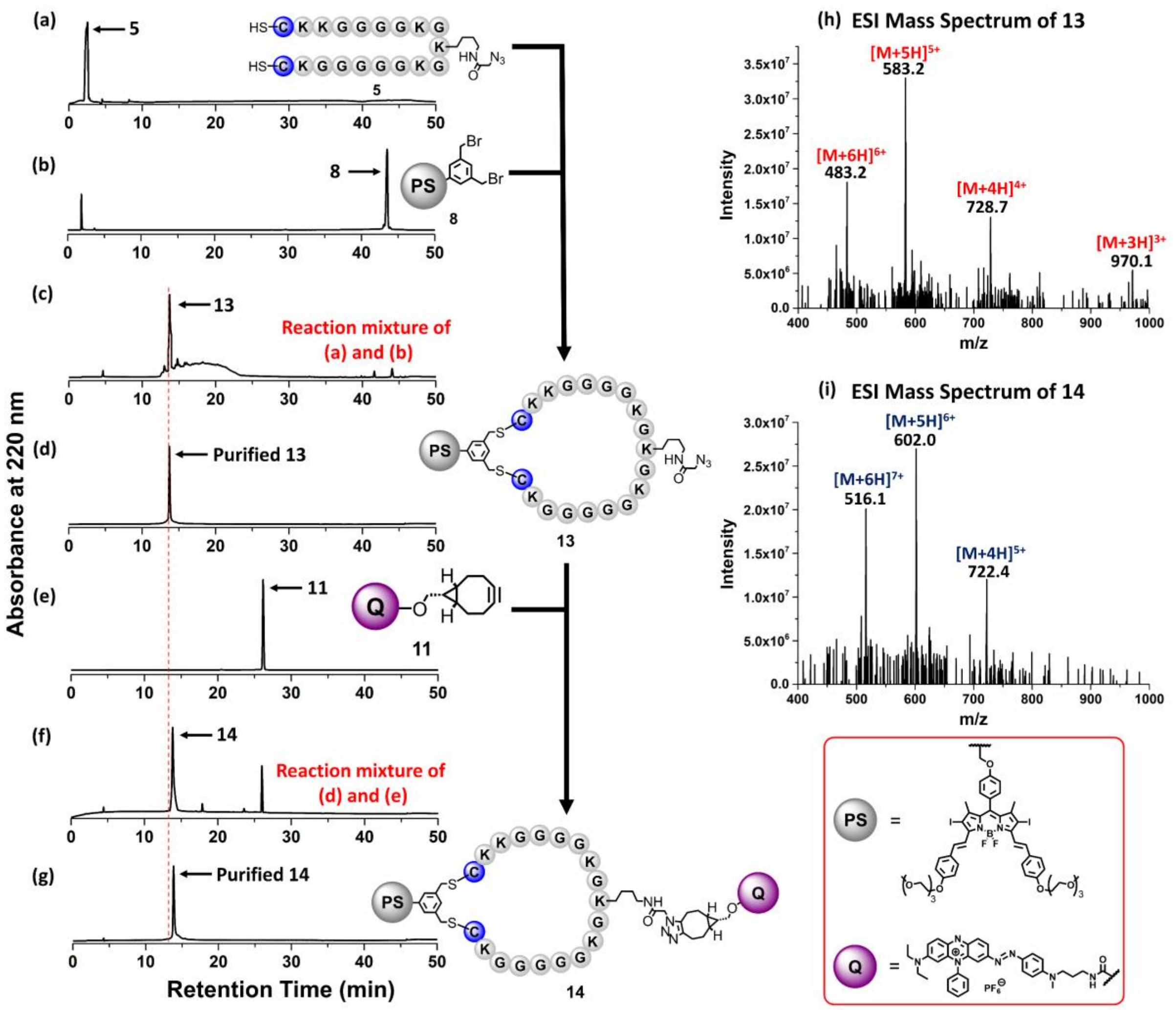

Figure S1. Monitoring the course of the formation of conjugate **14** using LC-MS.

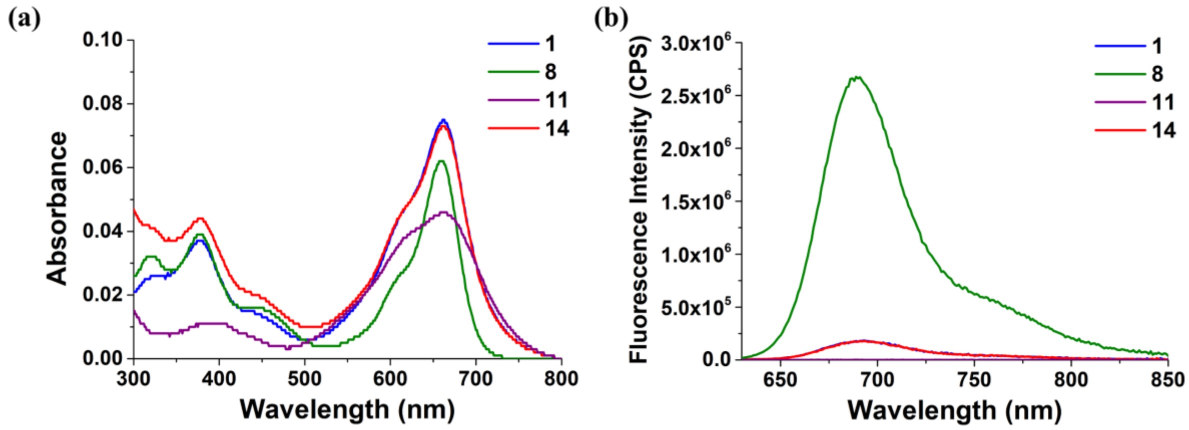

Figure S2. (a) Electronic absorption and (b) fluorescence (λ_{ex} = 610 nm) spectra of **1**, **8**, **11**, and **14** (all at 1 μM) in DMF.

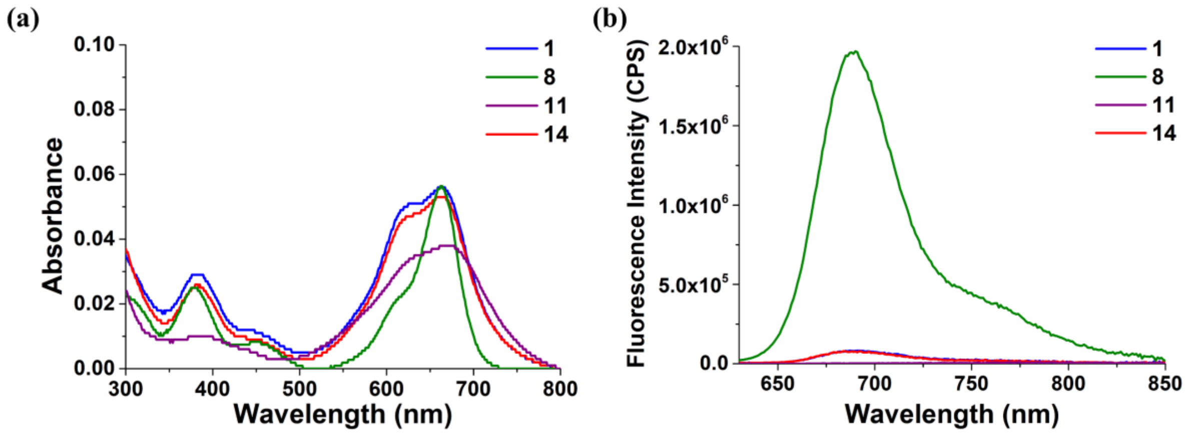

Figure S3. (a) Electronic absorption and (b) fluorescence (λ_{ex} = 610 nm) spectra of **1**, **8**, **11**, and **14** (all at 1 μM) in buffer solution (pH 5.0, 25 mM NaOAc, 1 mM EDTA, 500 μM GSH) with 0.1% Tween 80 (v/v).

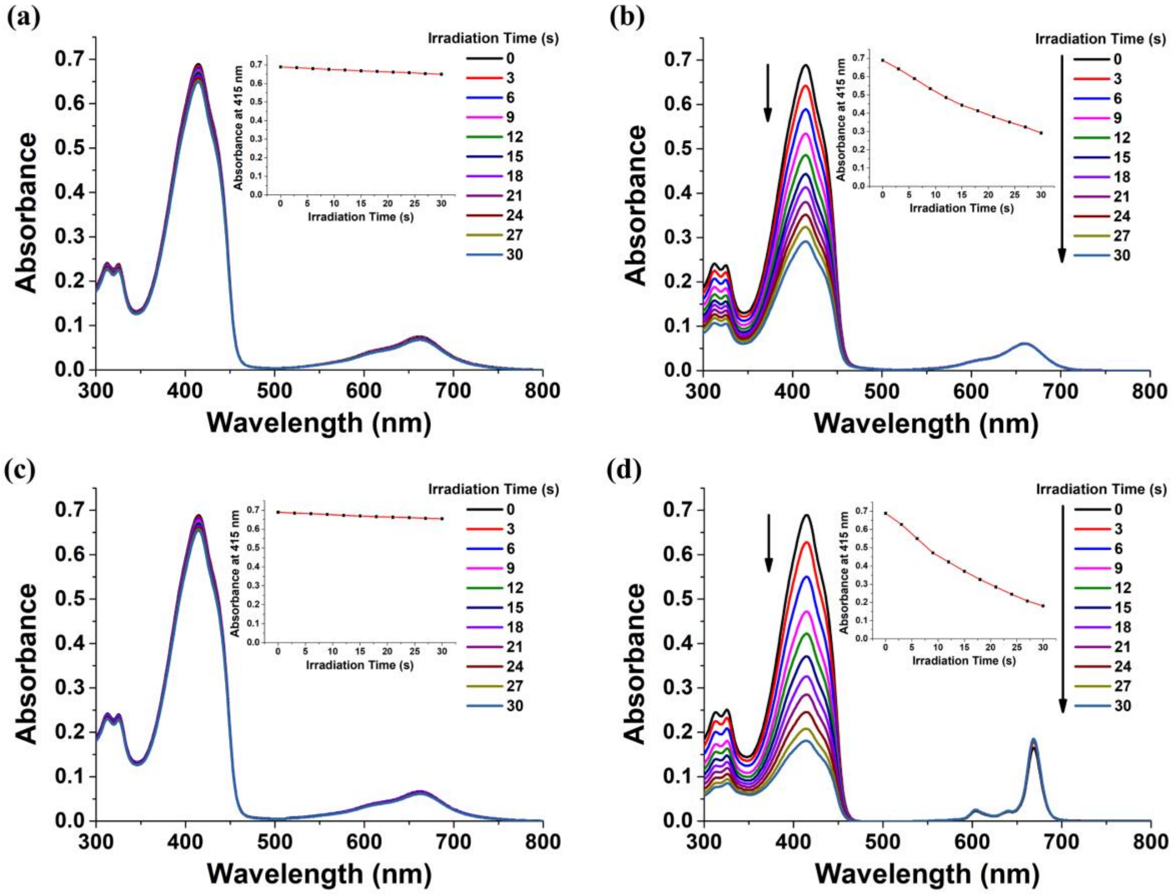

Figure S4. Change in the electronic absorption spectrum of a mixture of DPBF (initial concentration = 30 μM) and (a) **1**, (b) **8**, (c) **14**, or (d) ZnPc in DMF upon irradiation (λ > 610 nm) for 30 s.

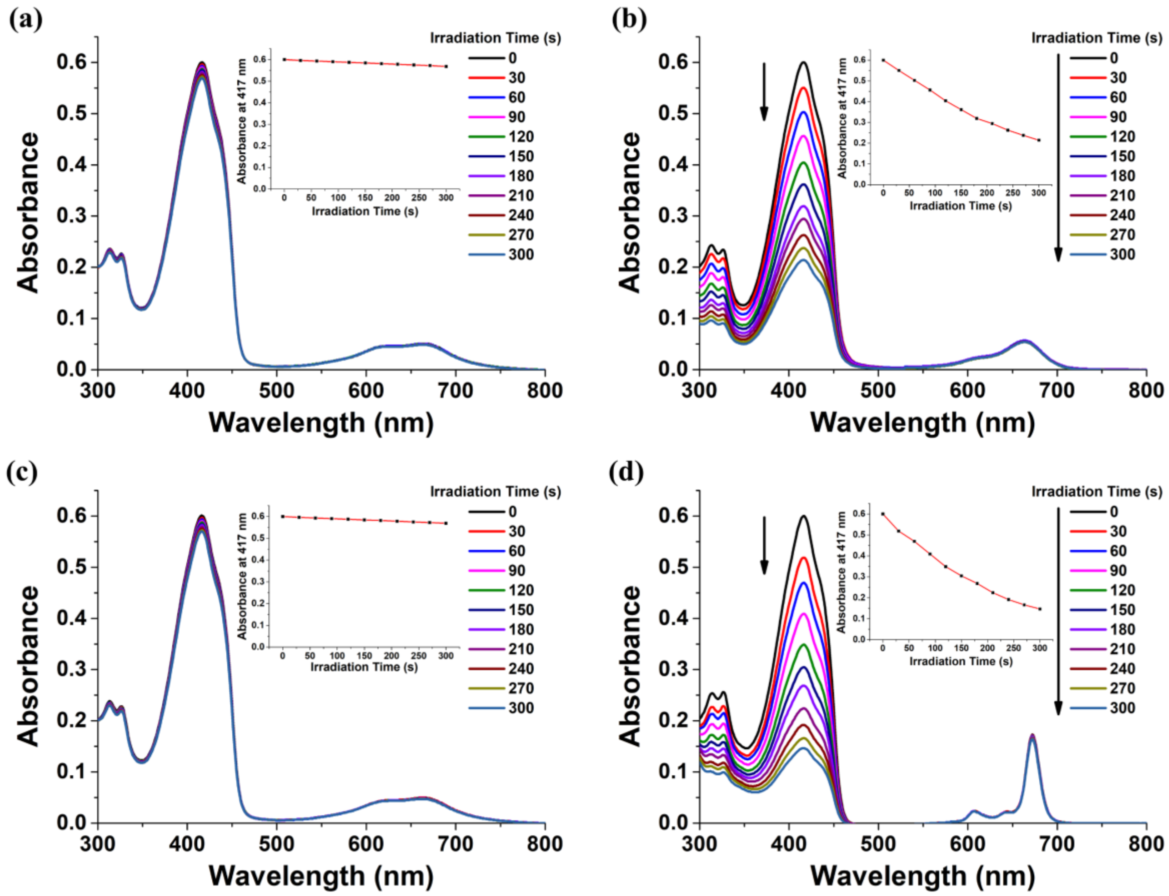

Figure S5. Change in the electronic absorption spectrum of a mixture of DPBF (initial concentration = 30 μM) and (a) **1**, (b) **8**, (c) **14**, or (d) ZnPc in PBS at pH 7.4 with 0.1% Tween 80 (v/v) upon irradiation (λ > 610 nm) for 300 s.

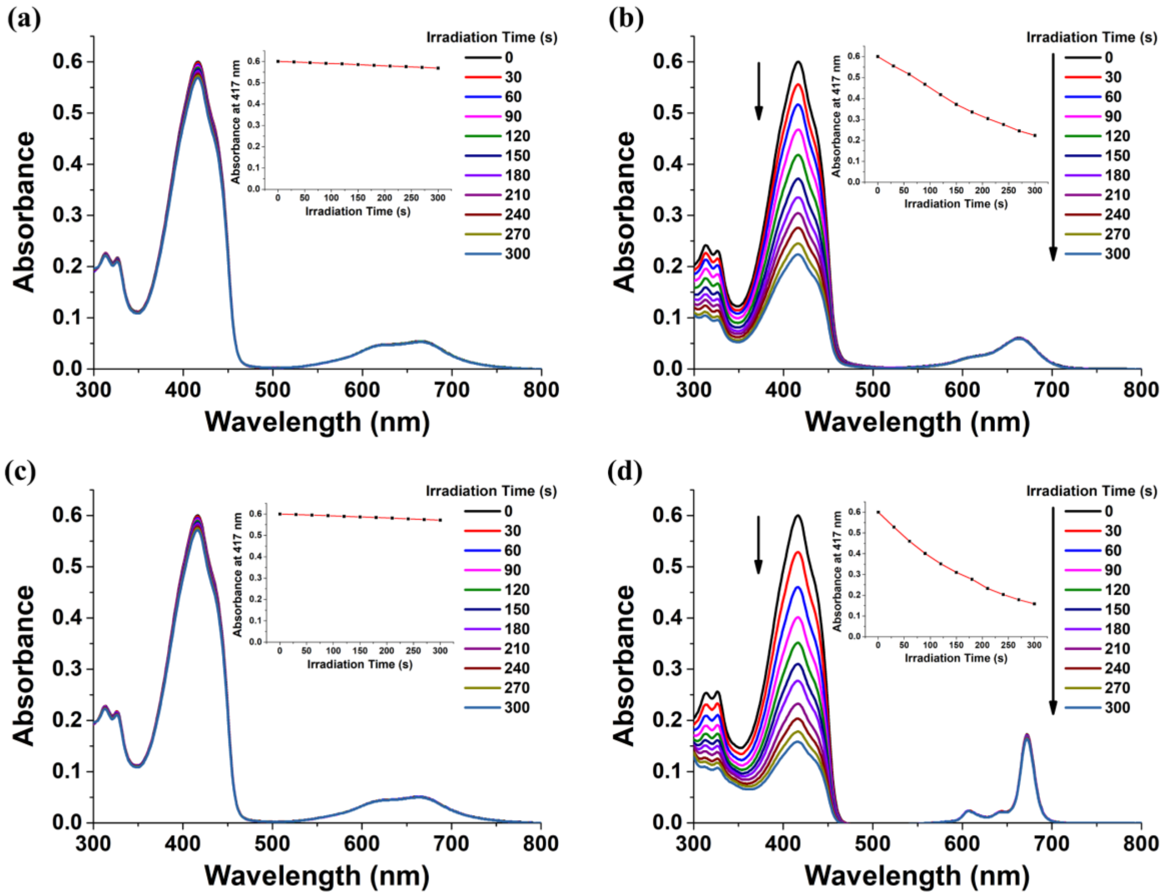

Figure S6. Change in the electronic absorption spectrum of a mixture of DPBF (initial concentration = 30 μM) and (a) **1**, (b) **8**, (c) **14**, or (d) ZnPc in buffer solution (pH 5.0, 25 mM NaOAc, 1 mM EDTA, 500 μM GSH) with 0.1% Tween 80 (v/v) upon irradiation (λ > 610 nm) for 300 s.

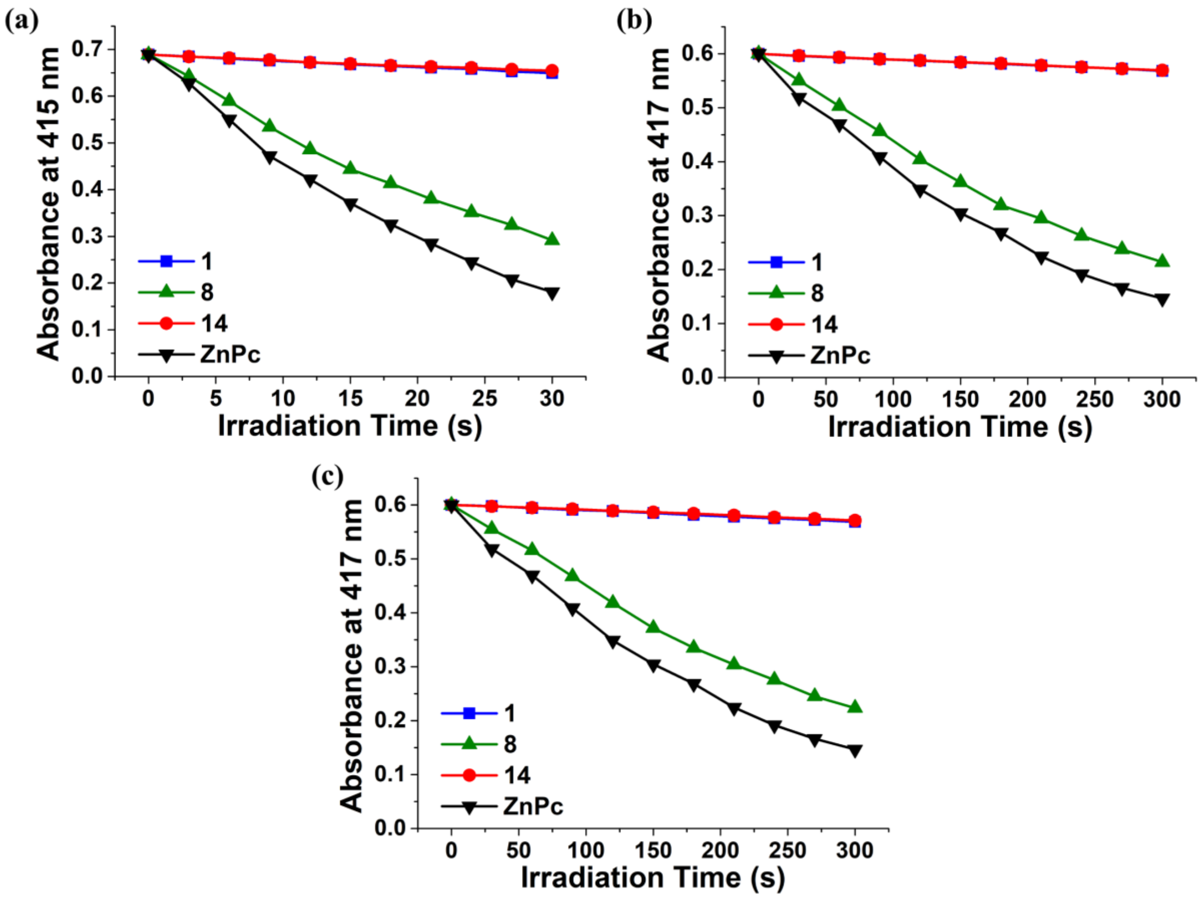

Figure S7. Comparison of the rates of decay of DPBF (initial concentration = 30 μM) sensitized by **1**, **8**, **14**, and ZnPc (all at 1 μM) in (a) DMF, (b) PBS at pH 7.4 with 0.1% Tween 80 (v/v), and (c) buffer solution (pH 5.0, 25 mM NaOAc, 1 mM EDTA, 500 μM GSH) with 0.1% Tween 80 (v/v).

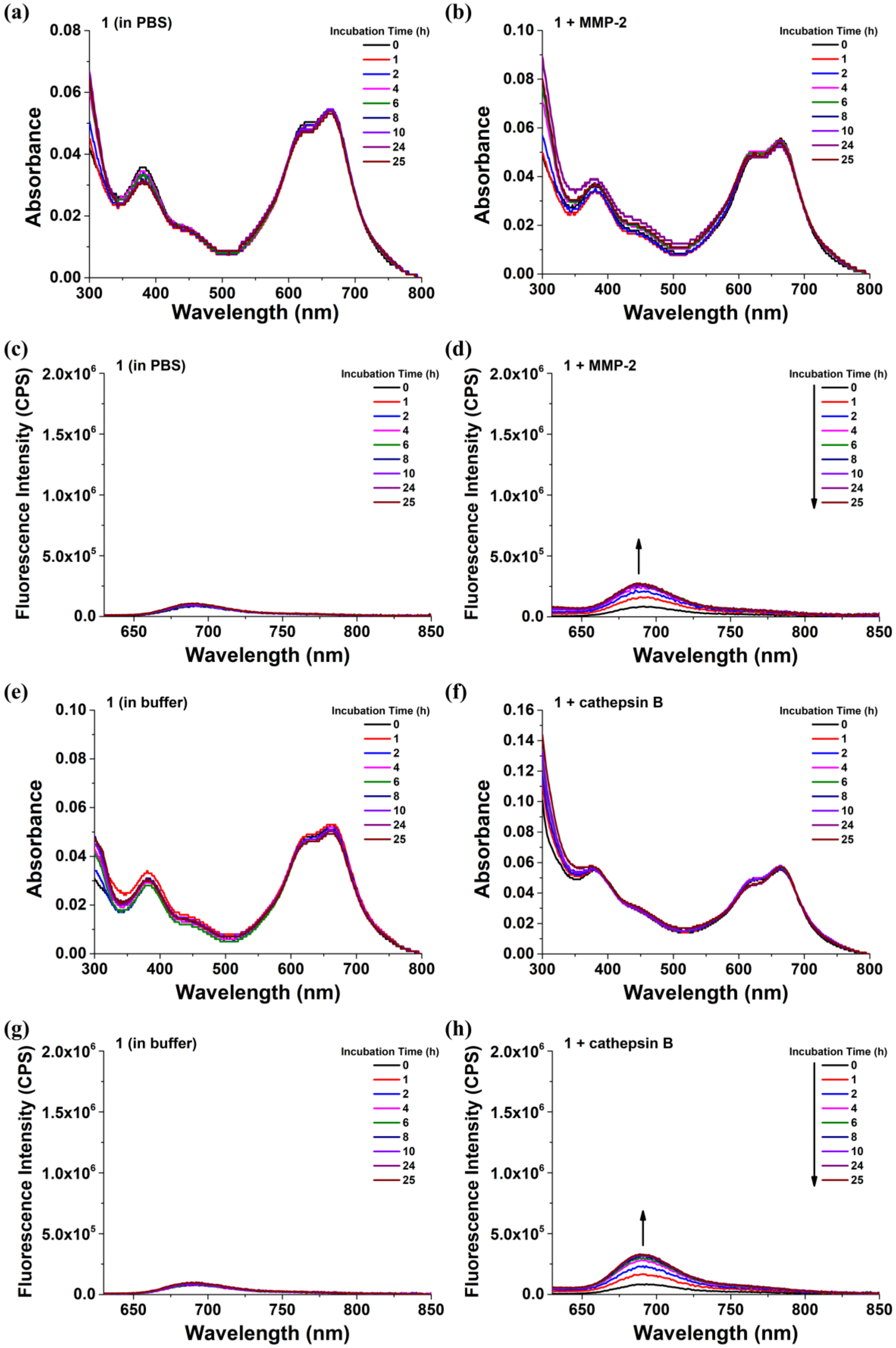
(a)
1 (in PBS)
Absorbance
Wavelength (nm)
Incubation Time (h)
0
1
2
4
6
8
10
24
25
(b)
1 + MMP-2
Absorbance
Wavelength (nm)
(c)
1 (in PBS)
Fluorescence Intensity (CPS)
Wavelength (nm)
(d)
1 + MMP-2
Fluorescence Intensity (CPS)
Wavelength (nm)
(e)
1 (in buffer)
Absorbance
Wavelength (nm)
(f)
1 + cathepsin B
Absorbance
Wavelength (nm)
(g)
1 (in buffer)
Fluorescence Intensity (CPS)
Wavelength (nm)
(h)
1 + cathepsin B
Fluorescence Intensity (CPS)
Wavelength (nm)

Figure S8. Change in the (a,b,e,f) electronic absorption and (c,d,g,h) fluorescence (λ_{ex} = 610 nm) spectra of **1** (1 μM) in the absence or presence of MMP-2 (2 μg mL^{-1}) or cathepsin B (1 unit mL^{-1}) in PBS at pH 7.4 with 0.1% Tween 80 (v/v) (for the study involving MMP-2) or buffer solution (pH 5.0, 25 mM NaOAc, 1 mM EDTA, 500 μM GSH) with 0.1% Tween 80 (v/v) (for the study involving cathepsin B) at 37 °C over a period of 25 h.

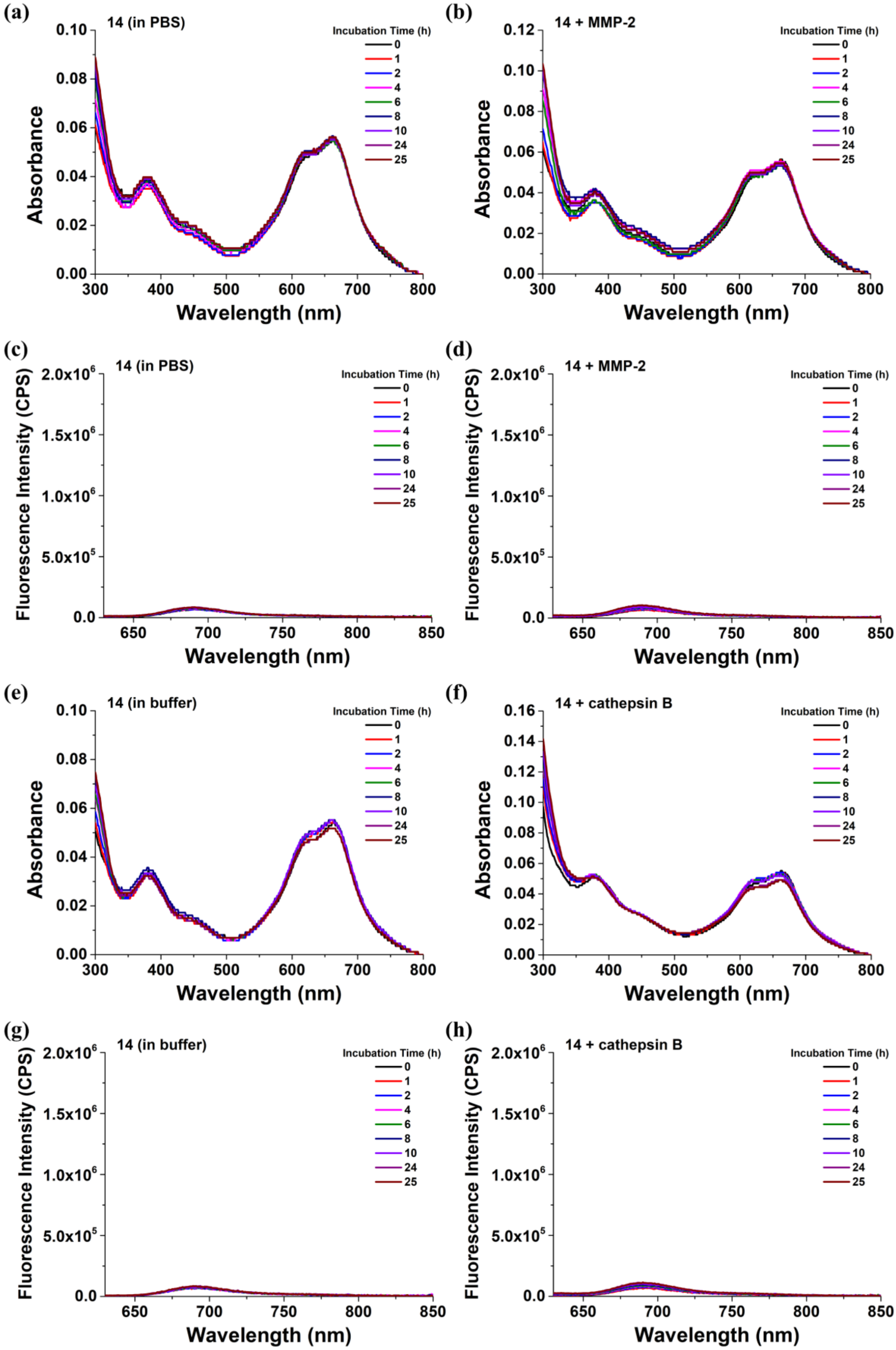

(a)
14 (in PBS)
Absorbance
Wavelength (nm)
Incubation Time (h)
0
1
2
4
6
8
10
24
25
(b)
14 + MMP-2
Absorbance
Wavelength (nm)
Incubation Time (h)
(c)
14 (in PBS)
Fluorescence Intensity (CPS)
Wavelength (nm)
Incubation Time (h)
(d)
14 + MMP-2
Fluorescence Intensity (CPS)
Wavelength (nm)
Incubation Time (h)
(e)
14 (in buffer)
Absorbance
Wavelength (nm)
Incubation Time (h)
(f)
14 + cathepsin B
Absorbance
Wavelength (nm)
Incubation Time (h)
(g)
14 (in buffer)
Fluorescence Intensity (CPS)
Wavelength (nm)
Incubation Time (h)
(h)
14 + cathepsin B
Fluorescence Intensity (CPS)
Wavelength (nm)
Incubation Time (h)

Figure S9. Change in the (a,b,e,f) electronic absorption and (c,d,g,h) fluorescence (λ_{ex} = 610 nm) spectra of **14** (1 μM) in the absence or presence of MMP-2 (2 μg mL^{-1}) or cathepsin B (1 unit mL^{-1}) in PBS at pH 7.4 with 0.1% Tween 80 (v/v) (for the study involving MMP-2) or buffer solution (pH 5.0, 25 mM NaOAc, 1 mM EDTA, 500 μM GSH) with 0.1% Tween 80 (v/v) (for the study involving cathepsin B) at 37 °C over a period of 25 h.

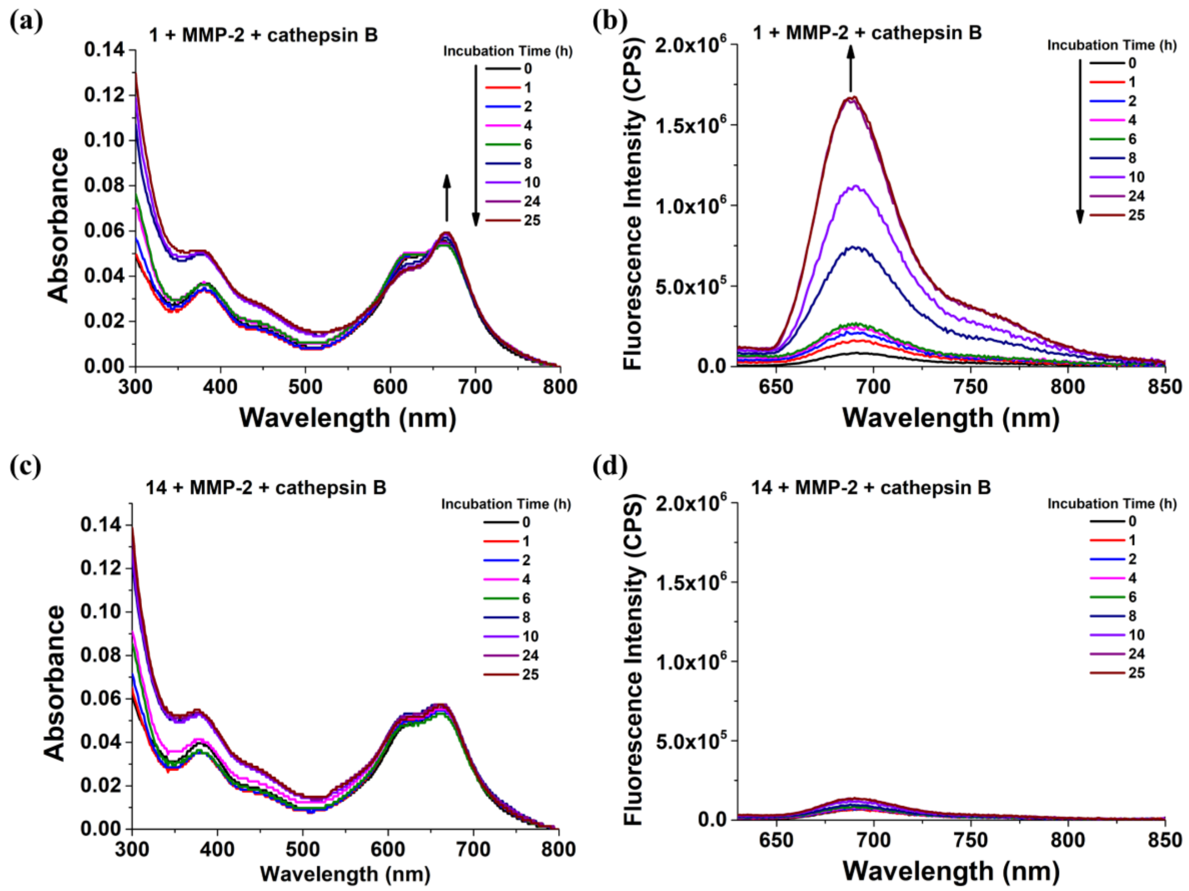

Figure S10. Change in the (a,c) electronic absorption and (b,d) fluorescence (λ_{ex} = 610 nm) spectra of (a,b) **1** and (c,d) **14** (both at 1 μM) upon treatment with MMP-2 (2 μg mL^{-1}) in PBS at pH 7.4 with 0.1% Tween 80 (v/v) at 37 °C for 6 h, and then together with cathepsin B (1 unit mL^{-1}) in buffer solution (pH 5.0, 25 mM NaOAc, 1 mM EDTA, 500 μM GSH) with 0.1% Tween 80 (v/v) at 37 °C for further 19 h.

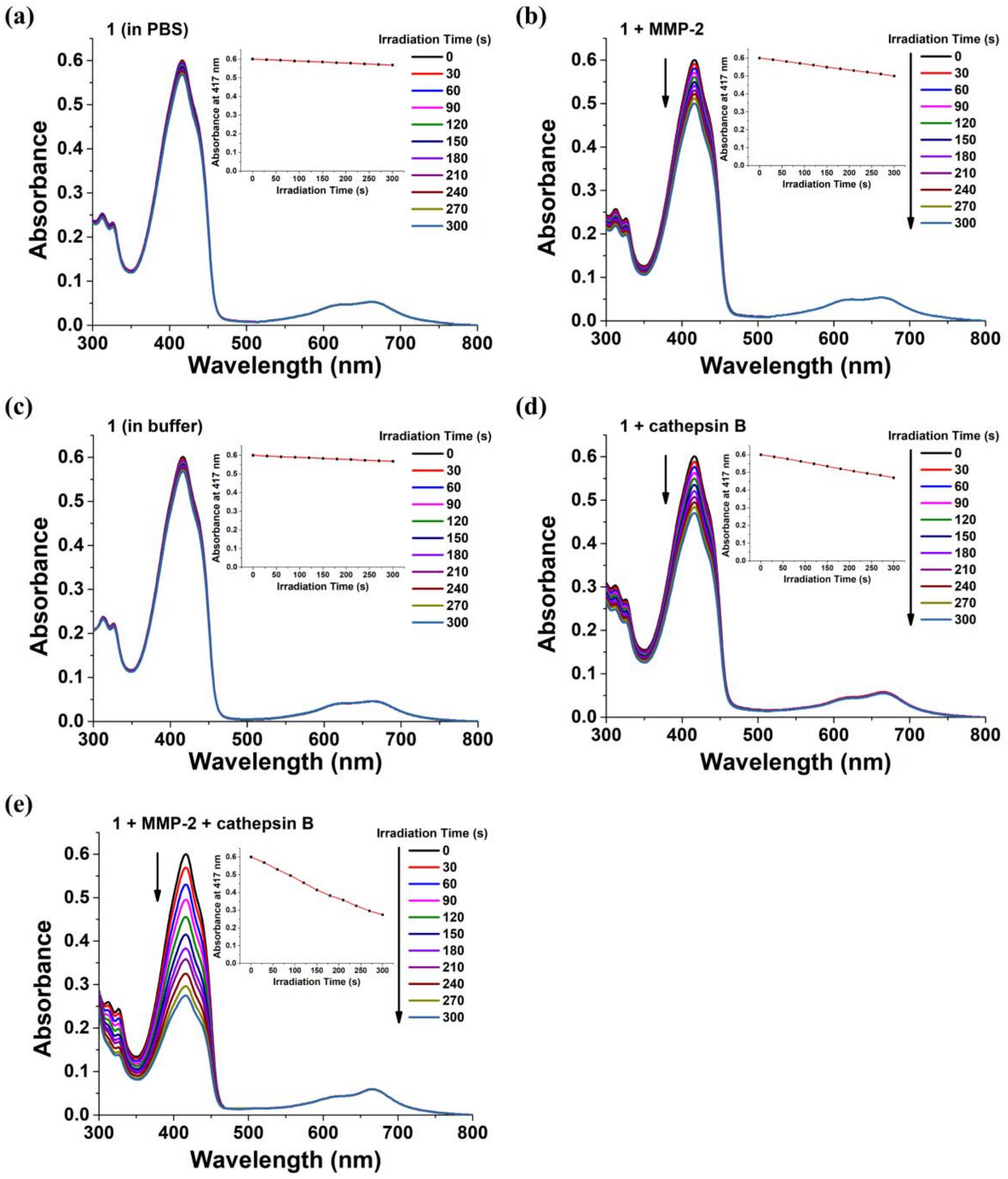

Figure S11. Change in the electronic absorption spectrum of a mixture of DPBF (initial concentration = 30 μM) and **1** (1 μM) after being treated as follows upon light irradiation (λ > 610 nm) over a period of 300 s: in the (a) absence or (b) presence of MMP-2 (2 μg mL^{-1}) in PBS at pH 7.4 with 0.1% Tween 80 (v/v) at 37 °C for 25 h; in the (c) absence or (d) presence

of cathepsin B (1 unit mL^{-1}) in buffer solution (pH 5.0, 25 mM NaOAc, 1 mM EDTA, 500 μM GSH) with 0.1% Tween 80 (v/v) at 37 °C for 25 h; (e) with MMP-2 (2 μg mL^{-1}) in PBS at pH 7.4 with 0.1% Tween 80 (v/v) at 37 °C for 6 h, and then together with cathepsin B (1 unit mL^{-1}) in buffer solution (pH 5.0, 25 mM NaOAc, 1 mM EDTA, 500 μM GSH) with 0.1% Tween 80 (v/v) at 37 °C for further 19 h.

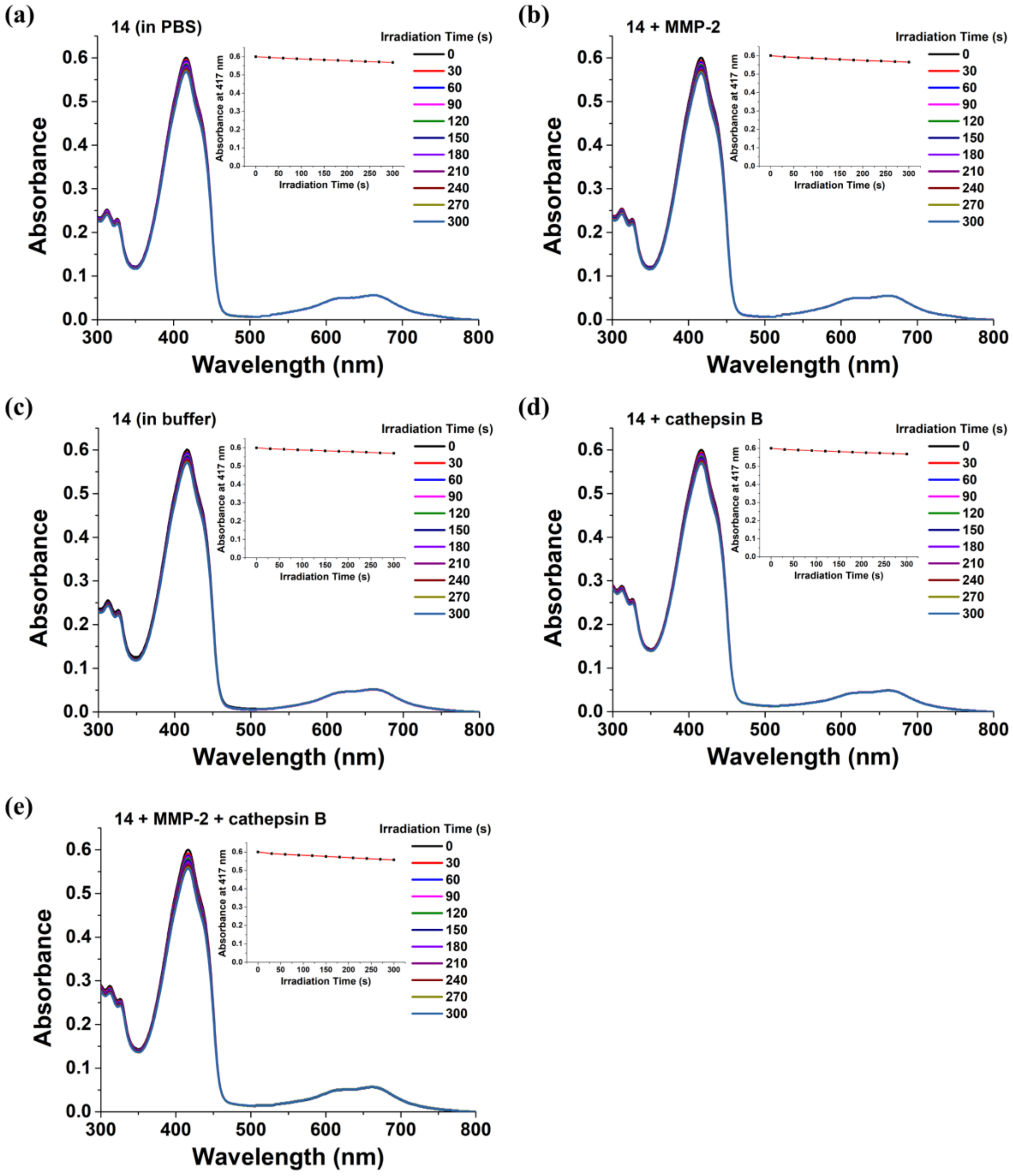

Figure S12. Change in the electronic absorption spectrum of a mixture of DPBF (initial concentration = 30 μM) and **14** (1 μM) after being treated as follows upon light irradiation (λ > 610 nm) over a period of 300 s: in the (a) absence or (b) presence of MMP-2 (2 μg mL^{-1}) in PBS at pH 7.4 with 0.1% Tween 80 (v/v) at 37 °C for 25 h; in the (c) absence or (d) presence

of cathepsin B (1 unit mL^{-1}) in buffer solution (pH 5.0, 25 mM NaOAc, 1 mM EDTA, 500 μM GSH) with 0.1% Tween 80 (v/v) at 37 °C for 25 h; (e) with MMP-2 (2 μg mL^{-1}) in PBS at pH 7.4 with 0.1% Tween 80 (v/v) at 37 °C for 6 h, and then together with cathepsin B (1 unit mL^{-1}) in buffer solution (pH 5.0, 25 mM NaOAc, 1 mM EDTA, 500 μM GSH) with 0.1% Tween 80 (v/v) at 37 °C for further 19 h.

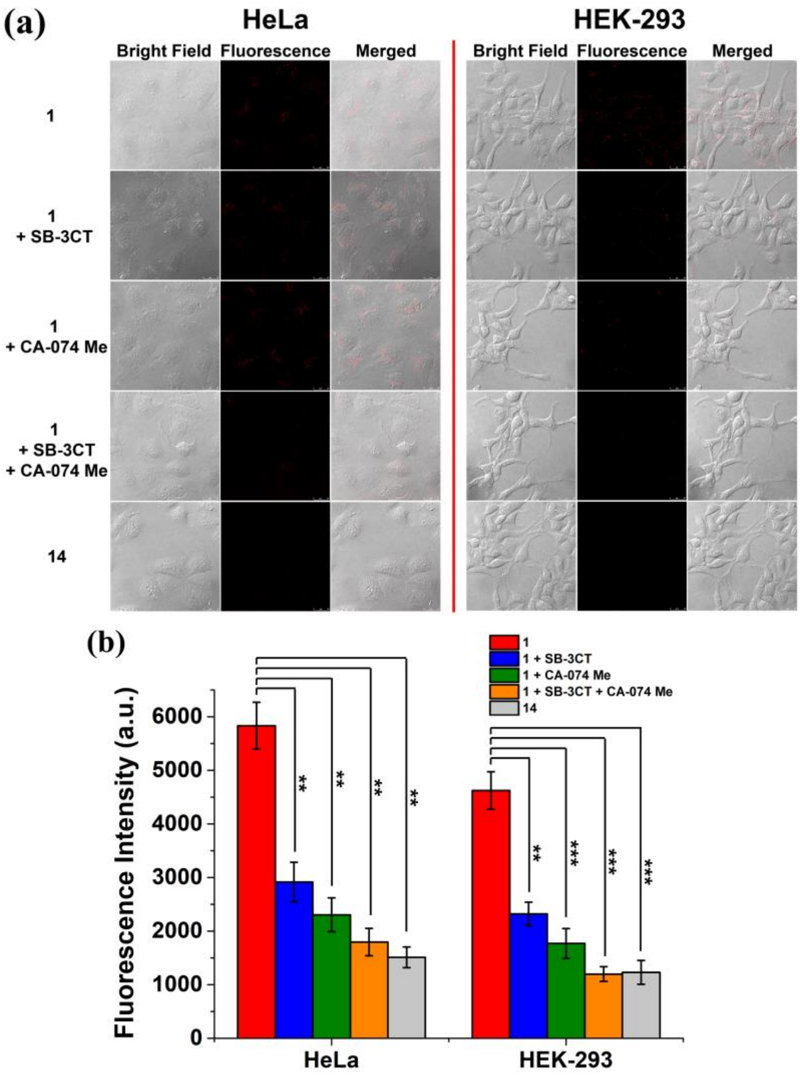

Figure S13. Bright field, fluorescence, and the merged images of HeLa and HEK-293 cells after incubation in a serum-free medium in the absence or presence of SB-3CT (10 μM) and/or CA-074 Me (25 μM) for 2 h, and then with **1** (2 μM) for 1 h, followed by post-incubation in the medium for a further 6 h or incubation with **14** (2 μM) for 1 h, followed by post-incubation in the medium for a further 6 h. (b) The mean intracellular fluorescence intensities of HeLa and HEK-293 cells under these conditions as determined by flow cytometry. Data are expressed as the mean ± SEM of three independent experiments. $^{**}p < 0.01$ and $^{***}p < 0.001$ as calculated by the Student's t-test.

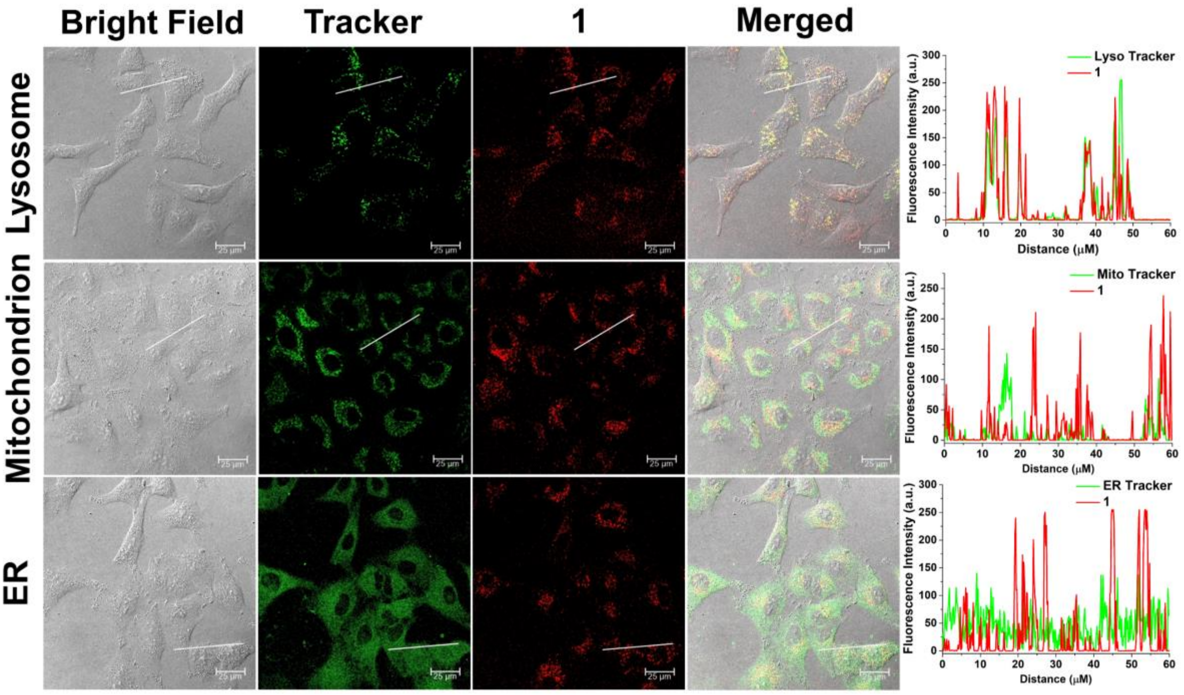

Figure S14. Visualization of the intracellular fluorescence of the DSBDP fragment of **1** and various subcellular trackers in A549 cells. The cells were sequentially incubated with **1** (2 μM) for 1 h, culture medium for 6 h, and then LysoTracker Green DND-26 (2 μM for 30 min), MitoTracker Green FM (0.2 μM for 15 min) or ER-Tracker Green (1 μM for 15 min). The rightmost column shows the fluorescence intensity profiles of the fragment and the trackers traced along the white lines in the corresponding confocal images.

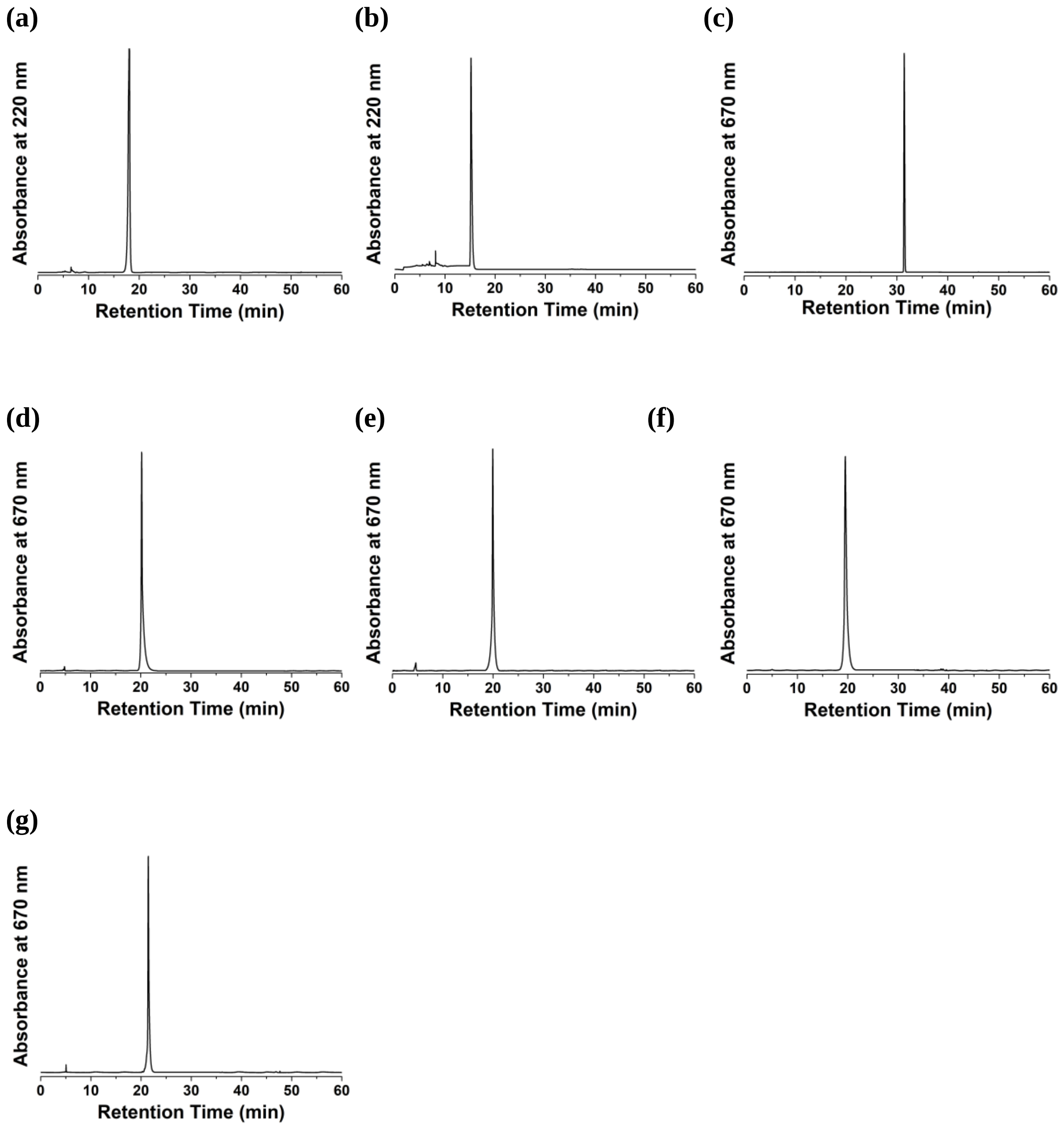

Figure S15. HPLC chromatograms of (a) **3**, (b) **5**, (c) **11**, (d) **12**, (e) **13**, (f) **14**, and (g) **1**.

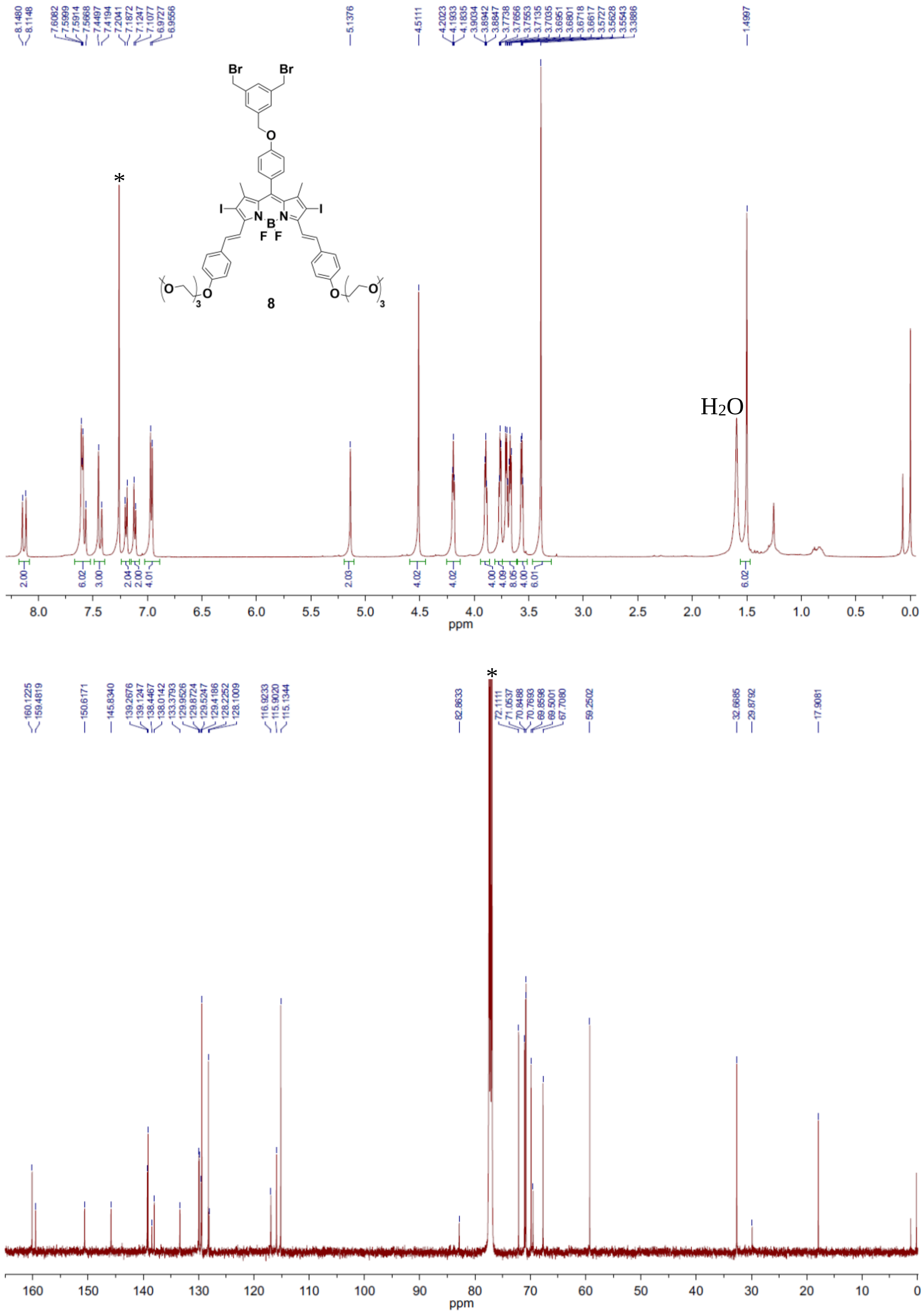

Figure S16. ^{1}H and $^{13}C\{^{1}H\}$ NMR spectra of **8** in $CDCl_3$.

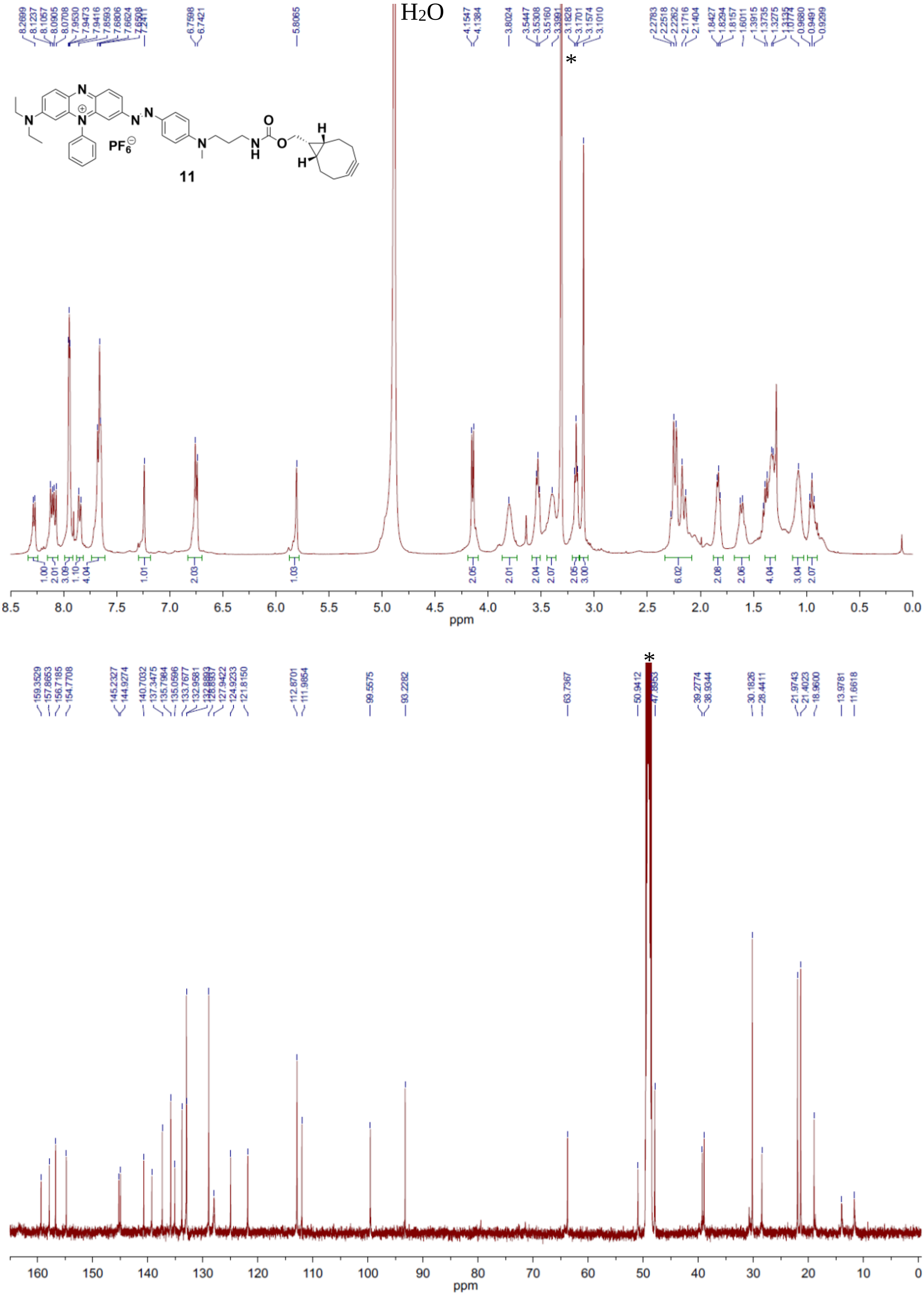

Figure S17. ^{1}H and $^{13}C\{^{1}H\}$ NMR spectra of **11** in CD_3OD.

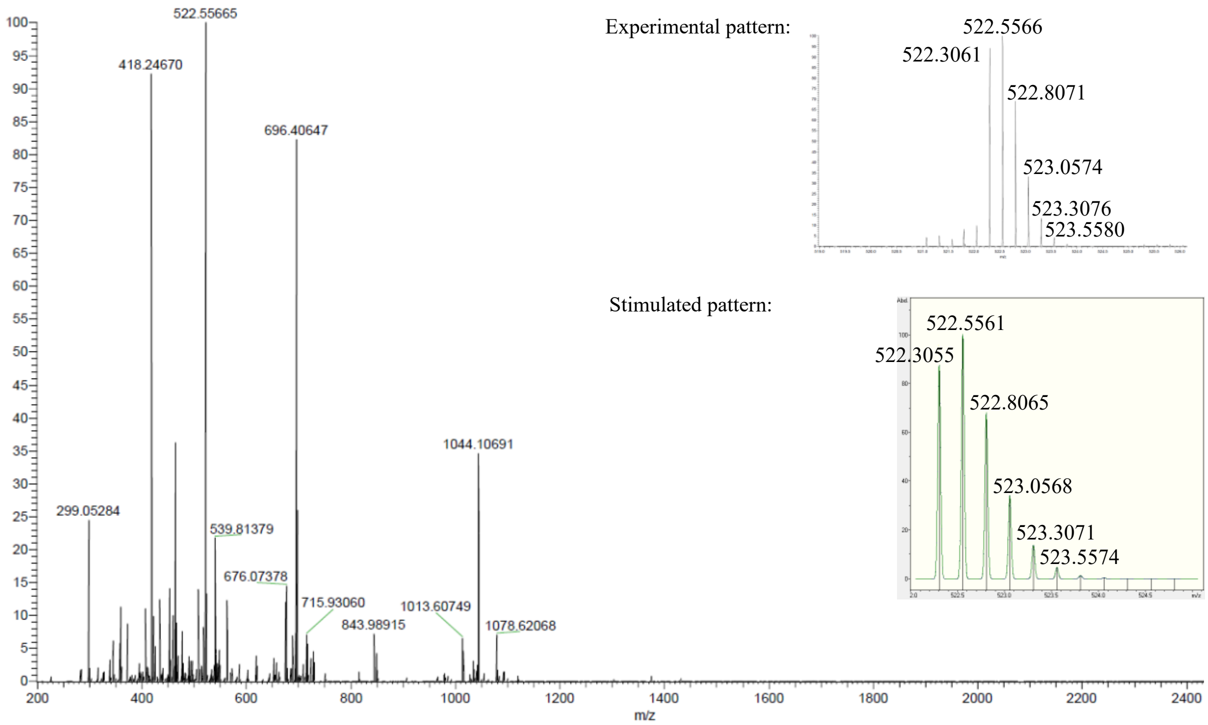

Figure S18. ESI mass spectrum of **3**. The insets show the experimental and simulated isotopic patterns for the $[M+4H]^{4+}$ ion.

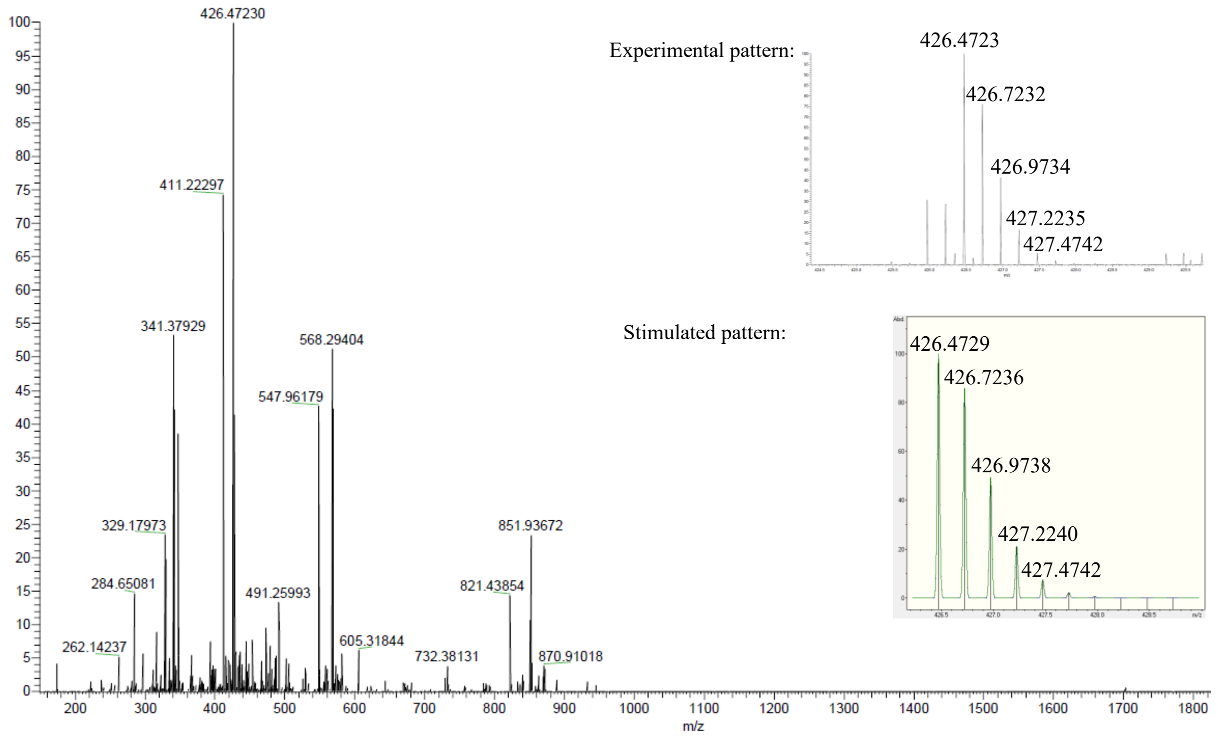

Figure S19. ESI mass spectrum of **5**. The insets show the experimental and simulated isotopic patterns for the $[M+4H]^{4+}$ ion.

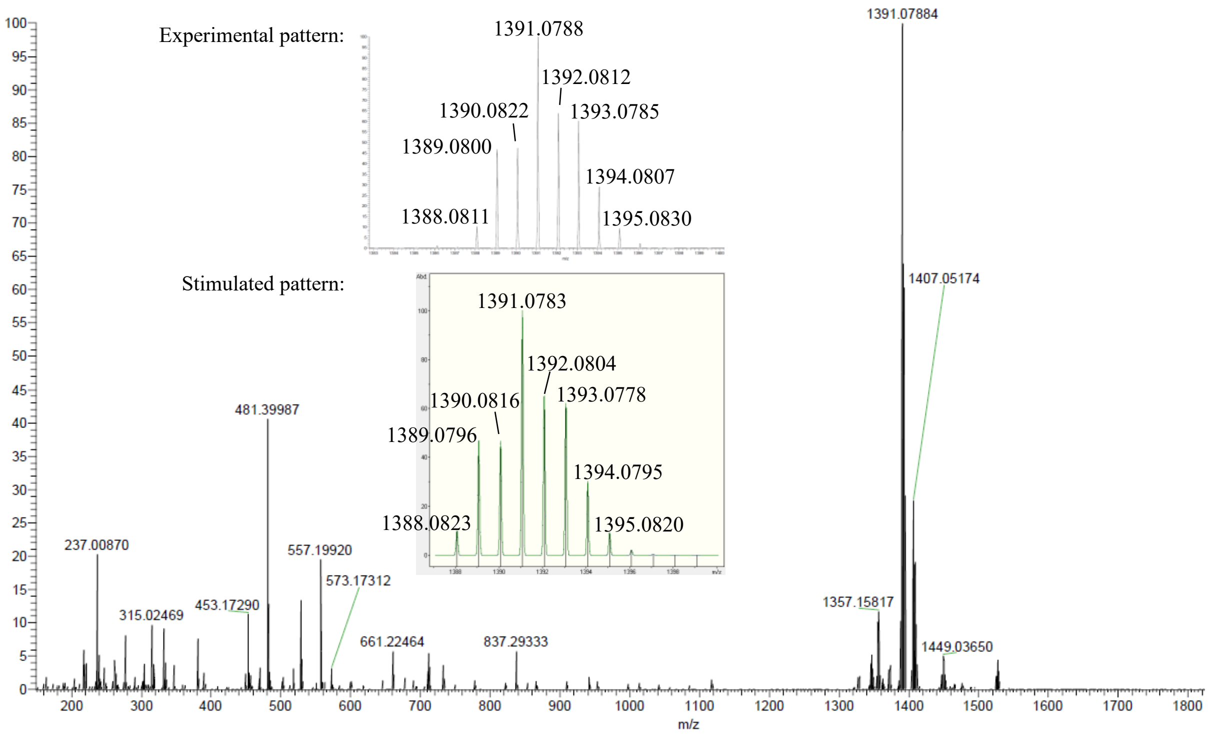

Figure S20. ESI mass spectrum of **8**. The insets show the experimental and simulated isotopic patterns for the $[M+Na]^+$ ion.

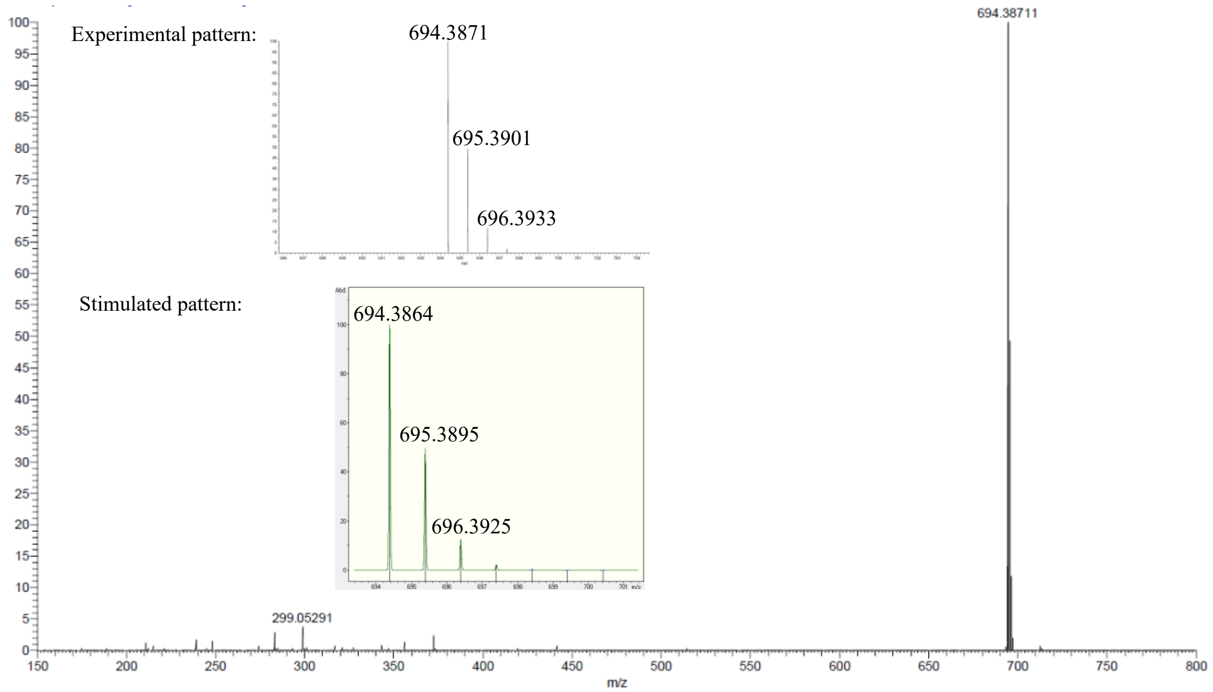

Figure S21. ESI mass spectrum of **11**. The insets show the experimental and simulated isotopic patterns for the $[M]^+$ ion.

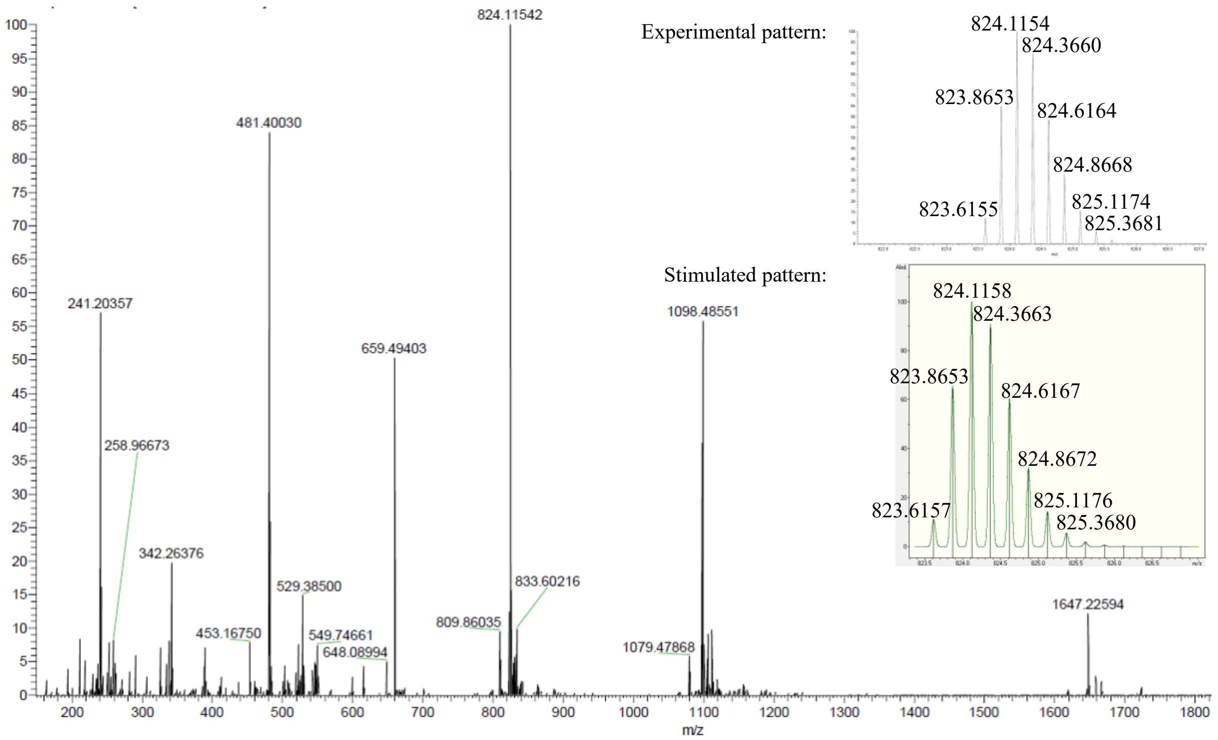

Figure S22. ESI mass spectrum of **12**. The insets show the experimental and simulated isotopic patterns for the $[M+4H]^{4+}$ ion.

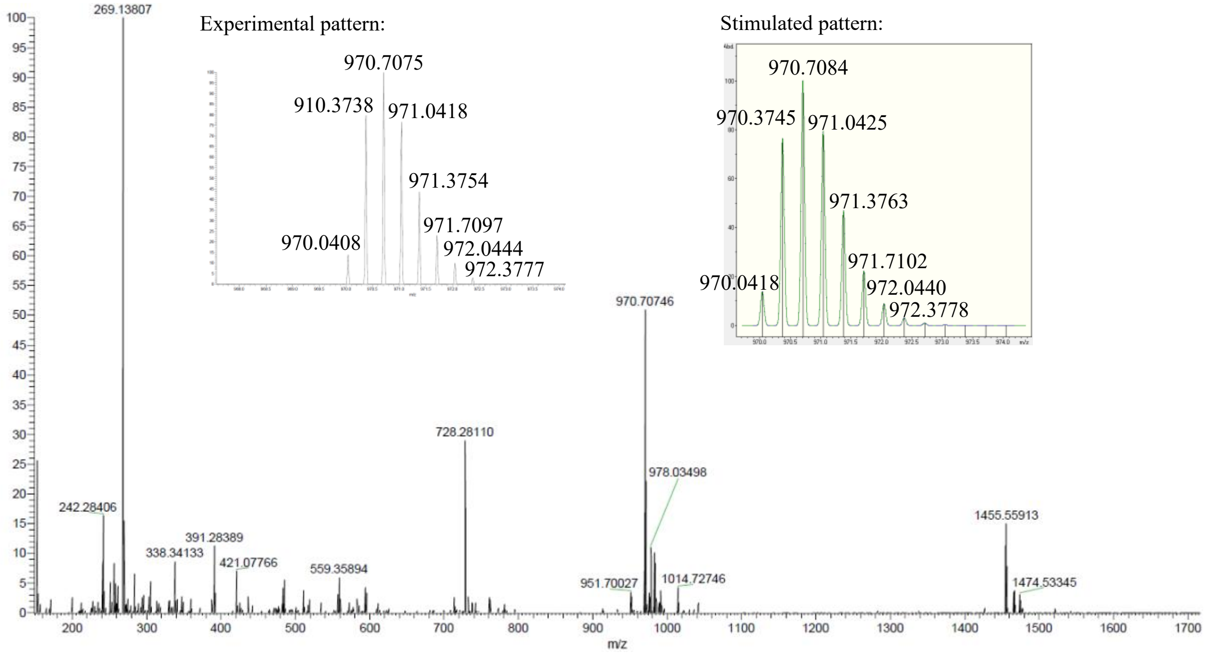

Figure S23. ESI mass spectrum of **13**. The insets show the experimental and simulated isotopic patterns for the $[M+3H]^{3+}$ ion.

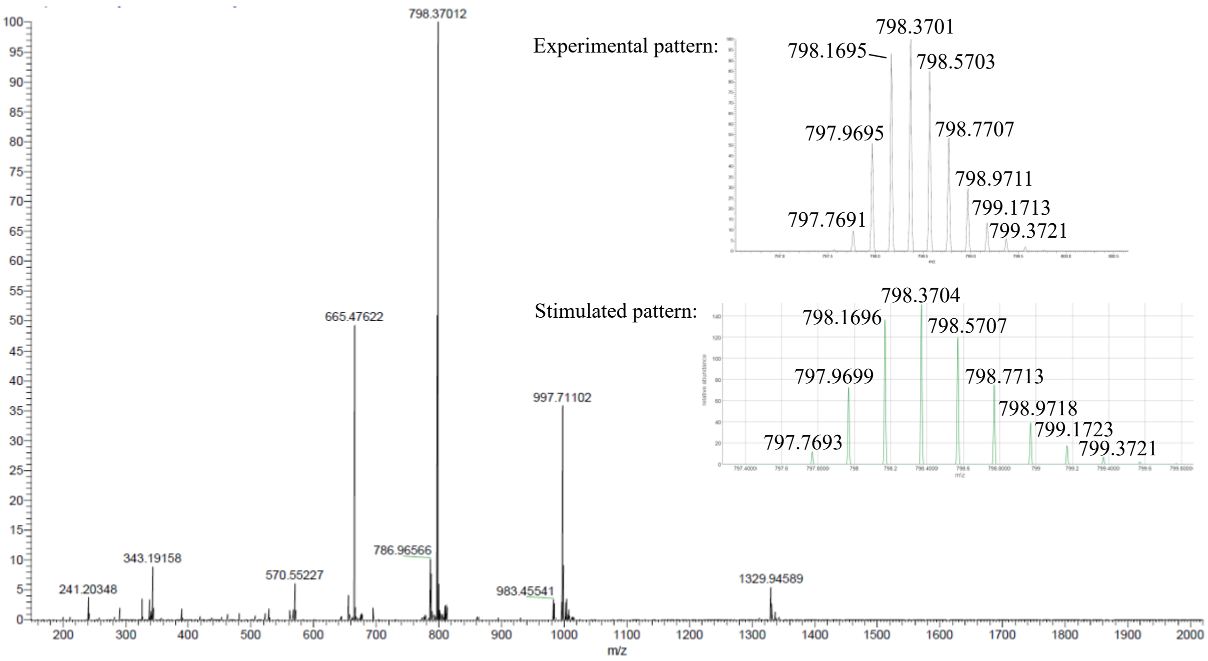

Figure S24. ESI mass spectrum of **1**. The insets show the experimental and simulated isotopic patterns for the $[M+4H]^{5+}$ ion.

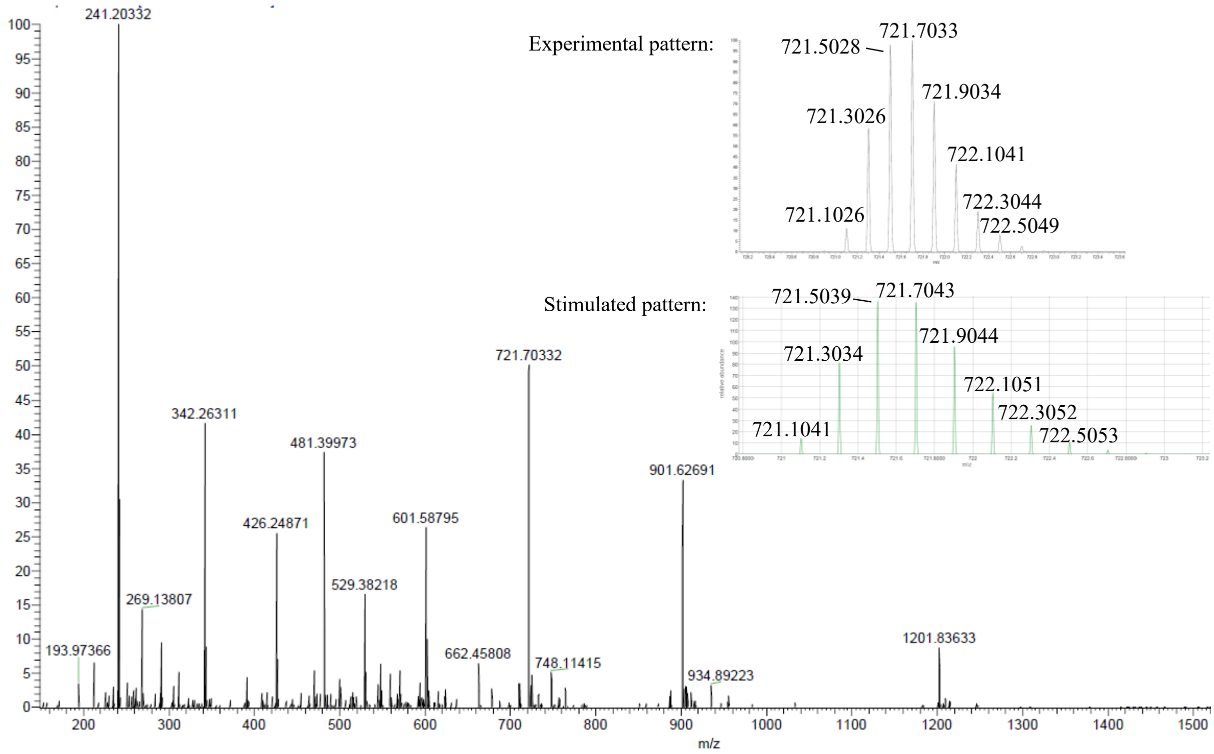

Figure S25. ESI mass spectrum of **14**. The insets show the experimental and simulated isotopic patterns for the $[M+4H]^{5+}$ ion.
